# Alcohol consumption and risk of cancer: a Mendelian randomization analysis of four biobanks and consortium data

**DOI:** 10.1186/s12916-025-04543-8

**Published:** 2025-12-16

**Authors:** Susanna C. Larsson, Amy M. Mason, Héléne T. Cronjé, Emily Bassett, Giovana Horta, Siddhartha Kar, Stephen Burgess

**Affiliations:** 1 Department of Surgical Sciences, Medical Epidemiology, Uppsala University, Uppsala, Sweden; 2 Unit of Cardiovascular and Nutritional Epidemiology, Institute of Environmental Medicine, Karolinska Institutet, Stockholm, Sweden; 3 British Heart Foundation Cardiovascular Epidemiology Unit, Department of Public Health and Primary Care, University of Cambridge, Cambridge, UK; 4 Victor Phillip Dahdaleh Heart and Lung Research Institute, University of Cambridge, Cambridge, UK; 5 Medical Research Council Biostatistics Unit, University of Cambridge, Cambridge, UK; 6 Faculty of Mathematics, University of Michigan, Ann Arbor, MI USA; 7 Department of Oncology, Early Cancer Institute, University of Cambridge, Cambridge, UK; 8 Department of Oncology, Centre for Cancer Genetic Epidemiology, University of Cambridge, Cambridge, UK

**Keywords:** Alcohol consumption, Cancer, Mendelian randomization

## Abstract

**Background:**

Alcohol consumption has been linked to cancer risk. Evidence is strongest for seven cancer types: breast, colorectum, oesophagus, liver, mouth, pharynx, and larynx. However, evidence supporting a causal effect from Mendelian randomization is inconsistent.

**Methods:**

We perform a comprehensive Mendelian randomization analysis to assess whether genetically-predicted alcohol consumption associates with risk of 20 cancers. Such associations would provide supportive evidence for a causal effect of alcohol consumption on cancer risk. We used 95 genetic variants associated with alcohol consumption at genome-wide significance. Primary analyses were conducted in European ancestry participants from UK Biobank (367,643 individuals), FinnGen (500,348 individuals), All of US (169,312 individuals), and Million Veteran Program (451,206 individuals). We also estimated associations in cancer-specific consortia.

**Results:**

No association was observed between genetically-predicted alcohol consumption and overall cancer (odds ratio (OR) per 1 standard deviation increase in alcohol consumption 0.96, *p* = 0.45). Among the seven highlighted cancer types, we saw a multiply-corrected significant positive estimate for combined head/neck cancer (OR 1.51, *p* = 0.001), and nominally significant positive estimates for colorectal (OR 1.21, *p* = 0.035) and oesophageal (OR 1.42 *p* = 0.033) cancer. For liver cancer, there was a null estimate overall (OR 1.40, *p* = 0.10), but a nominally significant positive estimate in Million Veteran Program and when using the *ADH1B*-rs1229984 variant. For breast cancer, there was a null estimate in biobank data (OR 1.09, *p* = 0.25) and consortium data (OR 0.98, *p* = 0.84). Conversely, we observed multiply-corrected significant negative estimates for kidney cancer (OR 0.64, *p* = 0.0003) and endometrial cancer (OR 0.56, *p* = 0.0006), and nominally significant negative estimates for non-Hodgkin’s lymphoma (OR 0.75, *p* = 0.010), myeloma (OR 0.61, *p* = 0.014), and some subtypes of ovarian cancer. There was a nominally significant positive association with cancer mortality (OR 1.44, *p* = 0.003), although this attenuated on adjustment for smoking heaviness. Limitations include potential invalidity of the genetic variants as instruments, limited power, multiple testing, variable cancer detection rates, and unrepresentativeness of the datasets.

**Conclusions:**

We observed moderate-to-weak evidence supporting causal effects of alcohol consumption on risk of head/neck, oesophageal, and colorectal cancer, inconsistent evidence for liver cancer, and no evidence for breast cancer. Overall, human genetic data do not provide evidence that alcohol consumption is a cause of all cancers and suggest there may even be inverse associations with certain cancer types.

**Supplementary Information:**

The online version contains supplementary material available at 10.1186/s12916-025-04543-8.

## Background

A recent report by the United States (US) Surgeon General recommended that alcohol sold in the US is labelled to indicate that alcohol consumption is a cause of cancer [1, 2]. Similar warnings are already required in South Korea [3] and will be mandated in Ireland starting in 2026 [4].

Much of the epidemiological evidence for the relationship between alcohol consumption and cancer risk in humans is observational. The Surgeon General’s report presents mixed messages: on the one hand, it claims that alcohol consumption contributes to 20,000 cancer deaths per year—an estimate based on a wide range of cancers [5]; on the other hand, it states that convincing evidence for a causal effect of alcohol on cancer has only been demonstrated for seven types of cancer, including cancer of the breast (in women), colorectum, oesophagus, liver, mouth (oral cavity), throat (pharynx), and voice box (larynx) [6, 7].

Observational epidemiological studies are limited in their ability to make reliable causal claims [8]. It is well-known that “correlation is not causation”, and many causal claims based on observational associations have failed replication in randomized trials [9, 10]. While there are plausible mechanisms by which alcohol consumption may increase cancer risk [11], several of these are likely to be localized to specific body areas [12]. There are also potential mechanisms by which alcohol consumption may reduce cancer risk [13, 14]. It is unlikely that a large-scale randomized trial to investigate the long-term effects of alcohol consumption would be feasible or ethical to conduct.

Mendelian randomization is an epidemiological approach developed to mitigate against bias from confounding and reverse causation that is pervasive in observational research [15, 16]. In Mendelian randomization, genetic variants specifically related to a particular exposure are used to construct genetically-defined population subgroups with different average levels of the exposure [17]. The independent segregation of alleles at conception means that these genetically-defined subgroups should not differ systematically with respect to confounding variables, giving rise to a natural experiment analogous to a randomized trial [18]. Additionally, genetic variants are fixed at conception, which provides further protection against the influence of environmental confounders and reverse causation [19]. Therefore, compared with conventional observational analyses, Mendelian randomization analyses can provide more reliable insights into causal relationships between risk factors and disease outcomes.

We have previously conducted a Mendelian randomization investigation to assess evidence for a causal effect of alcohol consumption on cancer risk in European ancestry participants from UK Biobank, a large prospective study of UK residents [20]. We did not observe consistent evidence for a harmful effect of alcohol consumption on cancer risk. Here, we expand on our previous analysis in several ways. First, we include five additional years of follow-up in UK Biobank, raising the total number of cancer events considered in that dataset from 75,037 to 99,079. Secondly, we include data from three other large population-based biobanks: FinnGen, All of US, and Million Veteran Program. Thirdly, we include data from relevant cancer-specific consortia. Fourthly, we consider a combined gastrointestinal cancer endpoint. Fifthly, we consider cancer mortality as an outcome. And finally, we consider non-European ancestry data from the All of US and Million Veteran Program biobanks. These developments increase power to detect a causal effect, as well as specificity to address the claims made in the Surgeon General’s report.

## Methods

### Overview of analysis

We perform two-sample Mendelian randomization analyses, taking genetic variants previously shown to be associated with alcohol consumption in a large genome-wide association study (GWAS), and assessing their associations with cancer risk in large biobanks and cancer-specific GWASs. A positive association between genetic predictors of alcohol consumption (or equivalently, genetically-predicted levels of alcohol consumption) and cancer risk would provide supportive evidence for a harmful causal effect of alcohol consumption on cancer risk.

For each outcome, we perform three primary analyses: unadjusted (i.e. univariable) using all variants, adjusted for smoking heaviness (i.e. multivariable with adjustment for lifetime smoking index), and using a variant in the alcohol dehydrogenase 1B (*ADH1B*) gene region only. Analyses using the *ADH1B* variant alone are less vulnerable to bias due to pleiotropy and other sources of instrument invalidity. However, as these estimates are based on a single variant, they are less precise and more variable.

Aside from when our outcome is cancer survival or mortality, we note that our analysis only addresses the question about cancer incidence. We do not primarily investigate whether alcohol consumption increases the severity of outcomes, but only whether it increases risk of having a cancer diagnosis.

If estimates attenuate in multivariable analyses adjusted for smoking heaviness, there are two possible interpretations: either the genetic associations with smoking represent horizontal pleiotropy, or they represent downstream effects of alcohol consumption [21]. In either case, this means that alcohol is not the proximal causal risk factor.

### Biobank datasets

We assess genetic associations with cancer risk in four large biobank datasets: UK Biobank, FinnGen, All of US, and Million Veteran Program.

UK Biobank is a longitudinal cohort study of around 500,000 people from the UK aged 40 to 69 years at baseline, recruited between 2006 and 2010 [22]. Participants were followed up until November 2022 or their date of death. We performed detailed quality control procedures on UK Biobank participants and on genetic variants as described previously, restricting analyses to unrelated participants (that is, more distant than third degree relatives) of European ancestries [23]. Our analyses were performed on 367,643 European ancestry participants. While there are non-European ancestry individuals in UK Biobank, they are relatively few for any specific ancestry group, and so we do not consider these individuals in our analyses.

FinnGen is a large-scale genomics initiative that has analysed over 500,000 Finnish biobank samples and has linked genetic variation with health data to understand disease mechanisms and predispositions [24]. For this work, we used summarized data from data freeze 12, which comprises 500,348 participants overall (282,064 women and 218,284 men). Genome-wide association analyses were performed using Regenie version 2.2.4 [25] and included age, sex, 10 principal components, FinnGen chip version, and genotyping batch as covariates.

All of US is a longitudinal cohort study that launched in 2018 and is still recruiting [26]. This analysis was run on Curated Data Repository v8, which contains whole genome sequencing data on 414,830 people. Participant outcomes are included up to October 2023. Quality control procedures on the participants and genetic variants have been described previously [27]. We restricted analyses to those with electronic health record data, binary sex at birth with cis gender identity, and unrelated individuals (kinship scores < 0.1 between each pair). Primary analyses were conducted on 169,312 European ancestry individuals. We also performed analyses on 56,655 African ancestry individuals and 51,443 admixed American ancestry individuals.

The Million Veteran Program is a longitudinal cohort study established in 2011 [28, 29]. Participants are US military veterans who were recruited through their use of the Department of Veterans Affairs healthcare system. Recruitment prioritized the enrolment of underrepresented groups. Genome-wide association data are available on 635,969 individuals, and primary analyses were performed on up to 451,206 European ancestry individuals [30]. Due to the majority of participants of Million Veteran Program being men (92%), associations with cervical, ovarian, or uterine cancers were not considered in this dataset. Moreover, associations in non-European populations were only available for a subset of cancer types, as only analyses with at least 500 cases were reported. African ancestry analyses were performed on up to 121,793 individuals, and admixed American ancestry analyses were performed on up to 59,869 individuals.

### GWAS consortia

We also consider consortium data for specific outcomes, particularly when large GWAS are available that do not primarily consist of biobank participants (Additional file 1: TableS1).

Genetic associations with breast cancer were obtained from analyses of female European ancestry participants by Zhang et al. [31, 32]. Associations with any breast cancer were estimated in 133,384 cases and 113,789 controls. We also consider associations with breast cancer subtypes: triple negative (~ 4,900 cases), triple negative or BRCA1-positive (18,016 cases), luminal A (~ 57,400 cases), luminal B (~ 13,800 cases), luminal B or HER2-negative (~ 13,800 cases), and HER2-enriched (~ 6,400 cases). Numbers of cases are approximate for some subtypes as the analysis used an imputation algorithm to handle missing tumour data. These datasets did not overlap with any of the biobanks.

Genetic associations with breast cancer survival were obtained from analyses of female European ancestry participants by Escala-Garcia et al. [33, 34] and Morra et al. [35, 36]. Associations with survival for any breast cancer were estimated for 96,661 women with breast cancer who had 7697 events (breast cancer-specific deaths, Escala-Garcia) and for 91,686 women with breast cancer who had 7531 events (Morra). These datasets did not overlap with any of the biobanks.

Genetic associations with ovarian cancer were obtained from analyses of female European ancestry participants by Dareng et al. [37, 38]. For each ovarian cancer subtype, estimates were obtained in analyses with 105,724 controls. We consider associations with non-mucinous epithelial ovarian cancer (23,394 cases), mucinous epithelial ovarian cancer (2587 cases), high-grade serous epithelial ovarian cancer (15,588 cases), low-grade serous epithelial ovarian cancer (2749 cases), endometrioid epithelial ovarian cancer (2877 cases), and clear cell epithelial ovarian cancer (1427 cases). This dataset did not overlap with any of the biobanks.

Genetic associations with endometrial cancer were obtained from analyses of female European ancestry participants by O’Mara et al. [39, 40]. Associations were estimated in 12,906 European ancestry cases and 108,979 European ancestry controls. This dataset contains 636 cases that overlap with UK Biobank.

Genetic associations with prostate cancer were obtained from analyses of male European ancestry participants by Wang et al. [41, 42]. Associations were estimated in 122,188 European ancestry cases and 604,640 European ancestry controls. This dataset contains 8765 cases that overlap with UK Biobank, 6311 that overlap with FinnGen, and 13,649 that overlap with Million Veteran Program.

Genetic associations with kidney cancer were obtained from Purdue et al., a large GWAS consortium [43, 44]. Associations with any kidney cancer were estimated in 25,890 European ancestry cases and 743,585 European ancestry controls. We also consider associations with clear renal cell carcinoma (16,321 cases) and papillary renal cell carcinoma (2193 cases). These datasets contain around 2900 cases that overlap with UK Biobank and FinnGen.

Genetic associations with colorectal cancer were obtained from Fernandez-Rozadilla et al. [45, 46]. Associations were estimated in 78,473 European ancestry cases and 107,143 European ancestry controls. This dataset contains 4800 cases that overlap with UK Biobank.

Genetic associations with oesophageal disease were obtained from Gharahkhani et al. [47, 48]. Associations with oesophageal cancer were estimated in 4112 European ancestry cases and 17,159 European ancestry controls. Associations with Barrett’s oesophagus (a condition that can lead to oesophageal cancer) were estimated in 6167 European ancestry cases and 17,159 European ancestry controls. These datasets do not overlap with any of the biobanks.

### Outcomes

In the biobank datasets, we consider 20 cancer types, primarily defined by cancer site: bladder, brain, breast, cervix, colorectum, head/neck, kidney, leukaemia, liver, lung, melanoma, myeloma, non-Hodgkin’s lymphoma, oesophagus, ovaries, pancreas, prostate, stomach, testes, and uterus. These comprise the most common cancers in these datasets. We also consider a composite “any cancer” outcome and an “any gastrointestinal cancer” outcome, comprising cancer of the bile ducts, colorectum, liver, oesophagus, pancreas, or stomach. Outcomes were defined using hospital episode statistics and death certificates based on International Classification of Diseases (ICD)−9 and ICD-10 codes, and cancer histology data were available (Additional file 1: TableS2). Associations with female-specific cancers (breast, cervix, ovaries, and uterus) were estimated in women only; associations with male-specific cancers (prostate and testes) were estimated in men only.

For FinnGen and Million Veteran Program, we were not able to access individual-participant data and so had to rely on publicly available GWAS data. This means we were not able to conduct analyses for composite outcomes, and the exact case definitions differed for some outcomes (Additional file 1: TableS3–S4).

We also consider cancer mortality as an outcome in UK Biobank, where cancer mortality was defined as having an ICD-9 or ICD-10 code for any cancer listed as a primary cause of death. Disease-specific mortality was only available in All of US for a few hundred individuals and genetic association estimates with disease-specific mortality were not available in FinnGen or Million Veteran Program. In total, 15,583 European ancestry UK Biobank participants had a cancer mortality event, 16,594 had a non-cancer mortality event, and 335,507 survived until the end of the follow-up period.

### Genetic variants used as instruments

Genetic variants were identified in a GWAS comprising 941,280 individuals of European descent that identified 99 variants associated with alcohol consumption at a genome-wide significance level (*p* < 5 × 10^−8^) [49]. Four variants were dropped due to high correlation (*r*^2^ > 0.1) with another identified variant (rs1004787, rs74664784, rs35538052, and rs79139602); for each pair of correlated variants, the variant with the lower beta-coefficient was dropped. The remaining variants have pairwise correlations less than *r*^2^ < 0.1. This threshold was chosen as a reasonable compromise between including highly correlated variants and so essentially double-counting data, and excluding relevant variants and so reducing power. In total, 95 variants associated with alcohol consumption were included in our analyses (Additional file 1: TableS5). The variants explained around 0.2% of the variability in alcohol consumption. This corresponds to an F statistic above 100 in univariable analyses, a conditional F statistic of 14.0 in multivariable analyses adjusted for smoking heaviness, and a conditional F statistic of 41.7 in multivariable analyses adjusted for educational attainment. This GWAS included 311,126 UK Biobank participants and 2666 Finnish individuals from the FinnTwin and Nicotine Addiction Genetics Finland (NAG-FIN) studies (neither of these studies was included in FinnGen, but some individuals in these studies may have also been recruited into FinnGen). In FinnGen, 5 of the 95 variants were not available, of which 3 were replaced with proxies (*r*^2^ > 0.95 for all proxies, Additional file 1: TableS6). In All of US, 1 of the variants was not available (rs378421). In Million Veteran Program, 1 of the variants was not available (rs2532276). In the consortium data, up to 3 variants were unavailable depending on the consortium. The same genetic variants were used in non-European ancestry analyses.

Additionally, we also perform analyses using rs1229984, a genetic variant in the *ADH1B* gene region that encodes alcohol dehydrogenase 1B, an enzyme that catalyses the oxidation of alcohol to acetaldehyde.

### Statistical methods

We obtained cancer genetic association estimates for UK Biobank and All of US by logistic regression within each dataset with adjustment for age, age-squared, sex, and 10 genomic principal components (5 principal components in All of US). Individuals with the outcome of interest were treated as cases, and individuals with no cancer outcome were regarded as controls. Individuals with a different cancer diagnosis were excluded from the analysis of other cancer types. For cancer mortality, individuals who survived until the end of the follow-up period were regarded as controls, and individuals who had a non-cancer mortality event were excluded from analyses. Genetic association estimates for FinnGen, Million Veteran Program, and GWAS consortia were estimated similarly, but we only had access to published summarized data. In Million Veteran Program, individuals without the specific cancer diagnosis were treated as controls.

Primary Mendelian randomization analyses within each dataset were performed using the inverse-variance weighted method with a random-effects model [50]. We additionally used the weighted median, MR-Egger, Mendelian Randomization Pleiotropy RESidual Sum and Outlier (MR-PRESSO), and contamination mixture methods [51, 52]. Estimates were combined across datasets using fixed-effect meta-analysis.

To mitigate against potential pleiotropic effects via cigarette smoking [53], we additionally performed multivariable Mendelian randomization adjusting for genetic associations with lifetime smoking index, estimated in 462,690 European ancestry UK Biobank participants [54, 55]. We note that the *ADH1B* variant is not associated with lifetime smoking index (*p* = 0.21) [54]. Additionally, we conducted multivariable Mendelian randomization adjusting for genetic associations with educational attainment, estimated in 753,152 individuals of European ancestry [56, 57]. On request from a reviewer, we also conducted multivariable Mendelian randomization analyses in UK Biobank for uterine and kidney cancer adjusted for genetic associations with body mass index, estimated in 806,834 individuals of European ancestry [58, 59].

For simplicity of presentation, we describe results which have a two-sided *p*-value below 0.05/20 = 0.0025 as significant on correction for multiple testing (based on 20 cancer types), results with a *p*-value less than 0.05 as nominally significant, and *p*-values greater than 0.05 as null. We note that consideration of nominally significant results may lead to false positive claims, as it ignores multiple testing. Our priority in this analysis was to detect whether there was any evidence supporting a causal effect of alcohol consumption; hence, we use a low evidential threshold. We also note that power to detect a causal effect varies strongly between outcomes, as the number of cases and controls varies between datasets.

Unless otherwise stated, analyses were performed using R version 4.3.3 (“Angel Food Cake”) and MendelianRandomization package version 0.10.0.

## Results

### Biobank datasets and cancer types

**Fig. 1:**
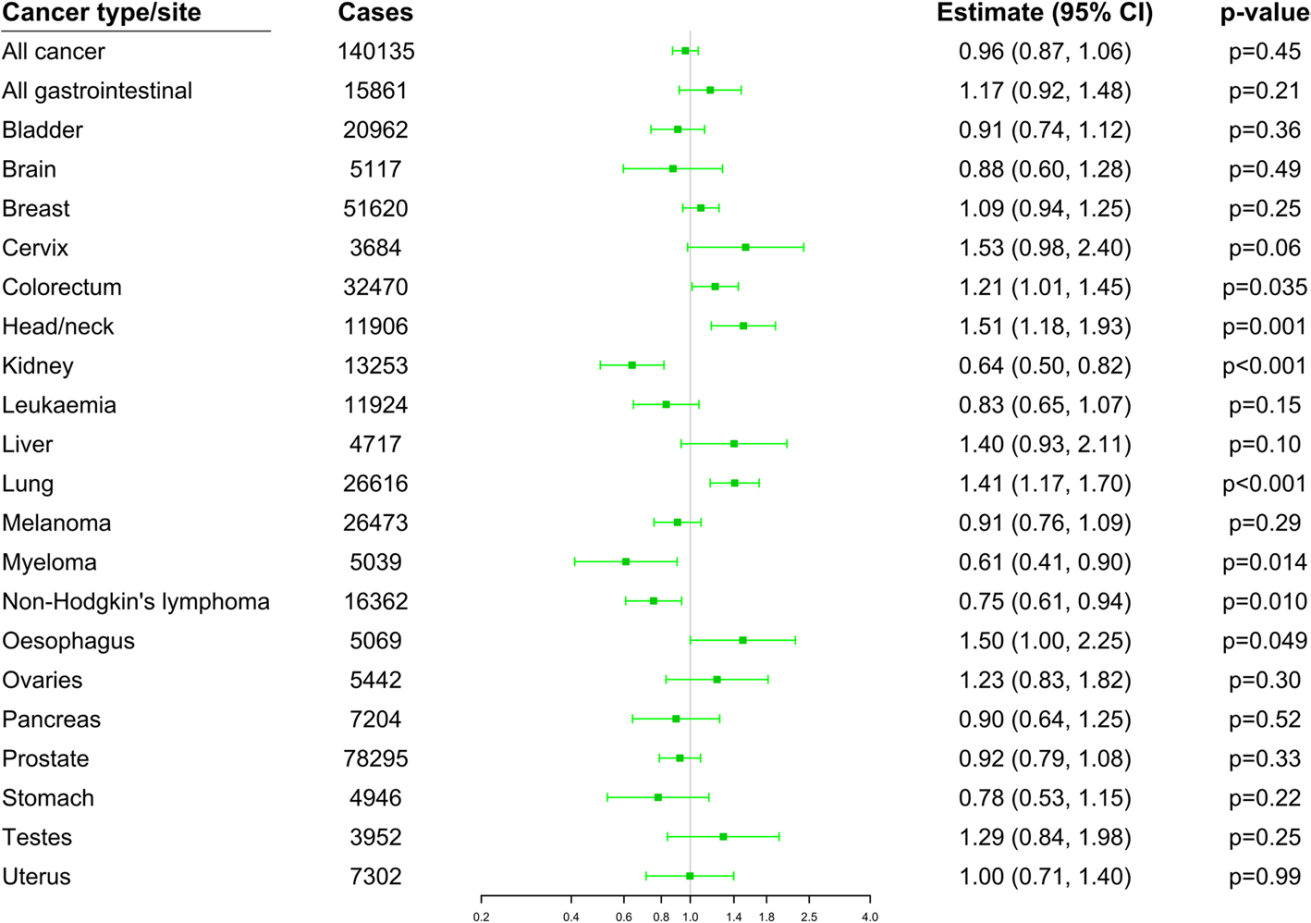
Combined Mendelian randomization estimates calculated using outcome data from all four biobank datasets. Results are from univariable analysis based on all genetic variants. Estimates represent odds ratios per 1 standard deviation increase in log-transformed number of drinks per week. Error bars are 95% confidence intervals

Associations between genetically-predicted alcohol consumption and cancer outcomes in European ancestry participants, derived from combined biobank datasets, are presented in Fig. 1 and Table 1. Corresponding estimates for African ancestry individuals are shown in Additional file 2: Fig. S1 (All of US) and Fig. S2 (Million Veteran Program), while those for admixed American ancestry individuals are displayed in Additional file 2: Fig. S3 (All of US) and Fig. S4 (Million Veteran Program). Estimates are expressed as odds ratios (OR) with 95% confidence intervals (CI) per 1 standard deviation increase in the log-transformed number of drinks consumed per week.

**Table 1:** Comparison of Mendelian randomization estimates calculated using outcome data for all four biobank datasets from three approaches

	**Unadjusted**			**Adjusted for smoking**			***ADH1B* variant only**		
Cancer type/site	Estimate	95% CI	*p*-value	Estimate	95% CI	*p*-value	Estimate	95% CI	*p*-value
All cancer	0.96	0.87, 1.06	0.45	0.89	0.80, 0.99	**0.031**	1.01	0.85, 1.19	0.95
All gastrointestinal	1.17	0.92, 1.48	0.21	1.10	0.84, 1.43	0.50	1.01	0.66, 1.56	0.96
Bladder	0.91	0.74, 1.12	0.36	0.78	0.62, 0.98	**0.030**	0.69	0.47, 1.03	0.07
Brain	0.88	0.60, 1.28	0.49	0.92	0.60, 1.42	0.72	0.69	0.30, 1.63	0.40
Breast	1.09	0.94, 1.25	0.25	1.02	0.88, 1.19	0.75	1.14	0.83, 1.55	0.42
Cervix	1.53	0.98, 2.40	0.06	1.34	0.81, 2.20	0.25	1.73	0.61, 4.87	0.30
Colorectum	1.21	1.01, 1.45	**0.035**	1.23	1.01, 1.50	**0.039**	1.47	1.04, 2.08	**0.028**
Head/neck	1.51	1.18, 1.93	***0.001***	1.40	1.06, 1.85	**0.017**	1.91	1.08, 3.37	**0.025**
Kidney	0.64	0.50, 0.82	***<0.001***	0.60	0.45, 0.78	***<0.001***	0.73	0.44, 1.20	0.21
Leukaemia	0.83	0.65, 1.07	0.15	0.81	0.62, 1.08	0.15	0.98	0.60, 1.60	0.94
Liver	1.40	0.93, 2.11	0.10	1.11	0.71, 1.74	0.65	2.26	1.01, 5.06	**0.048**
Lung	1.41	1.17, 1.70	***<0.001***	1.04	0.86, 1.26	0.68	1.17	0.82, 1.67	0.38
Melanoma	0.91	0.76, 1.09	0.29	0.86	0.70, 1.05	0.13	0.75	0.52, 1.08	0.12
Myeloma	0.61	0.41, 0.90	**0.014**	0.65	0.42, 1.01	0.05	0.41	0.18, 0.93	**0.033**
Non-Hodgkin's lymphoma	0.75	0.61, 0.94	**0.010**	0.69	0.54, 0.88	***0.002***	1.11	0.71, 1.73	0.64
Oesophagus	1.50	1.00, 2.25	**0.049**	1.32	0.84, 2.09	0.23	1.72	0.73, 4.07	0.22
Ovaries	1.23	0.83, 1.82	0.30	1.03	0.66, 1.60	0.89	0.49	0.21, 1.13	0.10
Pancreas	0.90	0.87, 1.06	0.45	0.98	0.68, 1.43	0.94	1.23	0.56, 2.69	0.60
Prostate	0.92	0.92, 1.48	0.21	0.93	0.78, 1.11	0.44	1.16	0.91, 1.49	0.23
Stomach	0.78	0.74, 1.12	0.36	0.59	0.38, 0.92	**0.020**	0.66	0.25, 1.74	0.41
Testes	1.29	0.60, 1.28	0.49	1.40	0.86, 2.26	0.17	2.35	0.92, 6.00	0.08
Uterus	1.00	0.94, 1.25	0.25	0.91	0.62, 1.34	0.64	1.06	0.48, 2.32	0.88

Estimates represent odds ratios per 1 standard deviation increase in log-transformed number of drinks per week. *P*-values less than 0.05 (nominally significant) are shown in bold, *p*-values less than 0.0025 (multiple-corrected threshold) are shown in bold-italic

In primary analyses across the four biobanks, positive estimates were observed for colorectal cancer (OR 1.21, 95% CI 1.01, 1.45, *p* = 0.035); head/neck cancer (OR 1.51, 95% CI 1.18, 1.93, *p* = 0.001); lung cancer (OR 1.41, 95% CI 1.17, 1.70, *p* = 0.0004); and oesophageal cancer (OR 1.50, 95% CI 1.00, 2.25, *p* = 0.049), while negative estimates were found for kidney cancer (OR 0.64, 95% CI 0.50, 0.82, *p* = 0.0003); myeloma (OR 0.61, 95% CI 0.41, 0.90, *p* = 0.014); and non-Hodgkin’s lymphoma (OR 0.75, 95% CI 0.61, 0.94, *p* = 0.010) (Fig. 1).

When accounting for smoking in multivariable Mendelian randomization analysis (Table 1), estimates for lung cancer attenuated sharply: OR 1.04 (95% CI 0.86, 1.26, *p* = 0.68). Otherwise, all associations remained with the exception of oesophageal cancer, for which the association attenuated somewhat (OR 1.32, 95% CI 0.84, 2.09, *p* = 0.23), and myeloma, where the estimate attenuated slightly (OR 0.65, 95% CI 0.42, 1.01, *p* = 0.05). There were additionally negative estimates for stomach cancer (OR 0.59, 95% CI 0.38, 0.92, *p* = 0.020) and bladder cancer (OR 0.78, 95% CI 0.62, 0.98, *p* = 0.030). Results from multivariable Mendelian randomization analysis adjusting for genetically predicted educational attainment (Additional File 1: Table S7) and body mass index (Additional File 1: Table S8) were consistent with the unadjusted results.

Estimates were similar but less precise when using the *ADH1B* variant only (Table 1). Positive estimates were observed for colorectal and head/neck, and additionally for liver cancer. The estimate for lung cancer was substantially attenuated. The only negative association was for myeloma.

Estimates from primary analyses in each biobank in turn are presented in Additional file 2: Figs. S5–S8. A notable biobank-specific result was a positive association with liver cancer in Million Veteran Program (OR 1.94, 95% 1.14, 3.30, *p* = 0.014).

### Biobank datasets and composite outcomes

There was no clear association between genetically-predicted alcohol consumption and risk of any cancer or any gastrointestinal cancer for European ancestry participants in the UK Biobank or All of US in the primary analysis (Table 1): combined estimate for any cancer OR 0.96 (95% CI 0.87, 1.06, *p* = 0.45), combined estimate for any gastrointestinal cancer OR 1.17 (95% CI 0.92, 1.48, *p* = 0.21). Similar estimates were obtained when using the *ADH1B* variant only, with an OR of 1.01 (95% CI 0.85, 1.19, *p* = 0.95) for any cancer and 1.01 (95% CI 0.66, 1.56, *p* = 0.96) for any gastrointestinal cancer. When adjusting for smoking heaviness, the estimate for any gastrointestinal cancer was null (OR 1.10, 95% CI 0.84, 1.43, *p* = 0.50), but the estimate for any cancer was negative (OR 0.89, 95% CI 0.80, 0.99, *p* = 0.031).

No associations were observed with composite outcomes for African ancestry participants in All of US: any cancer OR 0.79 (95% CI 0.49, 1.26, *p* = 0.31); any gastrointestinal cancer OR 0.84 (95% CI 0.31, 2.25, *p* = 0.72), or for admixed American participants in All of US: any cancer OR 1.09 (95% CI 0.78, 1.53, *p* = 0.62); any gastrointestinal cancer OR 0.87 (95% CI 0.38, 1.99, *p* = 0.74).

Genetically-predicted alcohol consumption was associated with cancer mortality in the UK Biobank (OR 1.44, 95% CI 1.13, 1.84, *p* = 0.003). This association attenuated on adjustment for smoking (OR 1.18, 95% CI 0.91, 1.52, *p* = 0.20), but was larger when using the *ADH1B* variant only (OR 1.68, 95% CI 1.02, 2.79, *p* = 0.043).

### GWAS consortia

**Fig. 2:**
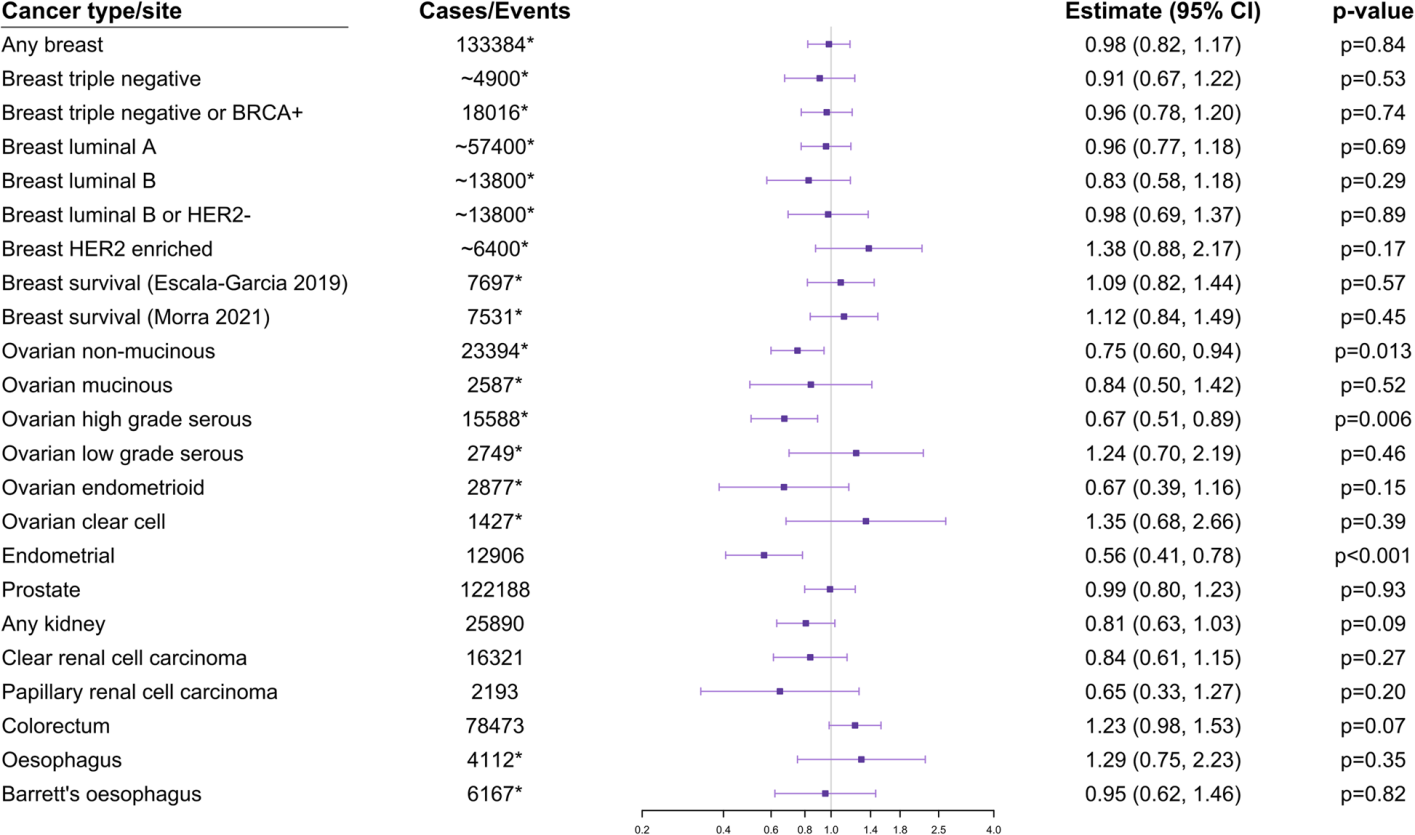
Mendelian randomization estimates calculated using consortium data.Results are from univariable analysis based on all genetic variants. Estimates represent odds ratios (hazard ratios for breast cancer survival) per 1 standard deviation increase in log-transformed number of drinks per week. Error bars are 95% confidence intervals. *Indicates no overlap in participants with biobank datasets

Results based on data from the GWAS consortia are shown in Fig. 2 and Table 2. As before, estimates represent ORs per 1 standard deviation increase in log-transformed number of drinks consumed per week. For breast cancer survival, estimates represent hazard ratios per standard deviation increase in log-transformed number of drinks consumed per week.

**Table 2:** Comparison of Mendelian randomization estimates calculated using outcome data for consortium data from three approaches

	**Unadjusted**			**Adjusted for smoking**			***ADH1B* variant only**		
Cancer type/site	Estimate	95% CI	*p*-value	Estimate	95% CI	*p*-value	Estimate	95% CI	*p*-value
Any breast	0.98	0.82, 1.17	0.84	0.96	0.79, 1.15	0.64	0.86	0.69, 1.06	0.16
Breast triple negative	0.91	0.67, 1.22	0.53	0.95	0.69, 1.33	0.78	0.81	0.50, 1.31	0.38
Breast triple negative or BRCA+	0.96	0.78, 1.20	0.74	0.98	0.77, 1.25	0.89	1.01	0.72, 1.43	0.95
Breast luminal A	0.96	0.77, 1.18	0.69	0.98	0.78, 1.22	0.84	0.93	0.71, 1.22	0.62
Breast luminal B	0.83	0.58, 1.18	0.29	0.74	0.51, 1.07	0.11	0.50	0.29, 0.86	**0.013**
Breast luminal B or HER2-	0.98	0.69, 1.37	0.89	0.97	0.66, 1.41	0.86	0.89	0.55, 1.46	0.65
Breast HER2 enriched	1.38	0.88, 2.17	0.17	1.40	0.86, 2.27	0.17	1.70	0.74, 3.93	0.21
Breast survival (Escala-Garcia 2019)	1.09	0.82, 1.44	0.57	1.10	0.80, 1.51	0.55	1.00	0.57, 1.75	0.99
Breast survival (Morra 2021)	1.12	0.84, 1.49	0.45	1.24	0.90, 1.70	0.19	0.94	0.54, 1.65	0.83
Ovarian non-mucinous	0.75	0.60, 0.94	**0.013**	0.81	0.65, 0.99	**0.045**	0.62	0.42, 0.90	**0.012**
Ovarian mucinous	0.84	0.50, 1.42	0.52	0.85	0.48, 1.50	0.56	1.07	0.36, 3.18	0.91
Ovarian high grade serous	0.67	0.51, 0.89	**0.006**	0.74	0.58, 0.95	**0.020**	0.49	0.32, 0.75	***0.001***
Ovarian low grade serous	1.24	0.70, 2.19	0.46	1.10	0.58, 2.06	0.78	1.44	0.52, 3.97	0.48
Ovarian endometrioid	0.67	0.39, 1.16	0.15	0.81	0.45, 1.46	0.48	1.47	0.54, 3.95	0.45
Ovarian clear cell	1.35	0.68, 2.66	0.39	1.27	0.60, 2.66	0.54	0.49	0.13, 1.92	0.31
Endometrial	0.56	0.41, 0.78	***<0.001***	0.55	0.38, 0.80	***0.001***	0.54	0.30, 0.98	**0.043**
Prostate	0.99	0.80, 1.23	0.93	0.99	0.78, 1.26	0.95	1.20	0.96, 1.49	0.10
Any kidney	0.81	0.63, 1.03	0.09	0.79	0.60, 1.03	0.08	1.11	0.75, 1.62	0.61
Clear renal cell carcinoma	0.84	0.61, 1.15	0.27	0.80	0.57, 1.11	0.18	1.35	0.84, 2.18	0.21
Papillary renal cell carcinoma	0.65	0.33, 1.27	0.20	0.75	0.37, 1.53	0.43	1.00	0.32, 3.16	1.00
Colorectum	1.23	0.98, 1.53	0.07	1.26	0.99, 1.60	0.06	1.33	1.04, 1.70	**0.023**
Oesophagus	1.29	0.75, 2.23	0.35	1.20	0.66, 2.19	0.54	0.82	0.29, 2.32	0.71
Barrett's oesophagus	0.95	0.62, 1.46	0.82	0.86	0.54, 1.39	0.55	1.11	0.47, 2.65	0.81

Estimates represent odds ratios (hazard ratios for breast cancer survival) per 1 standard deviation increase in log-transformed number of drinks per week. *P*-values less than 0.05 (nominally significant) are shown in bold, *p*-values less than 0.0025 (multiple-corrected threshold) are shown in bold-italic

There was no association of genetically-predicted alcohol consumption with breast cancer overall (OR 0.98, 95% CI 0.82, 1.17, *p* = 0.84), for any subtype of breast cancer or for breast cancer survival. There was a negative estimate for luminal B breast cancer based on the *ADH1B* variant only. There were negative associations for non-mucinous ovarian cancer (OR 0.75, 95% CI 0.60, 0.94, *p* = 0.013) and high-grade serous epithelial ovarian cancer (OR 0.67, 95% CI 0.51, 0.89, *p* = 0.006) and null associations for other ovarian cancer subtypes. There was a negative association with endometrial cancer (OR 0.56, 95% 0.41, 0.78, *p* = 0.0006) and a null association with prostate cancer (OR 0.99, 95% CI 0.80, 1.23, *p* = 0.93). The negative associations for ovarian cancer subtypes and endometrial cancer remained after adjusting for smoking heaviness and in analyses based on the *ADH1B* variant only. Likewise, adjusting for educational attainment did not alter the results materially (Additional file 1: TableS9).

There was no clear association with kidney cancer, although the estimate was in the inverse direction: OR 0.81 (95% CI 0.63, 1.03, *p* = 0.09). Similar estimates were observed for clear renal cell carcinoma (OR 0.84, 95% CI 0.61, 1.15, *p* = 0.27) and papillary cell renal cell carcinoma (OR 0.65, 95% CI 0.33, 1.27, *p* = 0.20). There was no clear association with colorectal cancer, but the estimate was in the harmful direction: OR 1.23 (95% CI 0.98, 1.53, *p* = 0.07). These estimates are not independent from the biobank estimates, as they contain case participants from the UK Biobank (kidney and colorectal) and FinnGen (kidney).

There was no clear association with oesophageal cancer in the consortium data, although the estimate was in the harmful direction: OR 1.29, 95% CI 0.75, 2.23, *p* = 0.35. This estimate is independent of the biobank estimates. Combining with the biobank estimates for oesophageal cancer, we obtain an overall meta-analysis OR estimate of 1.42 (95% CI 1.03, 1.97, *p* = 0.033). By contrast, the estimate for Barrett’s oesophagus was null (OR 0.95, 95% CI 0.62, 1.46, *p* = 0.82).

### Supplementary analyses

Results from the weighted median, MR-Egger, and contamination mixture methods as well as heterogeneity test statistics are provided in Additional file 1: Tables S10–S13 for biobank datasets, and in Additional file 1: Table S14 for consortia data. Estimates were generally consistent with those obtained from the primary inverse-variance weighted analysis, although typically less precise. In notable discrepancies from primary analyses, there was stronger evidence for a harmful effect of alcohol consumption on colorectal cancer, there was some evidence for a harmful effect on prostate cancer, and there was evidence for a protective effect on luminal B breast cancer. We also performed the MR-PRESSO method, which detected no outliers in any analysis. Consequently, the estimates were identical to those in the primary analysis. We further performed the debiased inverse-variance weighted method in UK Biobank, to account for potential bias due to sample overlap [60]. Estimates from this method only differed from those in the primary analysis in the second decimal place.

Estimates of between-study heterogeneity in estimates are provided in Additional file 1: Table S15. No outcomes had excess heterogeneity (p_het_ < 0.05) for analyses based on the wider set of genetic variants, whether univariable or multivariable. There was some excess heterogeneity in analyses based the *ADH1B* variant only, although this was not extreme for any outcome.

Scatterplots of the genetic associations for the consortium data are presented in Additional file 2: Figs. S9–S31. The variant having the strongest association with alcohol consumption (on the far right-hand side of the scatterplots) is the variant in the *ADH1B* gene region (rs1229984).

## Discussion

In this paper, we assessed the evidence for a causal relationship between alcohol consumption and cancer risk in a Mendelian randomization framework. Table 3 provides a summary of our findings and the evidence from meta-analyses of observational studies as a comparison [7, 61–73]. We found no evidence of a harmful effect of alcohol consumption on overall cancer incidence, but the genetic association with cancer mortality was positive. Of the seven cancers listed in the US Surgeon General’s report as convincingly linked to alcohol, we saw evidence supporting a harmful effect of alcohol consumption on head/neck cancer (our definition includes mouth, pharynx, and larynx cancers), oesophageal cancer, and colorectal cancer at a nominal level of statistical significance in the primary analysis. Moreover, we found evidence supporting a harmful effect of alcohol consumption on liver cancer in the analysis utilizing the *ADH1B*-rs1229984 genetic variant only. Conversely, estimates for breast cancer were compatible with the null in all analyses.

**Table 3:** Comparison of results from observational studies and the present Mendelian randomization study on alcohol consumption and cancer

	**Previous evidence from observational studies**^**a**^		**Evidence from the present Mendelian randomization analyses**					
			**Combined across biobanks**			**Consortium data**		
**Cancer type/site**	**Direction of the association**	**References**	**Unadjusted**	**Smoking adjusted**	***ADH1B* only**	**Unadjusted**	**Smoking adjusted**	***ADH1B* only**
Bladder	–	[7, 61]	–	↓	–	N/A	N/A	N/A
Brain	–	[61]	–	–	–	N/A	N/A	N/A
Breast	↑↑	[7, 61, 62]	–	–	–	–	–	–
Cervix	–	[7, 61]	–	–	–	N/A	N/A	N/A
Colorectum	↑↑	[7, 61, 64]	↑	↑	↑	–	–	↑
Head/neck (mouth, pharynx, and larynx)	↑↑	[7, 61]	↑↑	↑	↑	N/A	N/A	N/A
Kidney	↓↓	[7, 61, 65, 72]	↓↓	↓↓	–	–	–	–
Leukaemia	–	[61, 70]	–	–	–	N/A	N/A	N/A
Liver	↑↑	[7, 61]	–	–	↑	N/A	N/A	N/A
Lung	↑	[7, 61]	↑↑	–	–	N/A	N/A	N/A
Melanoma	↑	[7, 61]	–	–	–	N/A	N/A	N/A
Myeloma	↓	[71]	↓	–	↓	N/A	N/A	N/A
Non-Hodgkin’s lymphoma	↓	[61, 70]	↓	↓↓	–	N/A	N/A	N/A
Oesophagus	↑↑	[7, 61]	↑	–	–	–	–	–
Ovaries	–	[7, 61, 69]	–	–	–	–	–	–
Pancreas	↑	[7, 61, 63, 66]	–	–	–	N/A	N/A	N/A
Prostate	↑	[7, 61, 67]	–	–	–	–	–	–
Stomach	↑	[7, 61, 68]	–	↓	–	N/A	N/A	N/A
Testes	N/A	N/A	–	–	–	N/A	N/A	N/A
Uterus/endometrium	–	[7, 61]	–	–	–	↓↓	↓↓	↓

^a^Studies were identified through a literature search in PubMed, focusing on meta-analyses of observational studies published in the past two decades. A formal systematic review protocol was not applied. Two arrows indicate moderate to strong evidence of an association in observational studies, or an association at *p* < 0.0025 (multiple-corrected threshold) in the Mendelian randomization analysis. One arrow indicates suggestive evidence of an association in observational studies, or an association at *p* < 0.05 in the Mendelian randomization analysis. A minus indicates moderate to strong evidence of no association in observational studies, or an association at *p* > 0.05 in the Mendelian randomization analysis. N/A indicates not available

The positive associations of genetically-predicted alcohol consumption with cancer mortality, oesophageal cancer, and lung cancer attenuated, and became fully null for lung cancer, upon adjustment for smoking heaviness. A potential explanation for this finding is that alcohol consumption is culturally linked to cigarette smoking, such that alcohol drinking leads to an increased propensity to smoke. Hence, there may be an indirect causal effect of alcohol consumption on lung cancer, but smoking is the direct causal risk factor. The lack of genetic association with lung cancer in the All of US study may be a chance finding but may also reflect differences in the underlying relationships between alcohol consumption and smoking culture among the study populations.

Our present findings based on four large databases and consortia data agree with those from our previous Mendelian randomization investigation [20] and other Mendelian randomization studies based on the UK Biobank or consortia data [74]. Similarly, Mendelian randomization analyses in the China Kadoorie Biobank have indicated a harmful effect of alcohol consumption in a composite analysis for risk of a subset of alcohol-related cancers (cancers of the lip, oral cavity, pharynx, larynx, oesophagus, liver, colorectum, and female breast), but no effect on other (“non-alcohol-related”) cancers [75]. Another analysis in this dataset showed genetic associations of alcohol related variants with head/neck cancer and oesophageal cancer, but no clear associations with liver, colorectal, stomach, or lung cancer, although the number of cases was less than 1200 for each of these cancer types [76]. Mendelian randomization analyses for alcohol consumption in East Asian populations are particularly valuable, as genetic variants in these populations explain a much higher proportion of variance in alcohol consumption, and so analyses can be undertaken solely based on variants in gene regions with known mechanistic links to alcohol metabolism.

The lack of association between alcohol consumption and breast cancer risk in our Mendelian randomization analyses contrasts with meta-analyses of observational studies, which show a 5% to 10% increase in breast cancer risk per 10 g/day increment of alcohol consumption [7, 62]. This inconsistency may be due to residual confounding in observational studies. Alternatively, our analyses may have lacked the statistical power to detect the modest association observed in these studies.

There is suggestive evidence from observational studies that alcohol consumption may modestly increase the risk of melanoma [7, 61] as well as stomach [7, 61, 68], pancreatic [7, 61, 63, 66], and prostate cancer [61, 67]. Our analyses and previous Mendelian randomization investigations [74] do not support a harmful effect of alcohol consumption on these cancers.

The strongest associations we observed were in the protective direction. Specifically, we found evidence showing inverse associations between genetically-predicted alcohol consumption and kidney cancer, non-Hodgkin’s lymphoma, myeloma, endometrial cancer, and certain subtypes of ovarian cancer. These findings are consistent with observational epidemiological studies that have reported inverse associations for kidney cancer [7, 61, 65, 72], non-Hodgkin’s lymphoma [61, 70], and myeloma [73], but no association with endometrial or ovarian cancer [61, 69]. Although we are generally sceptical about the value of observational research in making causal claims, the positive observational associations of alcohol consumption with most outcomes make these negative associations notable. We may expect bias from confounding to influence estimates for different cancer types in a consistent direction. Hence, we would interpret these observational associations as further evidential support that there might be a protective effect of alcohol consumption on these cancer types. It is notable that the positive observational association between alcohol consumption and risk of breast cancer [7, 61, 62] was not supported in our analyses, but these less common cancer types with negative observational associations were corroborated. Although the observed inverse associations are generally consistent across sensitivity analyses and study designs, they should be interpreted with caution. Potential sources of bias, such as pleiotropy, residual confounding, health selection, and under-diagnosis, cannot be excluded. Moreover, while some hypotheses suggest that moderate alcohol consumption may lower the risk of certain cancers by reducing fasting insulin levels and the risk of type 2 diabetes [77], or through anti-carcinogenic properties of phenolic compounds found in wine and beer [13, 14], these mechanisms remain poorly understood and warrant further investigation.

However, we want to leave readers in no doubt that alcohol consumption increases the risk of many diseases. Previous wide-angled Mendelian randomization analyses have provided evidence for harmful effects on a variety of outcomes in both Chinese and European populations [78, (Kassaw NA, Zhou A, Stacey D, Mulugeta A, Lee SH, Burgess S, Hyppӧnen E: Phenome-wide study on alcohol consumption provides genetic evidence for a causal association with multiple diseases and biomarkers, Under review)]. Mendelian randomization analyses suggest a positive linear association between alcohol consumption and risk of mortality from all causes, cardiovascular disease, and digestive diseases [79]. Non-linear Mendelian randomization provides no evidence for a beneficial effect of alcohol consumption on cardiovascular disease risk at any level [80, 81]. A prior Mendelian randomization analysis in UK Biobank also provided evidence for a harmful effect of alcohol consumption on cancer mortality [82], aligning with our results. We observed inconsistency in this finding, as the estimate attenuated upon adjustment for smoking heaviness, but not when restricting to the *ADH1B* variant, which is not associated with lifetime smoking index.

There are many caveats and limitations to this research. Indeed, we tend to find that readers of our papers on Mendelian randomization are less sceptical about our findings than we are ourselves. Some limitations concern the genetic variants used in our analyses. The genetic variants may not be valid instrumental variables. They may have pleiotropic effects on other variables that influence alcohol consumption, or they may be subject to bias from assortative mating or population stratification [83]. However, these phenomena would typically bias estimates away from the null [52], whereas here we observe largely null findings, particularly for more common cancer outcomes. Genetic variants may explain only a small proportion of variance in alcohol consumption. However, the same variants have been able to support causal effects of alcohol consumption on several outcomes in UK Biobank data with similar numbers of events and hence similar statistical power [84].

The biological relevance of the genetic variants to alcohol consumption is not clear, although results were similar (but less precise) when only considering the lead variant in the *ADH1B* gene region. Additionally, it is unclear whether the genetic variants mimic relevant changes in alcohol consumption behaviour. In particular, we are unable to distinguish between consumption of different alcohol types and between regular drinking versus episodic or excessive drinking. Capturing such nuanced patterns of alcohol use would require distinct genetic predictors of alcohol drinking behaviours, which are implausible to exist.

Other limitations relate to the interpretation of our estimates. Our analyses assume a linear model, meaning that the estimates represent population-averaged effects of a shift in the distribution of alcohol consumption [85]. This approach does not allow us to assess the shape of the causal relationship or to identify any threshold level in the effect of alcohol consumption on cancer risk. While methods for non-linear Mendelian randomization have been developed, estimates in strata of the population with low levels of alcohol consumption are imprecise, as genetic associations with alcohol consumption in light drinkers are, by necessity, small in magnitude. Hence, non-linear analyses typically do not provide sufficient power to be informative about the harms of alcohol consumption at low consumption levels. Additionally, such analyses require individual-participant data and so could only be performed in UK Biobank (sufficiently detailed alcohol measurements in All of US are not available). For this reason, we do not perform non-linear analyses here, as they would likely be uninformative.

We also did not account for multiple testing in our analyses. Overall associations with head/neck, kidney, and endometrial cancer would be considered significant accounting for correction for 20 outcomes. While nominally significant associations in overall analyses provide some evidence supporting a causal effect of alcohol, we remain cautious about making strong claims in these cases. In contrast, we would regard associations seen in one dataset but not in another, or using one method but not another, as dubious.

A further limitation is that we could not examine the association between alcohol consumption and histological types of oesophageal cancer. Alcohol consumption is strongly associated with an increased risk of squamous cell oesophageal cancer but not oesophageal adenocarcinoma [7, 61], the more common type in European descent populations.

As we could only assess the diagnosis of cancer, it is possible that alcohol consumption increases the risk of undiagnosed cancer. It may be that some of our negative estimates reflect an inverse effect of alcohol on being diagnosed with cancer. In addition to this, we primarily assess the impact of alcohol consumption on cancer risk. Although we were able to consider cancer mortality as an outcome, we are not fully able to distinguish between death “from cancer” versus death “with cancer”. Although we restricted cases to those with a cancer outcome listed as a primary cause of death, it would require a brave doctor to sign a death certificate for a cancer patient who died from another cause and not list cancer as a cause of death. Hence, it is possible that alcohol consumption increases mortality in cancer patients, rather than increasing risk of mortality from cancer.

Finally, there are limitations around the populations under study. Epidemiological studies typically recruit participants that are healthier than average members of the underlying population, and so heavy drinkers may be underrepresented in our analyses. An exception is the Million Veteran Program, which recruited US military veterans, and contains a substantial proportion of heavy alcohol drinkers and ex-drinkers [86]. It is possible that the positive genetic association with liver cancer observed in this dataset reflects a harmful effect of heavy alcohol drinking that is not seen in datasets with low proportions of heavy drinkers.

Some aspects of our analyses were conducted in overlapping populations. In particular, the dataset used for the selection of variants overlapped with UK Biobank and possibly with some FinnGen participants. However, this overlap would typically bias estimates away from the null [87], and the overlap was only partial, meaning that bias should not be substantial.

Our analyses were primarily conducted in European ancestry populations. While we were able to consider other population groups in the All of US study, the mean age for European ancestry participants in All of US was 5 years greater than that for African ancestry participants and 10 years greater than that for admixed American ancestry participants, leading to lower numbers of cases and less statistical power in these population groups. Similarly, we could consider non-European ancestry participants in Million Veteran Program, but only for a subset of outcomes. Additionally, the genetic variants that we considered were selected based on European ancestry individuals and may not be strong predictors of alcohol consumption in non-European populations. However, we would not expect the physiological effects of alcohol consumption to differ strongly between humans of different ancestries, and previous analyses in Chinese populations have shown similar results to what we saw here.

## Conclusions

Our genetic epidemiological analysis provides moderate-to-weak evidence supporting causal effects of alcohol consumption on the risk of most “alcohol-related cancers”, including head/neck, oesophageal, colorectal, and liver cancers. No evidence was found to support an effect on breast cancer risk. Overall, human genetic data do not provide evidence that alcohol consumption is a cause of all cancers and suggest there may even be inverse associations with certain cancer types.

## Supplementary Information

Additional file 1: Table S1-S13. Table S1. Consortium datasets used in analyses. Table S2. Codes used in defining cancer outcomes in UK Biobank and All of US. Table S3. Codes used in defining cancer outcomes in FinnGen. Table S4. Codes used in defining cancer outcomes in Million Veteran Program. Table S5. List of 95 genetic variants used in analyses. Table S6. Proxy variants used in FinnGen. Table S7. Combined results from multivariable Mendelian randomization analysis adjusted for education calculated using outcome data from all four biobank datasets. Table S8. Results from multivariable Mendelian randomization analysis adjusted for body mass index calculated using outcome data from UK Biobank. Table S9. Results from multivariable Mendelian randomization analysis adjusted for education calculated using outcome data from consortium datasets. Table S10. Results from weighted median, MR-Egger, and contamination mixture methods and heterogeneity test statistics from the inverse-variance weighted method for European ancestry participants in UK Biobank. Table S11. Results from weighted median, MR-Egger, and contamination mixture methods and heterogeneity test statistics for FinnGen. Table S12. Results from weighted median, MR-Egger, and contamination mixture methods and heterogeneity test statistics for European ancestry participants in All of US. Table S13. Results from weighted median, MR-Egger, and contamination mixture methods and heterogeneity test statistics for European ancestry participants in Million Veteran Program. Table S14. Results from weighted median, MR-Egger, and contamination mixture methods and heterogeneity test statistics for consortium datasets. Table S15. Between-study heterogeneity in primary estimates: Q statistics and associated *p*-values.

Additional file 2: Figure S1-S31. Fig. S1. Mendelian randomization estimates calculated using outcome data on All of US African ancestry participants. Fig. S2. Mendelian randomization estimates calculated using outcome data on Million Veteran Program African ancestry participants. Fig. S3. Mendelian randomization estimates calculated using outcome data on All of US American admixed ancestry participants. Fig. S4. Mendelian randomization estimates calculated using outcome data on Million Veteran Program American admixed ancestry participants. Fig. S5. Mendelian randomization estimates calculated using outcome data on UK Biobank European ancestry participants. Fig. S6. Mendelian randomization estimates calculated using outcome data on FinnGen participants. Fig. S7. Mendelian randomization estimates calculated using outcome data on All of US European ancestry participants. Fig. S8. Mendelian randomization estimates calculated using outcome data on Million Veteran Program European ancestry participants. Fig. S9. Genetic associations with alcohol consumption and with risk of any breast cancer from consortium data. Fig. S10. Genetic associations with alcohol consumption and with risk of triple negative breast cancer from consortium data. Fig. S11. Genetic associations with alcohol consumption and with risk of triple negative or BRCA+ breast cancer from consortium data. Fig. S12. Genetic associations with alcohol consumption and with risk of luminal A breast cancer from consortium data. Fig. S13. Genetic associations with alcohol consumption and with risk of luminal B breast cancer from consortium data. Fig. S14. Genetic associations with alcohol consumption and with risk of luminal B or HER2- breast cancer from consortium data. Fig. S15. Genetic associations with alcohol consumption and with risk of HER2 enriched breast cancer from consortium data. Fig. S16. Genetic associations with alcohol consumption and with risk of breast cancer survival (Escala-Garcia 2019) from consortium data. Fig. S17. Genetic associations with alcohol consumption and with risk of breast cancer survival (Morra 2021) from consortium data. Fig. S18. Genetic associations with alcohol consumption and with risk of non-mucinous ovarian cancer from consortium data. Fig. S19. Genetic associations with alcohol consumption and with risk of mucinous ovarian cancer from consortium data. Fig. S20. Genetic associations with alcohol consumption and with risk of high grade serous ovarian cancer from consortium data. Fig. S21. Genetic associations with alcohol consumption and with risk of low grade serous ovarian cancer from consortium data. Fig. S22. Genetic associations with alcohol consumption and with risk of endometrioid ovarian cancer from consortium data. Fig. S23. Genetic associations with alcohol consumption and with risk of clear cell ovarian cancer from consortium data. Fig. S24. Genetic associations with alcohol consumption and with risk of endometrial cancer from consortium data. Fig. S25. Genetic associations with alcohol consumption and with risk of prostate cancer from consortium data. Fig. S26. Genetic associations with alcohol consumption and with risk of any kidney cancer from consortium data. Fig. S27. Genetic associations with alcohol consumption and with risk of clear renal cell carcinoma from consortium data. Fig. S28. Genetic associations with alcohol consumption and with risk of papillary renal cell carcinoma from consortium data. Fig. S29. Genetic associations with alcohol consumption and with risk of colorectum cancer from consortium data Fig. S30. Genetic associations with alcohol consumption and with risk of oesophagus cancer from consortium data. Fig. S31. Genetic associations with alcohol consumption and with risk of Barrett's oesophagus from consortium data.

Additional file 3. STROBE-MR checklist.

## Data Availability

Data used in the writing of this paper are publicly available. UK Biobank and All of US data are available on registration to any *bona fide* researcher. FinnGen data are available at https://finngen.gitbook.io/documentation/. Million Veteran Program data are available via the MVP accession phs002453.v1.p1 in the Database of Genotypes and Phenotypes (dbGaP). Consortium data are available through the GWAS catalogue, or as otherwise indicated. See Supplementary Table 1 for accession numbers.

## Abbreviations

ADH1B: Alcohol dehydrogenase 1B
CI: Confidence interval
GWAS: Genome-wide association study
ICD: International Classification of Diseases
MR-PRESSO: Mendelian Randomization Pleiotropy RESidual Sum and Outlier
OR: Odds ratio
