# Supplementary material for: Alcohol consumption and risk of cancer: a Mendelian randomization analysis of four biobanks and consortium data
Source: BMC Med. 2025 Dec 16;23:676. doi: 10.1186/s12916-025-04543-8 (PMC12707013; doi:10.1186/s12916-025-04543-8)
Supplement: Supplementary file 1 — Additional file 1: Table S1-S13. Table S1. Consortium datasets used in analyses. Table S2. Codes used in defining cancer outcomes in UK Biobank and All of US. Table S3. Codes used in defining cancer outcomes in FinnGen. Table S4. Codes used in defining cancer outcomes in Million Veteran Program. Table S5. List of 95 genetic variants used in analyses. Table S6. Proxy variants used in FinnGen. Table S7. Combined results from multivariable Mendelian randomization analysis adjusted for education calculated using outcome data from all four biobank datasets. Table S8. Results from multivariable Mendelian randomization analysis adjusted for body mass index calculated using outcome data from UK Biobank. Table S9. Results from multivariable Mendelian randomization analysis adjusted for education calculated using outcome data from consortium datasets. Table S10. Results from weighted median, MR-Egger, and contamination mixture methods and heterogeneity test statistics from the inverse-variance weighted method for European ancestry participants in UK Biobank. Table S11. Results from weighted median, MR-Egger, and contamination mixture methods and heterogeneity test statistics for FinnGen. Table S12. Results from weighted median, MR-Egger, and contamination mixture methods and heterogeneity test statistics for European ancestry participants in All of US. Table S13. Results from weighted median, MR-Egger, and contamination mixture methods and heterogeneity test statistics for European ancestry participants in Million Veteran Program. Table S14. Results from weighted median, MR-Egger, and contamination mixture methods and heterogeneity test statistics for consortium datasets. Table S15. Between-study heterogeneity in primary estimates: Q statistics and associated p-values. [file 12916_2025_4543_MOESM1_ESM.docx]

**Additional file 1**

**Table S1.** Consortium datasets used in analyses.

**Table S2.** Codes used in defining cancer outcomes in UK Biobank and All of US.

**Table S3.** Codes used in defining cancer outcomes in FinnGen.

**Table S4.** Codes used in defining cancer outcomes in Million Veteran Program.

**Table S5.** List of 95 genetic variants used in analyses.

**Table S6.** Proxy variants used in FinnGen.

**Table S7.** Combined results from multivariable Mendelian randomization analysis adjusted for education calculated using outcome data from all four biobank datasets.

**Table S8.** Results from multivariable Mendelian randomization analysis adjusted for body mass index calculated using outcome data from UK Biobank.

**Table S9.** Results from multivariable Mendelian randomization analysis adjusted for education calculated using outcome data from consortium datasets.

**Table S10.** Results from weighted median, MR-Egger, and contamination mixture methods and heterogeneity test statistics from the inverse-variance weighted method for European ancestry participants in UK Biobank.

**Table S11.** Results from weighted median, MR-Egger, and contamination mixture methods and heterogeneity test statistics for FinnGen.

**Table S12.** Results from weighted median, MR-Egger, and contamination mixture methods and heterogeneity test statistics for European ancestry participants in All of US.

**Table S13.** Results from weighted median, MR-Egger, and contamination mixture methods and heterogeneity test statistics for European ancestry participants in Million Veteran Program.

**Table S14.** Results from weighted median, MR-Egger, and contamination mixture methods and heterogeneity test statistics for consortium datasets.

**Table S15.** Between-study heterogeneity in primary estimates: Q statistics and associated p-values

**Table S1.** Consortium datasets used in analyses

| **Cancer type (and subtype) / site** | **GWAS Catalogue study accession** |
| --- | --- |
| Any breast | GCST010098 |
| Breast triple negative | GCST010100 |
| Breast triple negative or BRCA+ | GCST90454344 |
| Breast luminal A | GCST90454345 |
| Breast luminal B | GCST90454346 |
| Breast luminal B or HER2- | GCST90454347 |
| Breast HER2 enriched | GCST90454348 |
| Breast survival Escala Garcia 2019 | [link](https://www.ccge.medschl.cam.ac.uk/breast-cancer-association-consortium-bcac/data-data-access/summary-results/gwas-summary-results-2) |
| Breast survival Morra 2021 | [link](https://www.ccge.medschl.cam.ac.uk/breast-cancer-association-consortium-bcac/data-data-access/summary-results/gwas-summary-results-0) |
| Ovarian non mucinous | GCST90244167 |
| Ovarian mucinous | GCST90244172 |
| Ovarian high grade serous | GCST90244168 |
| Ovarian low grade serous | GCST90244169 |
| Ovarian endometrioid | GCST90244170 |
| Ovarian clear cell | GCST90244171 |
| Endometrial | GCST006464 |
| Prostate | GCST90274714 |
| Any kidney | GCST90320057 |
| Clear renal cell carcinoma | GCST90320058 |
| Papillary renal cell carcinoma | GCST90320059 |
| Colorectum | GCST90255675 |
| Oesophagus | GCST003739 |
| Barrett’s oesophagus | GCST003738 |

**Table S2.** Codes used in defining cancer outcomes in UK Biobank and All of US

| **Cancer site/ cancer** | **ICD-9 codes** | **ICD-10 codes** | **Self report (field 20001)** | **Cancer histology (field 40011)** |
| --- | --- | --- | --- | --- |
| Bladder | 188.X, 189.1, 189.2, V10.51, V10.53 | C67.X, C65.X, C66.X, Z85.51, Z85.54, Z85.53 | 1035 |  |
| Brain | 191.X, 192.0, 192.1, 192.2, 192.3, V10.85 | C70.X, C71.X, C72.0, C72.3, Z85.841 | 1031, 1032, 1033 |  |
| Breast | 174.X, 175.X, V10.3 | C50.X, Z85.3 | 1002 |  |
| Cervix | 180.X, V10.41 | C53.X, Z85.41 | 1041 |  |
| Colorectum | 153.X, 154.0, 154.1, V10.05, V10.06 | C18.X, C19.X, C20.X, Z85.038, Z85.048 | 1020, 1022, 1023 |  |
| Head/neck | 140.X, 141.X, 142.X, 143.X, 144.X, 145.X, 146.X, 147.X, 148.X, 149.X, 160.X, 161.X, V10.01, V10.02, V10.21, V10.22 | C00.X, C01.X, C02.X, C03.X, C04.X, C05.X, C06.X, C07.X, C08.X, C09.X, C10.X, C11.X, C12.X, C13.X, C14.X, C30.X, C31.X, C32.X, Z85.21, Z85.22, Z85.81 | 1006, 1007, 1009, 1004, 1010, 1011, 1012, 1077, 1078, 1079, 1005, 1015, 1016 |  |
| Kidney | 189.0, V10.52 | C64.X, Z85.528 | 1034 |  |
| Leukaemia | 204.X, 205.X, 206.X, 207.X, 208.X, V10.6 | C91.X, C92.X, C93.X, C94.0, C94.2, C94.3, C94.4, C94.8, C95, Z85.6 | 1048, 1055, 1056, 1074 |  |
| Liver | 155.0 | C22.0 | 1024 | 8170, 8171, 8172, 8173, 8174, 8175 |
| Lung | 162.X, V10.1 | C33.X, C34.X, C39.9, Z85.1 | 1001, 1027, 1028, 1080 |  |
| Melanoma | 172.X, V10.82 | C43.X, Z85.820 | 1059 |  |
| Myeloma | 203.0, 203.1 | C90.0, C90.1 | 1050 | 9732, 9733 |
| Non-Hodgkin’s Lymphoma | 200.X, 202.0, 202.1, 202.2, 202.7, V10.71 | C82.X, C83.X, C84.X, C85.X, C86.X, C88.0, C88.4, Z85.72 | 1053 |  |
| Oesophagus | 150.X, V10.03 | C15.X, Z85.01 | 1017 |  |
| Ovaries | 183.0, 183.2, 183.8, 183.9, V10.43 | C56.X, C57.0, C57.4, Z85.43 | 1039 |  |
| Pancreas | 157.X | C25.X, Z85.07 | 1034 |  |
| Prostate | 185.X, V10.46 | C61.X, Z85.46 | 1044 |  |
| Stomach | 151.X, V10.04 | C16.X, Z85.028 | 1018 |  |
| Testes | 186.X, V10.47 | C62.X, Z85.47 | 1045 |  |
| Thyroid | 193.X, V10.87 | C73.X, Z85.850 | 1065 |  |
| Uterus | 179.X, 182.X, V10.42 | C54.X, C55.X, Z85.42 | 1040 |  |

**Table S3.** Codes used in defining cancer outcomes in FinnGen

| Cancer site/cancer | ICD-9 codes | ICD-10 codes | Cancer registry: Topography ICD-O-3 | Phenocode |
| --- | --- | --- | --- | --- |
| Bladder | 188 | C67 | C67 | C3_BLADDER_EXALLC |
| Brain | 191 | C71 | C71 | C3_BRAIN_EXALLC |
| Breast | 174 | C50 | C50 | C3_BREAST_EXALLC |
| Cervix | 180 | C53 | C53 | C3_CERVIX_UTERI_EXALLC |
| Colorectum | 153, 1540, 154 | C18, C19, C20 | C18, C19, C20 | C3_COLORECTAL_EXALLC |
| Head/neck | 14[0-9], 161 | C0[1-8], C1[1-4], C32 | C0[1-8], C1[1-4], C32 | C3_HEAD_AND_NECK_EXALLC |
| Kidney | 1890 | C64 | C64 | C3_KIDNEY_NOTRENALPELVIS_EXALLC |
| Leukaemia | 204 | C91 |  | CD2_LYMPHOID_LEUKAEMIA_EXALLC |
| Liver |  | C220 | C22 | C3_HEPATOCELLU_CARC_EXALLC |
| Lung |  | C34 | C34 | C3_LUNG_NONSMALL_EXALLC |
| Melanoma | 172 | C43 | C44 | C3_MELANOMA_SKIN_EXALLC |
| Myeloma | 2030A | C90 | ANY | C3_MULT_MYELOMA_EXALLC |
| Non-Hodgkin’s Lymphoma | 200 | C8[2-5] | ANY | C3_NONHODGKIN_EXALLC |
| Oesophagus | 150 | C15 | C15 | C3_OESOPHAGUS_EXALLC |
| Ovaries | 183 | C56 | C56 | C3_OVARY_EXALLC |
| Pancreas | 157 | C25 | C25 | C3_PANCREAS_EXALLC |
| Prostate | 185 | C61 | C61 | C3_PROSTATE_EXALLC |
| Stomach | 151 | C16 | C16 | C3_STOMACH_EXALLC |
| Testes | 186 | C62 | C62 | C3_TESTIS_EXALLC |
| Uterus | 182 | C54 | C54 | C3_CORPUS_UTERI_EXALLC |

**Table S4.** Codes used in defining cancer outcomes in Million Veteran Program

| Cancer site/cancer | ICD-10 codes | Phenocode |
| --- | --- | --- |
| Bladder | C67, Z85.51 | Phe_189_21 |
| Brain | C71, Z85.841 | Phe_191_11 |
| Breast | C50, D05, D48.6, D49.3, Z85.3 | Phe_174 |
| Colorectum | C18, C19, C20, C21[0,1,2,8],  C26.0, C7A.02, D01, R85.61, Z85.0[3,4] | Phe_153 |
| Head/neck | C76 | Phe_195_3 |
| Kidney | C64, Z85.52 | Phe_189_11 |
| Leukaemia | C88.2,3,9; C90, C91-95, D46, Z85.6 | Phe_204 |
| Liver | C22, Z85.05 | Phe_155_1 |
| Lung | C33, C34, C7A.090, D02.2, Z85.[1,2] | Phe_165_1 |
| Melanoma | C43, D03 | Phe_172_11 |
| Myeloma | C88.2,3,9; C90.0, C90.2, C90.3 | Phe_204_4 |
| Non-Hodgkin’s lymphoma | C82, C83, C84, C85, C86, C88.4, C91.A, C96.[4,9,Z],Z85.72 | Phe_202_2 |
| Oesophagus | C15, D00.1, Z85.01 | Phe_150 |
| Pancreas | C25, Z85.07 | Phe_157 |
| Prostate | C61, D07.5, Z85.46 | Phe_185 |
| Stomach | C16, C7A.0, C7A.092, D00.2, Z85.02 | Phe_151 |
| Testes | C60, C62, C63, D07, D40.[0,1,8,9], Z85.[45, 47, 48, 49] | Phe_187 |

**Table S5.** List of 95 genetic variants used in analyses

| rsid | Chromosome and position (hg19) | Effect allele | Other allele | Beta | SE |
| --- | --- | --- | --- | --- | --- |
| rs705687 | 01:4548453 | G | A | -0.011 | 0.002 |
| rs58107686 | 01:33837334 | A | C | -0.010 | 0.002 |
| rs12088813 | 01:66407700 | C | A | -0.009 | 0.002 |
| rs5024204 | 01:71491890 | T | A | 0.010 | 0.002 |
| rs10753661 | 01:165119792 | A | G | -0.009 | 0.002 |
| rs28680958 | 01:173848808 | A | G | -0.011 | 0.002 |
| rs823114 | 01:205719532 | A | G | 0.009 | 0.001 |
| rs77165542 | 02:430975 | T | C | -0.026 | 0.004 |
| rs1260326 | 02:27730940 | C | T | 0.021 | 0.001 |
| rs2178197 | 02:27860551 | G | A | -0.009 | 0.001 |
| rs13383034 | 02:45155276 | T | C | 0.015 | 0.002 |
| rs13032049 | 02:63581507 | G | A | 0.010 | 0.002 |
| rs828867 | 02:74334462 | A | G | 0.009 | 0.001 |
| rs11692435 | 02:98275354 | A | G | 0.017 | 0.003 |
| rs13024996 | 02:144225215 | A | C | -0.011 | 0.002 |
| rs72859280 | 02:147956293 | T | G | 0.023 | 0.004 |
| rs56337305 | 02:225475560 | C | T | -0.010 | 0.001 |
| rs13094887 | 03:70968431 | T | A | -0.010 | 0.002 |
| rs62250685 | 03:85457240 | G | A | -0.014 | 0.002 |
| rs13066454 | 03:93994255 | T | C | -0.009 | 0.001 |
| rs9838144 | 03:131576287 | C | G | -0.010 | 0.002 |
| rs2011092 | 03:141124607 | C | T | -0.009 | 0.002 |
| rs60654199 | 03:141267295 | A | C | -0.017 | 0.003 |
| rs6787172 | 03:158187811 | G | T | -0.008 | 0.001 |
| rs3748034 | 04:3446091 | T | G | -0.012 | 0.002 |
| rs7682824 | 04:39406254 | T | C | 0.008 | 0.002 |
| rs11940694 | 04:39414993 | G | A | 0.026 | 0.001 |
| rs4501255 | 04:42151306 | G | C | 0.011 | 0.002 |
| rs12499107 | 04:99678691 | G | A | 0.013 | 0.002 |
| rs144198753 | 04:99713350 | T | C | -0.042 | 0.006 |
| rs1154414 | 04:100000136 | C | T | 0.018 | 0.002 |
| rs1229984 | 04:100239319 | C | T | 0.151 | 0.004 |
| rs10028756 | 04:100254520 | A | G | -0.019 | 0.002 |
| rs561222871 | 04:100260679 | T | C | -0.039 | 0.004 |
| rs36052336 | 04:100273594 | G | A | -0.018 | 0.003 |
| rs2165670 | 04:100286085 | A | G | 0.023 | 0.002 |
| rs17029090 | 04:100443853 | G | A | -0.049 | 0.005 |
| rs4699791 | 04:101243023 | A | G | 0.019 | 0.002 |
| rs13107325 | 04:103188709 | T | C | -0.028 | 0.003 |
| rs4690727 | 04:143648579 | G | C | 0.011 | 0.002 |
| rs10004020 | 04:152968372 | A | G | 0.009 | 0.002 |
| rs12651313 | 04:171086393 | G | C | -0.009 | 0.001 |
| rs4916723 | 05:87854395 | C | A | -0.010 | 0.001 |
| rs12655091 | 05:144412335 | A | G | -0.008 | 0.001 |
| rs55872084 | 05:155902003 | T | G | 0.010 | 0.002 |
| rs11739827 | 05:166803321 | T | G | -0.008 | 0.001 |
| rs10085696 | 07:69783020 | G | A | -0.011 | 0.002 |
| rs6460047 | 07:73042443 | C | T | 0.012 | 0.002 |
| rs10236149 | 07:98977515 | G | A | -0.013 | 0.002 |
| rs35034355 | 07:103840115 | A | G | -0.008 | 0.001 |
| rs6951574 | 07:153489744 | C | T | 0.013 | 0.001 |
| rs13250583 | 08:20949917 | T | C | -0.010 | 0.002 |
| rs1217091 | 08:64527399 | C | T | 0.012 | 0.002 |
| rs28601761 | 08:126500031 | G | C | 0.009 | 0.001 |
| rs55932213 | 09:108755622 | G | A | 0.009 | 0.002 |
| rs10978550 | 09:109345993 | C | T | -0.012 | 0.002 |
| rs7074871 | 10:110507806 | A | G | -0.009 | 0.002 |
| rs17665139 | 10:125093880 | T | C | -0.012 | 0.002 |
| rs7950166 | 11:8642218 | T | C | -0.010 | 0.002 |
| rs11030084 | 11:27643725 | T | C | -0.011 | 0.002 |
| rs56030824 | 11:47397353 | A | G | -0.012 | 0.002 |
| rs10750025 | 11:113424042 | T | C | 0.010 | 0.002 |
| rs1713676 | 11:113660576 | G | A | -0.008 | 0.001 |
| rs4938230 | 11:116075001 | A | C | 0.013 | 0.002 |
| rs682011 | 11:121544285 | C | T | 0.008 | 0.001 |
| rs12795042 | 11:133658168 | C | A | -0.008 | 0.002 |
| rs10876188 | 12:51895882 | T | C | -0.008 | 0.001 |
| rs3809162 | 12:54674235 | G | A | 0.009 | 0.001 |
| rs10506274 | 12:81601464 | T | G | -0.009 | 0.001 |
| rs4842786 | 12:92170791 | A | G | -0.009 | 0.001 |
| rs500321 | 13:27124360 | T | A | -0.010 | 0.002 |
| rs1123285 | 14:57274519 | G | C | -0.009 | 0.002 |
| rs2180870 | 14:58782779 | C | T | -0.012 | 0.002 |
| rs28929474 | 14:94844947 | T | C | -0.037 | 0.005 |
| rs11625650 | 14:104610138 | A | G | -0.010 | 0.002 |
| rs2472297 | 15:75027880 | T | C | 0.011 | 0.002 |
| rs12907323 | 15:86796012 | G | A | 0.008 | 0.001 |
| rs2764771 | 16:20013793 | A | G | 0.010 | 0.002 |
| rs17177078 | 16:24810681 | T | C | -0.022 | 0.003 |
| rs378421 | 16:28754684 | A | G | -0.011 | 0.001 |
| rs113443718 | 16:29892184 | A | G | -0.010 | 0.002 |
| rs62044525 | 16:64872590 | G | C | -0.012 | 0.002 |
| rs7185555 | 16:69131281 | C | G | -0.011 | 0.002 |
| rs79616692 | 16:72338507 | C | G | 0.016 | 0.002 |
| rs1104608 | 16:73912588 | C | G | -0.011 | 0.001 |
| rs4548913 | 17:2209888 | A | G | -0.008 | 0.002 |
| rs3803800 | 17:7462969 | G | A | 0.011 | 0.002 |
| rs2854334 | 17:29715500 | G | A | 0.009 | 0.001 |
| rs2532276 | 17:44246624 | A | C | -0.022 | 0.003 |
| rs10438820 | 17:78524597 | T | C | 0.009 | 0.002 |
| rs9950000 | 18:53052169 | T | C | -0.009 | 0.001 |
| rs4092465 | 18:55080437 | G | A | -0.008 | 0.002 |
| rs281379 | 19:49214274 | A | G | 0.014 | 0.001 |
| rs4815364 | 20:25035711 | A | G | 0.009 | 0.001 |
| rs9607814 | 22:41946519 | A | C | -0.010 | 0.002 |

**Table S6.** Proxy variants used in FinnGen

| Original | Proxy | chr | pos | alleles | MAF | distance (bp) | R^2^ | Matching |
| --- | --- | --- | --- | --- | --- | --- | --- | --- |
| rs4501255 | rs6856819 | 4 | 42150213 | (C/G) | 0.2266 | 1093 | 0.9944 | C=C, G=G |
| rs561222871 | rs147851155 | 4 | 100282262 | (G/A) | 0.0795 | 21583 | 1 | C=G, T=A |
| rs6951574 | rs2622225 | 7 | 153492123 | (A/G) | 0.4612 | 2379 | 0.9454 | T=A, C=G |

Variants rs378421 and rs7682824 did not have suitable proxies, and so were not included in analyses.

**Table S7.** Combined results from multivariable Mendelian randomization analysis adjusted for education calculated using outcome data from all four biobank datasets

|  | **Unadjusted** | | |  | **Adjusted for education** | | |
| --- | --- | --- | --- | --- | --- | --- | --- |
| **Cancer type/site** | **Estimate** | **95% CI** | **p-value** |  | **Estimate** | **95% CI** | **p-value** |
| All cancer | 0.96 | 0.87, 1.06 | 0.45 |  | 0.95 | 0.86, 1.06 | 0.36 |
| All gastrointestinal | 1.17 | 0.92, 1.48 | 0.21 |  | 1.16 | 0.91, 1.50 | 0.23 |
| Bladder | 0.91 | 0.74, 1.12 | 0.36 |  | 0.88 | 0.71, 1.09 | 0.23 |
| Brain | 0.88 | 0.60, 1.28 | 0.49 |  | 0.86 | 0.58, 1.29 | 0.47 |
| Breast | 1.09 | 0.94, 1.25 | 0.25 |  | 1.03 | 0.90, 1.19 | 0.64 |
| Cervix | 1.53 | 0.98, 2.40 | 0.06 |  | 1.52 | 0.95, 2.42 | 0.08 |
| Colorectum | 1.21 | 1.01, 1.45 | 0.035 |  | 1.16 | 0.97, 1.40 | 0.11 |
| Head/neck | 1.51 | 1.18, 1.93 | 0.001 |  | 1.43 | 1.10, 1.85 | 0.007 |
| Kidney | 0.64 | 0.50, 0.82 | <0.001 |  | 0.66 | 0.51, 0.85 | 0.001 |
| Leukaemia | 0.83 | 0.65, 1.07 | 0.15 |  | 0.81 | 0.62, 1.05 | 0.12 |
| Liver | 1.40 | 0.93, 2.11 | 0.10 |  | 1.33 | 0.87, 2.03 | 0.19 |
| Lung | 1.41 | 1.17, 1.70 | <0.001 |  | 1.39 | 1.14, 1.70 | 0.001 |
| Melanoma | 0.91 | 0.76, 1.09 | 0.29 |  | 0.86 | 0.72, 1.04 | 0.12 |
| Myeloma | 0.61 | 0.41, 0.90 | 0.014 |  | 0.62 | 0.41, 0.94 | 0.024 |
| Non-Hodgkin's lymphoma | 0.75 | 0.61, 0.94 | 0.010 |  | 0.77 | 0.62, 0.97 | 0.025 |
| Oesophagus | 1.50 | 1.00, 2.25 | 0.049 |  | 1.48 | 0.97, 2.25 | 0.07 |
| Ovaries | 1.23 | 0.83, 1.82 | 0.30 |  | 1.16 | 0.77, 1.74 | 0.49 |
| Pancreas | 0.90 | 0.87, 1.06 | 0.45 |  | 0.88 | 0.62, 1.26 | 0.50 |
| Prostate | 0.92 | 0.92, 1.48 | 0.21 |  | 0.92 | 0.78, 1.09 | 0.34 |
| Stomach | 0.78 | 0.74, 1.12 | 0.36 |  | 0.73 | 0.48, 1.10 | 0.13 |
| Testes | 1.29 | 0.60, 1.28 | 0.49 |  | 1.23 | 0.78, 1.92 | 0.38 |
| Uterus | 1.00 | 0.94, 1.25 | 0.25 |  | 0.92 | 0.65, 1.31 | 0.63 |

Unadjusted estimates are provided for comparison. Estimates represent odds per 1 standard deviation increase in log-transformed number of drinks per week.

**Table S8.** Results from multivariable Mendelian randomization analysis adjusted for body mass index calculated using outcome data from UK Biobank

|  | **Unadjusted** | | |  | **Adjusted for BMI** | | |
| --- | --- | --- | --- | --- | --- | --- | --- |
| **Cancer site** | **Estimate** | **95% CI** | **p-value** |  | **Estimate** | **95% CI** | **p-value** |
| Kidney | 0.89 | 0.50, 1.60 | 0.70 |  | 0.77 | 0.43, 1.38 | 0.38 |
| Uterus | 0.71 | 0.39, 1.30 | 0.27 |  | 0.75 | 0.42, 1.36 | 0.35 |

Unadjusted estimates are provided for comparison. Estimates represent odds ratios per 1 standard deviation increase in log-transformed number of drinks per week.

**Table S9.** Results from multivariable Mendelian randomization analysis adjusted for education for consortium dataset

|  | **Unadjusted** | | |  | **Adjusted for education** | | |
| --- | --- | --- | --- | --- | --- | --- | --- |
| **Cancer type/site** | **Estimate** | **95% CI** | **p-value** |  | **Estimate** | **95% CI** | **p-value** |
| Any breast | 0.98 | 0.82, 1.17 | 0.84 |  | 0.94 | 0.79, 1.13 | 0.52 |
| Breast triple negative | 0.91 | 0.67, 1.22 | 0.53 |  | 0.93 | 0.67, 1.28 | 0.66 |
| Breast triple negative or BRCA+ | 0.96 | 0.78, 1.20 | 0.74 |  | 1.00 | 0.79, 1.27 | 0.99 |
| Breast luminal A | 0.96 | 0.77, 1.18 | 0.69 |  | 0.94 | 0.75, 1.16 | 0.55 |
| Breast luminal B | 0.83 | 0.58, 1.18 | 0.29 |  | 0.81 | 0.57, 1.15 | 0.23 |
| Breast luminal B or HER2- | 0.98 | 0.69, 1.37 | 0.89 |  | 0.98 | 0.69, 1.39 | 0.93 |
| Breast HER2 enriched | 1.38 | 0.88, 2.17 | 0.17 |  | 1.42 | 0.89, 2.25 | 0.14 |
| Breast survival (Escala-Garcia 2019) | 1.09 | 0.82, 1.44 | 0.57 |  | 1.17 | 0.87, 1.58 | 0.30 |
| Breast survival (Morra 2021) | 1.12 | 0.84, 1.49 | 0.45 |  | 1.24 | 0.92, 1.68 | 0.16 |
| Ovarian non-mucinous | 0.75 | 0.60, 0.94 | 0.013 |  | 0.84 | 0.69, 1.02 | 0.08 |
| Ovarian mucinous | 0.84 | 0.50, 1.42 | 0.52 |  | 0.82 | 0.48, 1.41 | 0.47 |
| Ovarian high grade serous | 0.67 | 0.51, 0.89 | 0.006 |  | 0.78 | 0.61, 0.99 | 0.038 |
| Ovarian low grade serous | 1.24 | 0.70, 2.19 | 0.46 |  | 1.16 | 0.63, 2.11 | 0.64 |
| Ovarian endometrioid | 0.67 | 0.39, 1.16 | 0.15 |  | 0.74 | 0.42, 1.30 | 0.29 |
| Ovarian clear cell | 1.35 | 0.68, 2.66 | 0.39 |  | 1.51 | 0.75, 3.05 | 0.25 |
| Endometrial | 0.56 | 0.41, 0.78 | <0.001 |  | 0.59 | 0.41, 0.83 | 0.002 |
| Prostate | 0.99 | 0.80, 1.23 | 0.93 |  | 0.97 | 0.78, 1.22 | 0.83 |
| Any kidney | 0.81 | 0.63, 1.03 | 0.09 |  | 0.82 | 0.64, 1.06 | 0.13 |
| Clear renal cell carcinoma | 0.84 | 0.61, 1.15 | 0.27 |  | 0.87 | 0.63, 1.21 | 0.41 |
| Papillary renal cell carcinoma | 0.65 | 0.33, 1.27 | 0.20 |  | 0.63 | 0.32, 1.26 | 0.19 |
| Colorectum | 1.23 | 0.98, 1.53 | 0.07 |  | 1.19 | 0.95, 1.50 | 0.12 |
| Oesophagus | 1.29 | 0.75, 2.23 | 0.35 |  | 1.28 | 0.73, 2.23 | 0.39 |
| Barrett's oesophagus | 0.95 | 0.62, 1.46 | 0.82 |  | 0.99 | 0.63, 1.54 | 0.96 |

Unadjusted estimates are provided for comparison. Estimates represent odds ratios (hazard ratios for breast cancer survival) per 1 standard deviation increase in log-transformed number of drinks per week.

**Table S9.** Results from weighted median, MR-Egger, and contamination mixture methods and heterogeneity test statistics for European ancestry participants in UK Biobank

|  | Weighted median | | | MR-Egger | | | Contamination mixture | | | Heterogeneity test* | |
| --- | --- | --- | --- | --- | --- | --- | --- | --- | --- | --- | --- |
| Cancer type/site | Estimate | 95% CI | p-value | Estimate | 95% CI | p-value | Estimate | 95% CI | p-value | Q statistic | p-value |
| All cancer | 1.16 | 0.98, 1.37 | 0.08 | 1.07 | 0.84, 1.36 | 0.59 | 1.21 | 0.54, 0.65 | 0.17 | 155.0 | <0.001 |
| All gastrointestinal | 1.25 | 0.78, 2.01 | 0.35 | 1.32 | 0.75, 2.32 | 0.33 | 1.24 | 0.91, 1.83 | 0.14 | 132.3 | 0.012 |
| Bladder | 1.07 | 0.47, 2.43 | 0.87 | 1.08 | 0.45, 2.61 | 0.86 | 0.91 | 0.50, 1.66 | 0.69 | 108.8 | 0.21 |
| Brain | 1.98 | 0.52, 7.59 | 0.32 | 1.37 | 0.22, 8.40 | 0.73 | 2.38 | 0.75, 6.81 | 0.11 | 137.3 | 0.005 |
| Breast | 1.30 | 0.89, 1.91 | 0.17 | 1.56 | 0.98, 2.49 | 0.06 | 1.25 | 0.87, 1.64 | 0.26 | 123.8 | 0.040 |
| Cervix | 1.27 | 0.42, 3.84 | 0.67 | 1.03 | 0.32, 3.28 | 0.96 | 1.17 | 0.49, 2.47 | 0.88 | 88.7 | 0.74 |
| Colorectum | 1.20 | 0.69, 2.07 | 0.52 | 1.37 | 0.68, 2.74 | 0.38 | 1.36 | 0.89, 1.91 | 0.16 | 136.1 | 0.007 |
| Head/neck | 2.16 | 0.88, 5.28 | 0.09 | 3.49 | 1.08, 11.32 | 0.037 | 2.57 | 1.13, 7.12 | 0.037 | 98.8 | 0.46 |
| Kidney | 0.59 | 0.20, 1.70 | 0.33 | 0.58 | 0.19, 1.81 | 0.35 | 1.02 | 0.46, 2.17 | 0.96 | 103.5 | 0.33 |
| Leukaemia | 0.88 | 0.31, 2.51 | 0.81 | 1.55 | 0.51, 4.75 | 0.44 | 0.52 | 0.18, 1.73 | 0.41 | 104.1 | 0.32 |
| Liver | 0.88 | 0.10, 7.52 | 0.91 | 0.75 | 0.08, 7.40 | 0.80 | 1.67 | 0.49, 5.50 | 0.42 | 108.3 | 0.22 |
| Lung | 1.36 | 0.66, 2.82 | 0.40 | 1.19 | 0.48, 2.91 | 0.71 | 2.07 | 1.16, 3.38 | 0.019 | 139.5 | 0.004 |
| Melanoma | 0.76 | 0.40, 1.45 | 0.41 | 0.61 | 0.30, 1.24 | 0.17 | 0.75 | 0.49, 1.16 | 0.15 | 116.2 | 0.10 |
| Myeloma | 0.91 | 0.22, 3.81 | 0.90 | 0.59 | 0.13, 2.75 | 0.50 | 0.75 | 0.28, 2.57 | 0.61 | 89.4 | 0.72 |
| Non-Hodgkin's lymphoma | 1.42 | 0.65, 3.11 | 0.38 | 1.59 | 0.61, 4.15 | 0.34 | 1.06 | 0.16, 2.04 | 0.82 | 112.6 | 0.15 |
| Oesophagus | 1.07 | 0.29, 3.95 | 0.91 | 1.04 | 0.24, 4.54 | 0.95 | 1.57 | 0.53, 3.60 | 0.48 | 109.7 | 0.20 |
| Ovaries | 0.54 | 0.20, 1.44 | 0.22 | 0.64 | 0.20, 2.07 | 0.45 | 0.78 | 0.34, 1.83 | 0.65 | 111.6 | 0.16 |
| Pancreas | 1.49 | 0.44, 5.08 | 0.53 | 1.96 | 0.51, 7.46 | 0.32 | 1.58 | 0.65, 3.59 | 0.26 | 69.0 | 0.99 |
| Prostate | 1.12 | 0.70, 1.80 | 0.63 | 1.28 | 0.71, 2.32 | 0.42 | 0.95 | 0.59, 1.56 | 0.72 | 147.8 | <0.001 |
| Stomach | 0.62 | 0.14, 2.74 | 0.53 | 0.59 | 0.12, 2.84 | 0.51 | 0.60 | 0.25, 1.50 | 0.29 | 109.7 | 0.20 |
| Testes | 2.40 | 0.46, 12.51 | 0.30 | 3.71 | 0.50, 27.26 | 0.20 | 1.46 | 0.18, 15.30 | 0.54 | 100.7 | 0.41 |
| Uterus | 0.79 | 0.29, 2.16 | 0.65 | 1.28 | 0.40, 4.08 | 0.67 | 0.95 | 0.33, 1.74 | 0.85 | 128.0 | 0.023 |
| Cancer mortality | 1.66 | 1.11, 2.50 | 0.014 | 1.79 | 1.10, 2.89 | 0.018 | 1.75 | 1.31, 2.36 | <0.001 | 123.9 | 0.040 |

Estimates represent odds ratios per 1 standard deviation increase in log-transformed number of drinks per week. *Heterogeneity test statistics from the inverse-variance weighted method.

**Table S10.** Results from weighted median, MR-Egger, and contamination mixture methods and heterogeneity test statistics for FinnGen

|  | Weighted median | | | MR-Egger | | | Contamination mixture | | | Heterogeneity test* | |
| --- | --- | --- | --- | --- | --- | --- | --- | --- | --- | --- | --- |
| Cancer type/site | Estimate | 95% CI | p-value | Estimate | 95% CI | p-value | Estimate | 95% CI | p-value | Q statistic | p-value |
| Bladder | 1.14 | 0.58, 2.22 | 0.70 | 1.11 | 0.90, 1.37 | 0.34 | 0.87 | 0.40, 5.01 | 0.79 | 119.7 | 0.05 |
| Brain | 1.88 | 0.69, 5.11 | 0.22 | 0.79 | 0.59, 1.06 | 0.12 | 2.62 | 0.86, 6.26 | 0.11 | 91.3 | 0.62 |
| Breast | 1.08 | 0.77, 1.50 | 0.66 | 1.01 | 0.99, 1.03 | 0.27 | 1.73 | 1.19, 2.28 | 0.005 | 131.0 | 0.010 |
| Cervix | 1.41 | 0.31, 6.41 | 0.66 | 1.12 | 0.69, 1.82 | 0.65 | 0.94 | 0.18, 5.01 | 0.91 | 130.0 | 0.012 |
| Colorectum | 0.95 | 0.61, 1.49 | 0.83 | 1.03 | 0.91, 1.15 | 0.66 | 0.75 | 0.46, 1.14 | 0.17 | 156.5 | <0.001 |
| Head/neck | 1.91 | 0.95, 3.83 | 0.07 | 0.85 | 0.66, 1.10 | 0.23 | 1.92 | 0.87, 4.45 | 0.11 | 100.0 | 0.37 |
| Kidney | 0.62 | 0.30, 1.27 | 0.19 | 0.90 | 0.75, 1.08 | 0.27 | 0.49 | 0.20, 1.19 | 0.08 | 108.6 | 0.18 |
| Leukaemia | 0.94 | 0.34, 2.61 | 0.91 | 1.05 | 0.86, 1.29 | 0.64 | 1.43 | 0.28, 4.25 | 0.55 | 119.7 | 0.05 |
| Liver | 0.74 | 0.17, 3.25 | 0.69 | 0.92 | 0.70, 1.19 | 0.51 | 0.61 | 0.14, 2.87 | 0.40 | 109.3 | 0.17 |
| Lung | 1.61 | 0.90, 2.86 | 0.11 | 1.00 | 0.95, 1.06 | 0.95 | 3.07 | 1.60, 7.05 | 0.007 | 112.2 | 0.12 |
| Melanoma | 0.83 | 0.46, 1.49 | 0.53 | 0.97 | 0.86, 1.09 | 0.62 | 0.82 | 0.45, 1.58 | 0.58 | 111.0 | 0.14 |
| Myeloma | 0.35 | 0.11, 1.09 | 0.07 | 1.32 | 0.97, 1.78 | 0.07 | 0.53 | 0.15, 2.05 | 0.31 | 117.8 | 0.07 |
| Non-Hodgkin's | 0.53 | 0.29, 0.98 | 0.042 | 0.94 | 0.77, 1.16 | 0.58 | 0.46 | 0.23, 0.87 | 0.019 | 90.0 | 0.65 |
| Oesophagus | 2.09 | 0.61, 7.21 | 0.24 | 1.09 | 0.85, 1.40 | 0.49 | 5.20 | 1.06, 24.27 | 0.043 | 92.1 | 0.59 |
| Ovaries | 0.94 | 0.35, 2.57 | 0.91 | 1.05 | 0.92, 1.21 | 0.45 | 0.62 | 0.04, 3.04 | 0.42 | 152.4 | <0.001 |
| Pancreas | 0.77 | 0.33, 1.77 | 0.53 | 1.09 | 1.00, 1.19 | 0.06 | 1.12 | 0.38, 4.42 | 0.83 | 126.0 | 0.022 |
| Prostate | 0.88 | 0.59, 1.32 | 0.54 | 1.04 | 0.98, 1.11 | 0.19 | 0.78 | 0.51, 1.35 | 0.36 | 200.8 | <0.001 |
| Stomach | 0.92 | 0.37, 2.28 | 0.85 | 0.98 | 0.90, 1.06 | 0.58 | 1.24 | 0.54, 4.16 | 0.50 | 96.1 | 0.48 |
| Testes | 2.95 | 0.55, 15.83 | 0.21 | 1.24 | 0.72, 2.12 | 0.43 | 6.06 | 1.01, 31.25 | 0.049 | 99.8 | 0.38 |
| Uterus | 1.26 | 0.58, 2.73 | 0.55 | 0.99 | 0.86, 1.13 | 0.87 | 1.45 | 0.74, 2.78 | 0.30 | 110.7 | 0.15 |

Estimates represent odds ratios per 1 standard deviation increase in log-transformed number of drinks per week. *Heterogeneity test statistics from the inverse-variance weighted method.

**Table S11.** Results from weighted median, MR-Egger, and contamination mixture methods and heterogeneity test statistics for European ancestry participants in All of US

|  | Weighted median | | | MR-Egger | | | Contamination mixture | | | Heterogeneity test* | |  |
| --- | --- | --- | --- | --- | --- | --- | --- | --- | --- | --- | --- | --- |
| Cancer type/site | Estimate | 95% CI | p-value | Estimate | 95% CI | p-value | Estimate | 95% CI | p-value | Q statistic | p-value | |
| Bladder | 0.69 | 0.28, 1.70 | 0.42 | 0.78 | 0.60, 1.02 | 0.07 | 0.83 | 0.37, 1.69 | 0.53 | 135.6 | 0.006 | |
| Brain | 0.44 | 0.13, 1.51 | 0.19 | 0.86 | 0.42, 1.79 | 0.69 | 0.55 | 0.21, 1.74 | 0.24 | 131.8 | 0.011 | |
| Breast | 0.86 | 0.54, 1.38 | 0.54 | 0.60 | 0.23, 1.52 | 0.28 | 0.87 | 0.59, 1.28 | 0.39 | 109.2 | 0.19 | |
| Cervix | 2.16 | 0.46, 10.12 | 0.33 | 0.34 | 0.10, 1.15 | 0.08 | 1.50 | 0.48, 6.10 | 0.44 | 97.8 | 0.46 | |
| Colorectum | 0.69 | 0.31, 1.56 | 0.38 | 0.89 | 0.56, 1.41 | 0.61 | 1.04 | 0.51, 9.30 | 0.89 | 102.0 | 0.34 | |
| Head/neck | 0.67 | 0.21, 2.11 | 0.49 | 0.75 | 0.16, 3.52 | 0.71 | 0.67 | 0.32, 1.56 | 0.35 | 104.3 | 0.29 | |
| Kidney | 0.47 | 0.18, 1.26 | 0.14 | 0.73 | 0.29, 1.81 | 0.50 | 0.45 | 0.23, 0.85 | 0.017 | 133.4 | 0.008 | |
| Leukaemia | 0.59 | 0.22, 1.57 | 0.29 | 0.61 | 0.20, 1.85 | 0.38 | 0.84 | 0.38, 1.66 | 0.59 | 84.1 | 0.82 | |
| Liver | 0.52 | 0.08, 3.47 | 0.50 | 0.47 | 0.17, 1.27 | 0.14 | 0.63 | 0.06, 2.54 | 0.50 | 105.7 | 0.26 | |
| Lung | 0.48 | 0.22, 1.07 | 0.07 | 0.64 | 0.25, 1.69 | 0.37 | 2.38 | 0.46, 6.39 | 0.27 | 63.2 | 1.00 | |
| Melanoma | 0.60 | 0.32, 1.14 | 0.12 | 0.48 | 0.07, 3.23 | 0.45 | 0.63 | 0.39, 1.01 | 0.06 | 103.4 | 0.31 | |
| Myeloma | 0.24 | 0.07, 0.82 | 0.023 | 0.41 | 0.17, 0.97 | 0.043 | 0.14 | 0.06, 0.60 | 0.006 | 127.3 | 0.021 | |
| Non-Hodgkin's | 0.71 | 0.31, 1.59 | 0.40 | 0.62 | 0.29, 1.32 | 0.21 | 0.56 | 0.27, 1.16 | 0.14 | 146.0 | <0.001 | |
| Oesophagus | 3.97 | 0.43, 36.72 | 0.22 | 0.16 | 0.04, 0.67 | 0.013 | 2.72 | 0.63, 12.33 | 0.18 | 140.7 | 0.002 | |
| Ovaries | 1.19 | 0.36, 3.97 | 0.77 | 0.55 | 0.24, 1.23 | 0.15 | 2.23 | 0.76, 31.52 | 0.15 | 100.4 | 0.39 | |
| Pancreas | 1.74 | 0.38, 7.99 | 0.47 | 5.11 | 0.58, 44.70 | 0.14 | 1.90 | 0.47, 5.77 | 0.35 | 86.5 | 0.77 | |
| Prostate | 0.70 | 0.32, 1.53 | 0.37 | 1.21 | 0.37, 3.89 | 0.75 | 0.93 | 0.53, 4.23 | 0.81 | 78.4 | 0.92 | |
| Stomach | 0.61 | 0.09, 4.20 | 0.62 | 1.46 | 0.34, 6.33 | 0.61 | 0.62 | 0.15, 3.88 | 0.55 | 84.7 | 0.81 | |
| Testes | 4.99 | 0.39, 63.38 | 0.21 | 0.81 | 0.35, 1.86 | 0.62 | 2.62 | 0.39, 17.19 | 0.27 | 115.6 | 0.10 | |
| Uterus | 1.25 | 0.41, 3.83 | 0.70 | 0.61 | 0.09, 4.15 | 0.61 | 0.88 | 0.43, 1.94 | 0.79 | 101.0 | 0.37 | |

Estimates represent odds ratios per 1 standard deviation increase in log-transformed number of drinks per week. *Heterogeneity test statistics from the inverse-variance weighted method.**Table S12.** Results from weighted median, MR-Egger, and contamination mixture methods and heterogeneity test statistics for European ancestry participants in Million Veteran Program

|  | Weighted median | | | MR-Egger | | | Contamination mixture | | | Heterogeneity test* | |
| --- | --- | --- | --- | --- | --- | --- | --- | --- | --- | --- | --- |
| Cancer type/site | Estimate | 95% CI | p-value | Estimate | 95% CI | p-value | Estimate | 95% CI | p-value | Q-statistic | p-value |
| Bladder | 0.62 | 0.39, 0.99 | 0.047 | 0.54 | 0.32, 0.90 | 0.018 | 0.61 | 0.43, 0.86 | 0.007 | 126.3 | 0.024 |
| Brain | 0.96 | 0.23, 4.02 | 0.96 | 1.23 | 0.28, 5.36 | 0.78 | 0.42 | 0.09, 1.20 | 0.09 | 84.7 | 0.81 |
| Breast | 1.10 | 0.40, 3.01 | 0.85 | 1.50 | 0.52, 4.28 | 0.45 | 1.23 | 0.47, 2.66 | 0.86 | 107.3 | 0.22 |
| Colorectum | 2.30 | 1.51, 3.51 | <0.001 | 2.45 | 1.50, 3.98 | <0.001 | 1.74 | 1.07, 2.73 | 0.033 | 123.6 | 0.035 |
| Head/neck | 2.16 | 1.06, 4.42 | 0.034 | 1.68 | 0.83, 3.38 | 0.15 | 2.19 | 1.20, 3.75 | 0.010 | 98.1 | 0.45 |
| Kidney | 1.05 | 0.58, 1.90 | 0.88 | 1.03 | 0.53, 2.00 | 0.94 | 0.80 | 0.30, 1.24 | 0.27 | 115.5 | 0.10 |
| Leukaemia | 1.25 | 0.70, 2.24 | 0.46 | 1.09 | 0.59, 2.03 | 0.77 | 1.23 | 0.76, 1.91 | 0.41 | 112.5 | 0.14 |
| Liver | 3.22 | 1.34, 7.75 | 0.009 | 1.85 | 0.72, 4.71 | 0.20 | 3.52 | 1.38, 6.23 | 0.016 | 110.1 | 0.17 |
| Lung | 1.59 | 1.03, 2.46 | 0.036 | 1.50 | 0.93, 2.42 | 0.10 | 2.02 | 1.37, 4.03 | 0.002 | 128.4 | 0.018 |
| Melanoma | 0.97 | 0.57, 1.63 | 0.90 | 0.94 | 0.55, 1.60 | 0.82 | 1.11 | 0.74, 1.61 | 0.56 | 109.0 | 0.19 |
| Myeloma | 0.55 | 0.16, 1.97 | 0.36 | 0.64 | 0.17, 2.34 | 0.50 | 0.61 | 0.26, 2.04 | 0.31 | 100.1 | 0.39 |
| Non-Hodgkin's | 1.40 | 0.77, 2.56 | 0.27 | 1.27 | 0.67, 2.40 | 0.46 | 1.32 | 0.81, 2.38 | 0.28 | 116.9 | 0.08 |
| Oesophagus | 1.68 | 0.57, 4.99 | 0.35 | 0.92 | 0.27, 3.19 | 0.90 | 1.95 | 0.87, 4.60 | 0.10 | 133.8 | 0.008 |
| Pancreas | 1.14 | 0.36, 3.59 | 0.83 | 0.60 | 0.19, 1.88 | 0.38 | 1.01 | 0.48, 2.33 | 0.98 | 104.5 | 0.28 |
| Prostate | 1.27 | 0.96, 1.67 | 0.09 | 1.42 | 0.92, 2.20 | 0.12 | 1.26 | 1.01, 1.65 | 0.045 | 265.7 | <0.001 |
| Stomach | 0.82 | 0.18, 3.73 | 0.80 | 0.74 | 0.16, 3.36 | 0.70 | 0.59 | 0.20, 1.75 | 0.38 | 77.7 | 0.93 |
| Testes | 1.30 | 0.46, 3.62 | 0.62 | 1.39 | 0.50, 3.85 | 0.52 | 1.31 | 0.58, 2.69 | 0.49 | 89.9 | 0.68 |

Estimates represent odds ratios per 1 standard deviation increase in log-transformed number of drinks per week. *Heterogeneity test statistics from the inverse-variance weighted method.

**Table S13.** Results from weighted median, MR-Egger, and contamination mixture methods and heterogeneity test statistics for consortium datasets

|  | Weighted median | | | MR-Egger | | | Contamination mixture | | | Heterogeneity test* | |
| --- | --- | --- | --- | --- | --- | --- | --- | --- | --- | --- | --- |
| Cancer type/site | Estimate | 95% CI | p-value | Estimate | 95% CI | p-value | Estimate | 95% CI | p-value | Q-statistic | p-value |
| Any breast | 0.86 | 0.71, 1.04 | 0.13 | 0.96 | 0.70, 1.33 | 0.82 | 0.81 | 0.68, 1.70 | 0.30 | 283.6 | <0.001 |
| Breast triple negative | 0.81 | 0.51, 1.28 | 0.37 | 0.80 | 0.48, 1.34 | 0.41 | 0.74 | 0.53, 1.08 | 0.13 | 130.7 | 0.015 |
| Breast triple negative or BRCA+ | 1.02 | 0.73, 1.42 | 0.92 | 1.03 | 0.72, 1.47 | 0.88 | 0.98 | 0.76, 1.33 | 0.81 | 121.9 | 0.05 |
| Breast luminal A | 0.93 | 0.72, 1.21 | 0.59 | 1.00 | 0.69, 1.46 | 0.99 | 0.97 | 0.72, 1.16 | 0.69 | 230.5 | <0.001 |
| Breast luminal B | 0.51 | 0.30, 0.85 | 0.011 | 0.60 | 0.33, 1.09 | 0.09 | 0.50 | 0.35, 0.80 | 0.014 | 139.5 | 0.004 |
| Breast luminal B or HER2- | 0.89 | 0.56, 1.41 | 0.62 | 0.98 | 0.54, 1.77 | 0.95 | 0.99 | 0.68, 1.51 | 0.97 | 171.0 | <0.001 |
| Breast HER2 enriched | 1.68 | 0.76, 3.72 | 0.20 | 1.67 | 0.76, 3.67 | 0.20 | 2.08 | 0.98, 3.99 | 0.06 | 104.8 | 0.30 |
| Breast survival (Escala-Garcia 2019) | 1.02 | 0.61, 1.71 | 0.94 | 0.95 | 0.57, 1.56 | 0.83 | 1.31 | 0.92, 2.16 | 0.13 | 91.9 | 0.65 |
| Breast survival (Morra 2021) | 0.96 | 0.57, 1.62 | 0.89 | 0.91 | 0.55, 1.51 | 0.72 | 1.30 | 0.79, 2.97 | 0.26 | 84.9 | 0.82 |
| Ovarian non-mucinous | 0.63 | 0.45, 0.88 | 0.007 | 0.57 | 0.38, 0.85 | 0.006 | 0.65 | 0.51, 0.88 | 0.015 | 136.2 | 0.007 |
| Ovarian mucinous | 1.07 | 0.40, 2.84 | 0.90 | 1.24 | 0.47, 3.27 | 0.66 | 0.83 | 0.36, 1.78 | 0.68 | 90.9 | 0.68 |
| Ovarian high grade serous | 0.49 | 0.33, 0.72 | <0.001 | 0.44 | 0.27, 0.72 | 0.001 | 0.55 | 0.37, 1.90 | 0.06 | 156.0 | <0.001 |
| Ovarian low grade serous | 1.44 | 0.56, 3.71 | 0.45 | 1.35 | 0.47, 3.85 | 0.58 | 1.52 | 0.74, 3.09 | 0.22 | 128.6 | 0.021 |
| Ovarian endometrioid | 1.45 | 0.63, 3.32 | 0.38 | 1.30 | 0.48, 3.53 | 0.61 | 1.00 | 0.18, 1.84 | 1.00 | 125.4 | 0.032 |
| Ovarian clear cell | 0.51 | 0.16, 1.61 | 0.25 | 0.75 | 0.22, 2.58 | 0.65 | 0.90 | 0.29, 2.05 | 0.78 | 90.8 | 0.69 |
| Endometrial | 0.55 | 0.32, 0.93 | 0.026 | 0.80 | 0.44, 1.47 | 0.47 | 0.51 | 0.31, 0.74 | <0.001 | 136.7 | 0.006 |
| Prostate | 1.18 | 0.97, 1.44 | 0.09 | 1.31 | 0.88, 1.95 | 0.19 | 1.30 | 1.08, 1.56 | 0.004 | 459.9 | <0.001 |
| Any kidney | 1.08 | 0.77, 1.51 | 0.65 | 1.01 | 0.65, 1.57 | 0.96 | 0.90 | 0.51, 1.18 | 0.35 | 159.6 | <0.001 |
| Clear renal cell carcinoma | 1.28 | 0.85, 1.93 | 0.24 | 1.06 | 0.61, 1.83 | 0.85 | 0.96 | 0.53, 1.46 | 0.75 | 158.1 | <0.001 |
| Papillary renal cell carcinoma | 1.00 | 0.34, 2.93 | 1.00 | 0.86 | 0.27, 2.79 | 0.81 | 0.86 | 0.42, 1.71 | 0.69 | 120.0 | 0.049 |
| Colorectum | 1.33 | 1.06, 1.68 | 0.015 | 1.43 | 0.97, 2.12 | 0.07 | 1.20 | 1.03, 1.51 | 0.025 | 272.8 | <0.001 |
| Oesophagus | 0.98 | 0.41, 2.32 | 0.96 | 1.14 | 0.41, 3.18 | 0.80 | 1.71 | 0.73, 5.52 | 0.19 | 111.7 | 0.08 |
| Barrett's oesophagus | 1.15 | 0.55, 2.41 | 0.71 | 1.21 | 0.54, 2.73 | 0.65 | 1.27 | 0.76, 2.10 | 0.36 | 104.5 | 0.18 |

Estimates represent odds ratios per 1 standard deviation increase in log-transformed number of drinks per week. *Heterogeneity test statistics from the inverse-variance weighted method.

**Table S14.** Between-study heterogeneity in primary estimates: Q statistics and associated p-values

|  | **Unadjusted** | | **Adjusted for smoking** | | ***ADH1B* variant only** | |
| --- | --- | --- | --- | --- | --- | --- |
| Cancer type/site | Q statistic | p-value | Q statistic | p-value | Q statistic | p-value |
| All cancer | 0.00 | 0.98 | 0.00 | 0.98 | 5.49 | 0.02 |
| All gastrointestinal | 0.28 | 0.60 | 0.70 | 0.40 | 1.42 | 0.23 |
| Bladder | 3.08 | 0.38 | 4.22 | 0.24 | 1.01 | 0.80 |
| Brain | 4.03 | 0.26 | 5.58 | 0.13 | 3.31 | 0.35 |
| Breast | 1.46 | 0.69 | 0.82 | 0.84 | 2.72 | 0.44 |
| Cervix | 0.24 | 0.89 | 0.22 | 0.89 | 0.35 | 0.84 |
| Colorectum | 3.80 | 0.28 | 3.57 | 0.31 | 9.12 | 0.03 |
| Head/neck | 3.61 | 0.31 | 2.61 | 0.46 | 4.82 | 0.19 |
| Kidney | 1.16 | 0.76 | 1.10 | 0.78 | 4.55 | 0.21 |
| Leukaemia | 1.86 | 0.60 | 0.72 | 0.87 | 1.75 | 0.63 |
| Liver | 3.69 | 0.30 | 2.87 | 0.41 | 3.78 | 0.29 |
| Lung | 6.87 | 0.08 | 5.49 | 0.14 | 8.24 | 0.04 |
| Melanoma | 1.19 | 0.75 | 4.05 | 0.26 | 1.53 | 0.68 |
| Myeloma | 2.79 | 0.43 | 2.46 | 0.48 | 1.78 | 0.62 |
| Non-Hodgkin's lymphoma | 1.82 | 0.61 | 1.41 | 0.70 | 2.41 | 0.49 |
| Oesophagus | 0.96 | 0.81 | 0.60 | 0.90 | 0.89 | 0.83 |
| Ovaries | 3.72 | 0.16 | 2.79 | 0.25 | 9.04 | 0.01 |
| Pancreas | 4.14 | 0.25 | 0.50 | 0.92 | 1.78 | 0.62 |
| Prostate | 0.38 | 0.95 | 0.80 | 0.85 | 1.90 | 0.59 |
| Stomach | 2.97 | 0.40 | 0.50 | 0.92 | 0.10 | 0.99 |
| Testes | 2.35 | 0.50 | 2.97 | 0.40 | 4.28 | 0.23 |
| Uterus | 1.41 | 0.49 | 1.75 | 0.42 | 0.61 | 0.74 |
