# Supplementary material for: Alcohol consumption and risk of cancer: a Mendelian randomization analysis of four biobanks and consortium data
Source: BMC Med. 2025 Dec 16;23:676. doi: 10.1186/s12916-025-04543-8 (PMC12707013; doi:10.1186/s12916-025-04543-8)

**Additional file 2**

**Fig. S1.** Mendelian randomization estimates calculated using outcome data on All of US African ancestry participants.

**Fig. S2.** Mendelian randomization estimates calculated using outcome data on Million Veteran Program African ancestry participants.

**Fig. S3.** Mendelian randomization estimates calculated using outcome data on All of US American admixed ancestry participants.

**Fig. S4.** Mendelian randomization estimates calculated using outcome data on Million Veteran Program American admixed ancestry participants.

**Fig. S5.** Mendelian randomization estimates calculated using outcome data on UK Biobank European ancestry participants.

**Fig. S6.** Mendelian randomization estimates calculated using outcome data on FinnGen participants.

**Fig. S7.** Mendelian randomization estimates calculated using outcome data on All of US European ancestry participants.

**Fig. S8.** Mendelian randomization estimates calculated using outcome data on Million Veteran Program European ancestry participants.

**Fig. S9.** Genetic associations with alcohol consumption and with risk of any breast cancer from consortium data.

**Fig. S10.** Genetic associations with alcohol consumption and with risk of triple negative breast cancer from consortium data.

**Fig. S11.** Genetic associations with alcohol consumption and with risk of triple negative or BRCA+ breast cancer from consortium data.

**Fig. S12.** Genetic associations with alcohol consumption and with risk of luminal A breast cancer from consortium data.

**Fig. S13.** Genetic associations with alcohol consumption and with risk of luminal B breast cancer from consortium data.

**Fig. S14.** Genetic associations with alcohol consumption and with risk of luminal B or HER2- breast cancer from consortium data.

**Fig. S15.** Genetic associations with alcohol consumption and with risk of HER2 enriched breast cancer from consortium data.

**Fig. S16.** Genetic associations with alcohol consumption and with risk of breast cancer survival (Escala-Garcia 2019) from consortium data.

**Fig. S17.** Genetic associations with alcohol consumption and with risk of breast cancer survival (Morra 2021) from consortium data.

**Fig. S18.** Genetic associations with alcohol consumption and with risk of non-mucinous ovarian cancer from consortium data.

**Fig. S19.** Genetic associations with alcohol consumption and with risk of mucinous ovarian cancer from consortium data.

**Fig. S20.** Genetic associations with alcohol consumption and with risk of high grade serous ovarian cancer from consortium data.

**Fig. S21.** Genetic associations with alcohol consumption and with risk of low grade serous ovarian cancer from consortium data.

**Fig. S22.** Genetic associations with alcohol consumption and with risk of endometrioid ovarian cancer from consortium data.

**Fig. S23.** Genetic associations with alcohol consumption and with risk of clear cell ovarian cancer from consortium data.

**Fig. S24.** Genetic associations with alcohol consumption and with risk of endometrial cancer from consortium data.

**Fig. S25.** Genetic associations with alcohol consumption and with risk of prostate cancer from consortium data.

**Fig. S26.** Genetic associations with alcohol consumption and with risk of any kidney cancer from consortium data.

**Fig. S27.** Genetic associations with alcohol consumption and with risk of clear renal cell carcinoma from consortium data.

**Fig. S28.** Genetic associations with alcohol consumption and with risk of papillary renal cell carcinoma from consortium data.

**Fig. S29.** Genetic associations with alcohol consumption and with risk of colorectum cancer from consortium data

**Fig. S30.** Genetic associations with alcohol consumption and with risk of oesophagus cancer from consortium data.

**Fig. S31.** Genetic associations with alcohol consumption and with risk of Barrett's oesophagus from consortium data.

**Fig. S1.** Mendelian randomization estimates calculated using outcome data on All of US African ancestry participants


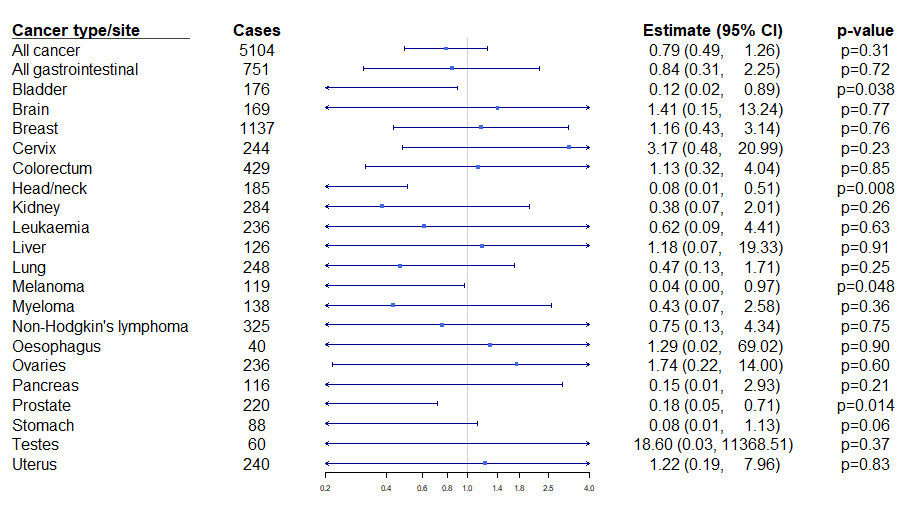


Estimates represent odds ratios per 1 standard deviation increase in log-transformed number of drinks per week. Error bars are 95% confidence intervals.

**Fig. S2.** Mendelian randomization estimates calculated using outcome data on Million Veteran Program African ancestry participants


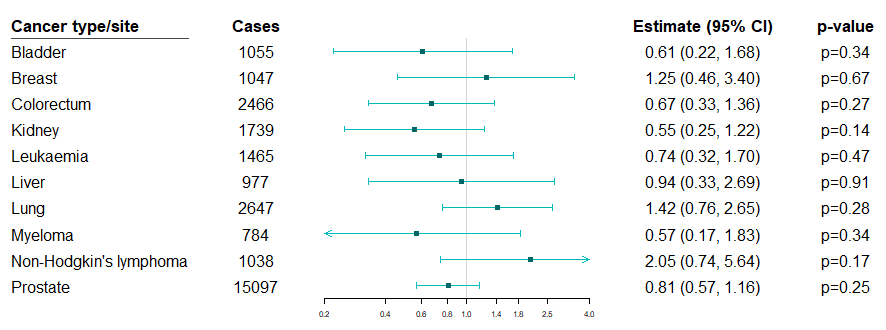


Estimates represent odds ratios per 1 standard deviation increase in log-transformed number of drinks per week. Error bars are 95% confidence intervals.

**Fig. S3.** Mendelian randomization estimates calculated using outcome data on All of US American admixed ancestry participants


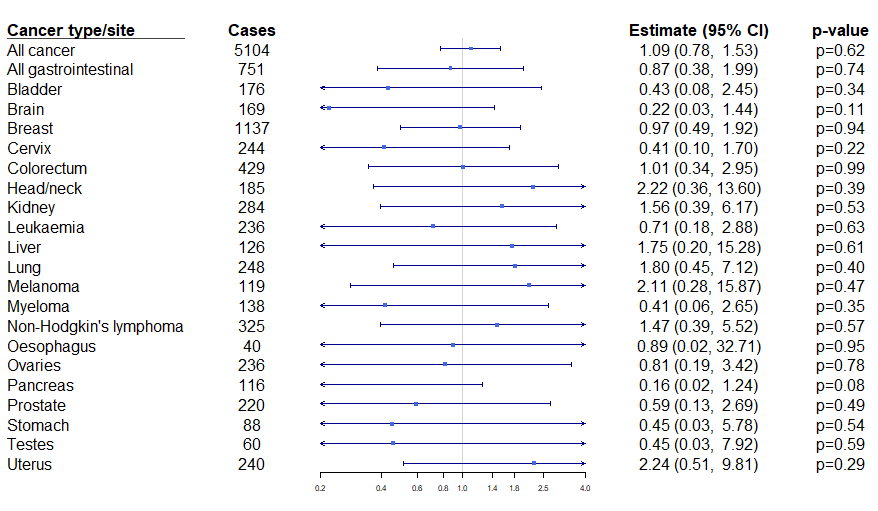


Estimates represent odds ratios per 1 standard deviation increase in log-transformed number of drinks per week. Error bars are 95% confidence intervals.

**Fig. S4.** Mendelian randomization estimates calculated using outcome data on Million Veteran Program American admixed ancestry participants


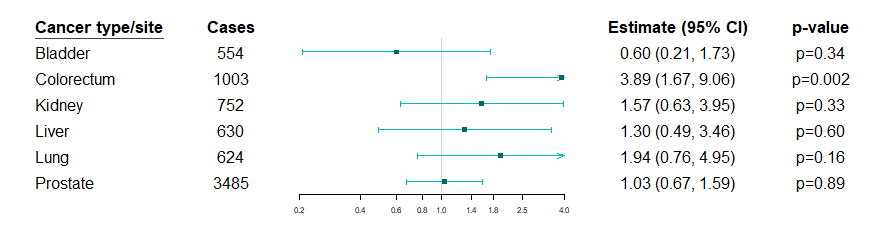


**Fig. S5.** Mendelian randomization estimates calculated using outcome data on UK Biobank European ancestry participants


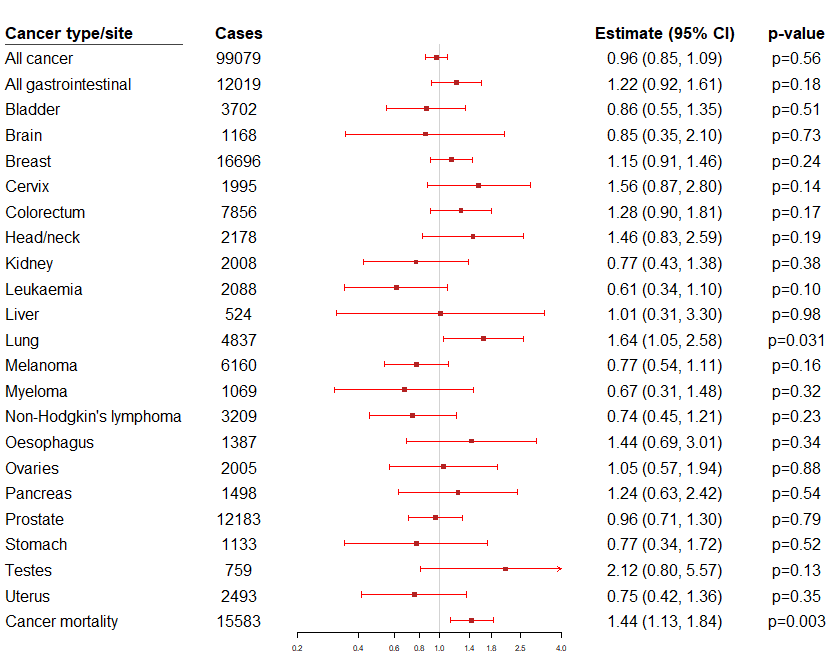


Estimates represent odds ratios per 1 standard deviation increase in log-transformed number of drinks per week. Error bars are 95% confidence intervals.

**Fig. S6.** Mendelian randomization estimates calculated using outcome data on FinnGen participants


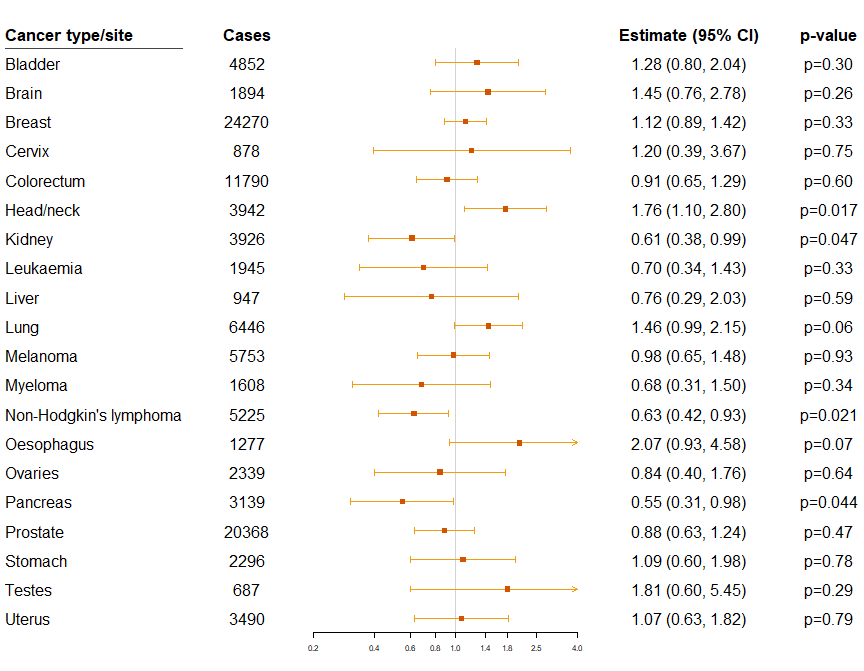


Estimates represent odds ratios per 1 standard deviation increase in log-transformed number of drinks per week. Error bars are 95% confidence intervals.

**Fig. S7.** Mendelian randomization estimates calculated using outcome data on All of US European ancestry participants


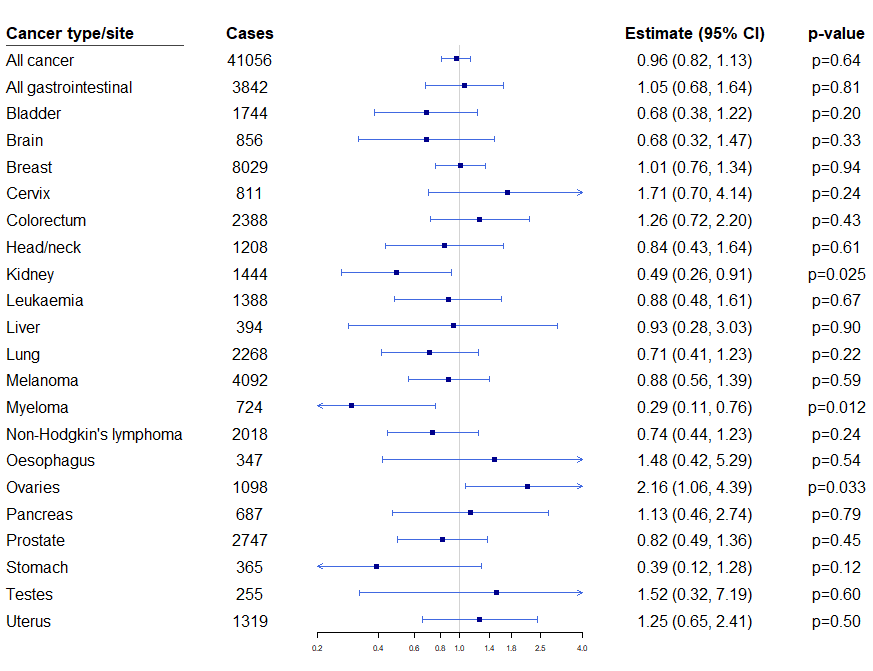


Estimates represent odds ratios per 1 standard deviation increase in log-transformed number of drinks per week. Error bars are 95% confidence intervals.

**Fig. S8.** Mendelian randomization estimates calculated using outcome data on Million Veteran Program European ancestry participants


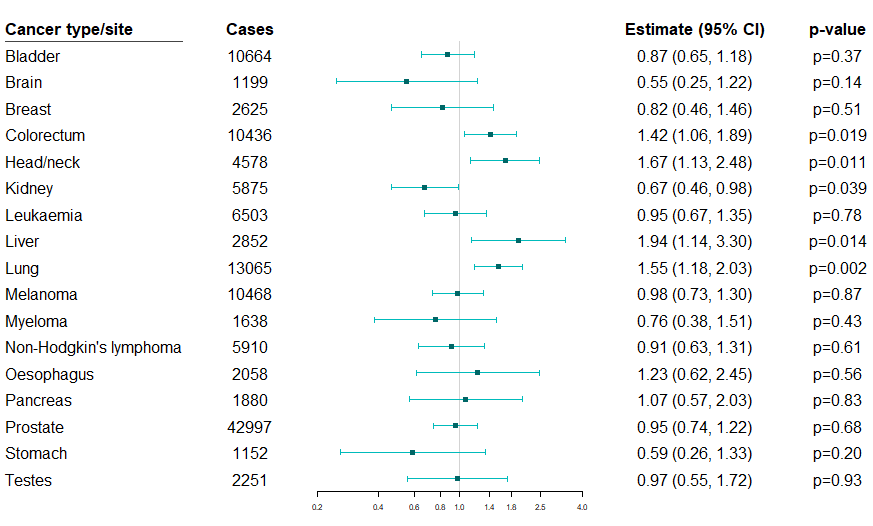


**Fig. S9.** Genetic associations with alcohol consumption and with risk of any breast cancer from consortium data


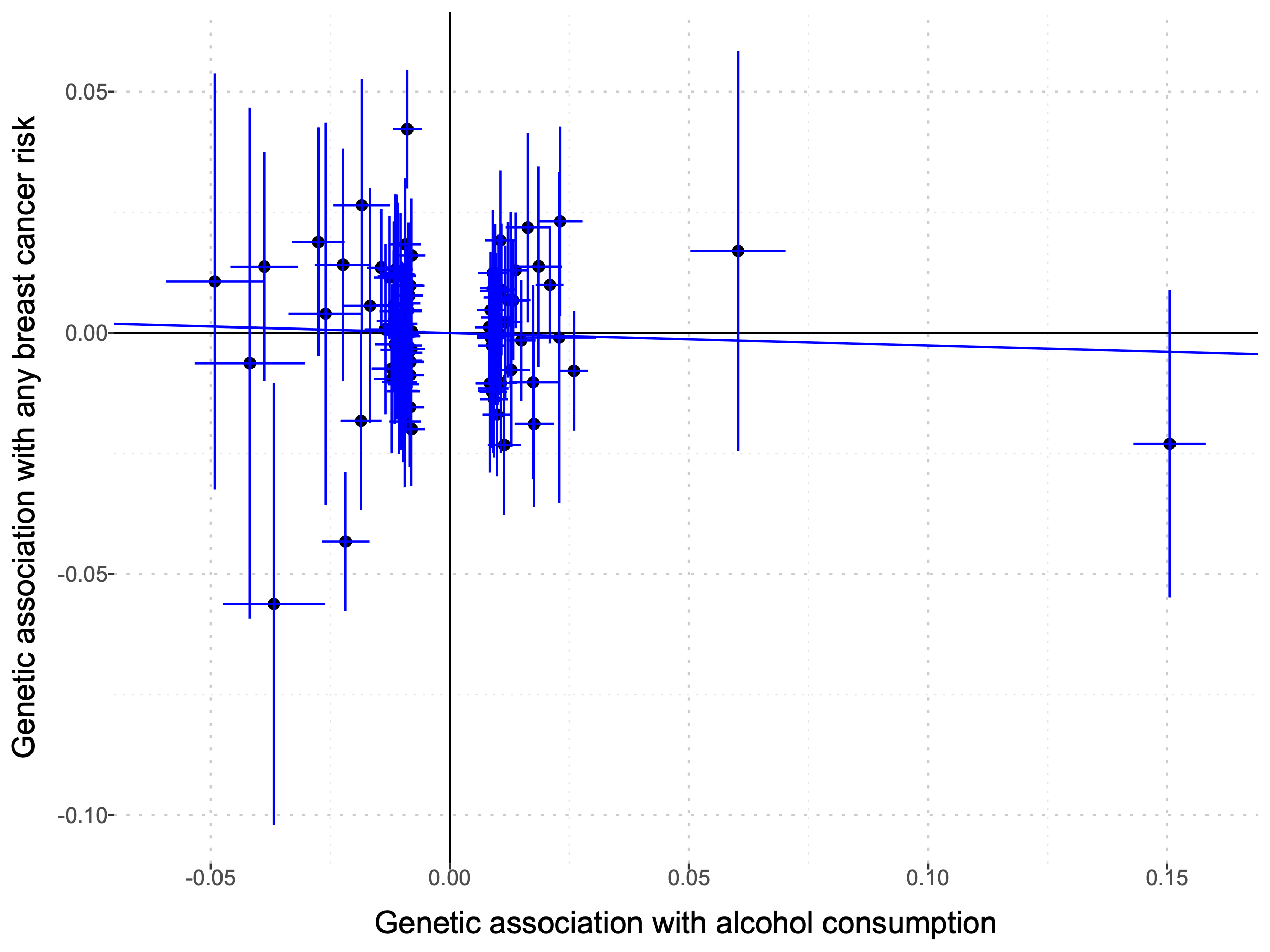


**Fig. S10.** Genetic associations with alcohol consumption and with risk of triple negative breast cancer from consortium data


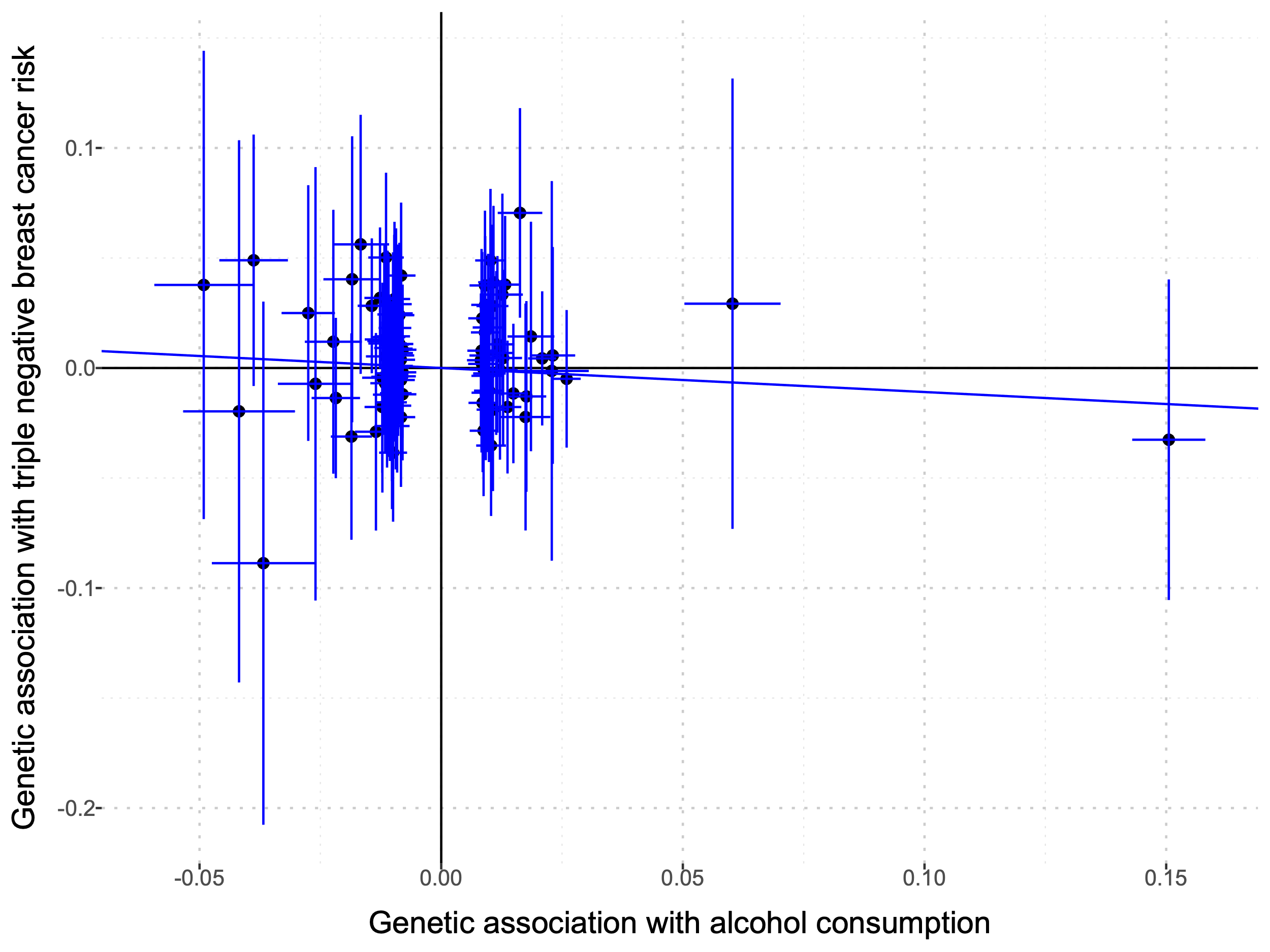


**Fig. S11.** Genetic associations with alcohol consumption and with risk of triple negative or BRCA+ breast cancer from consortium data


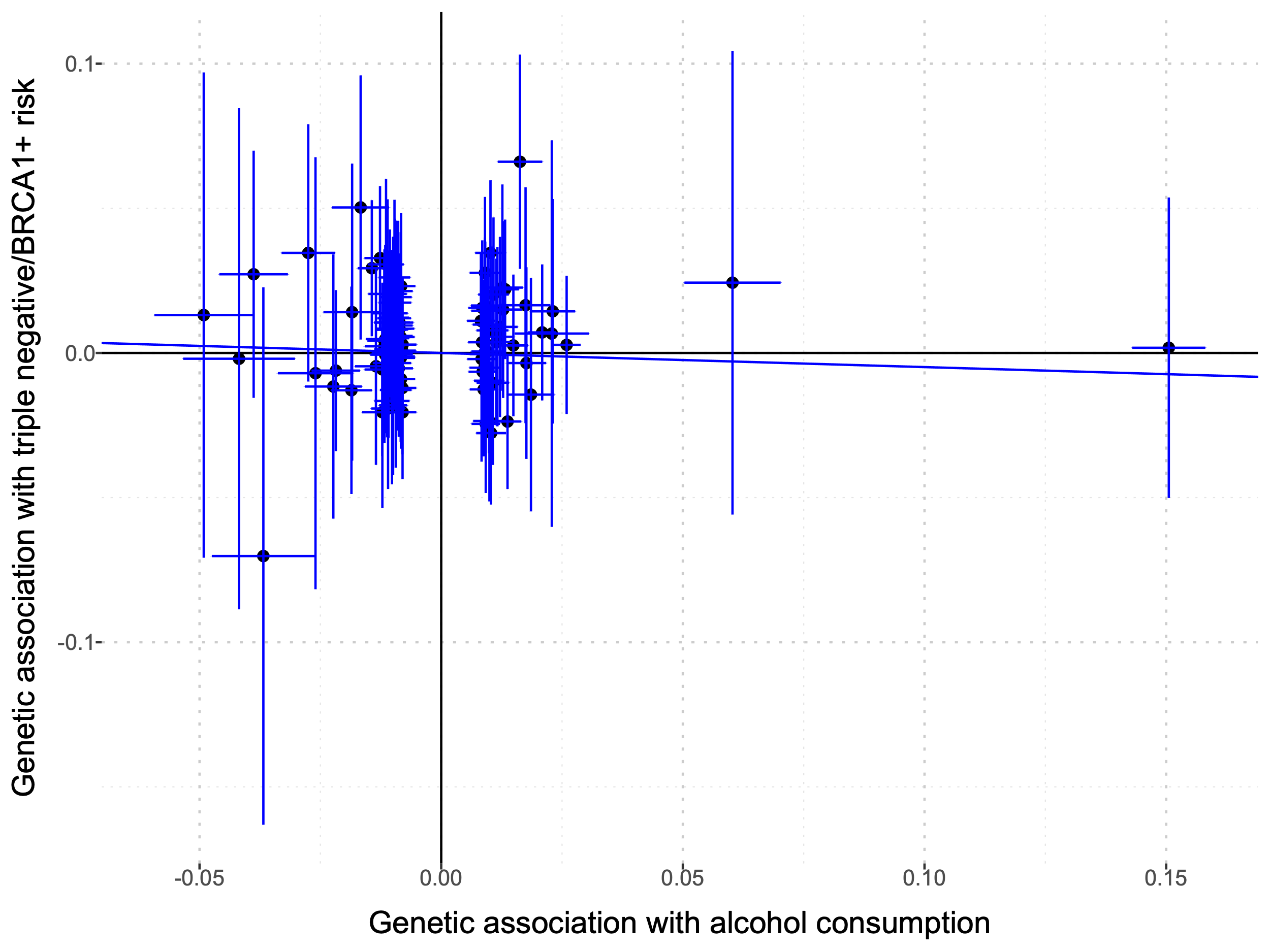


**Fig. S12.** Genetic associations with alcohol consumption and with risk of luminal A breast cancer from consortium data


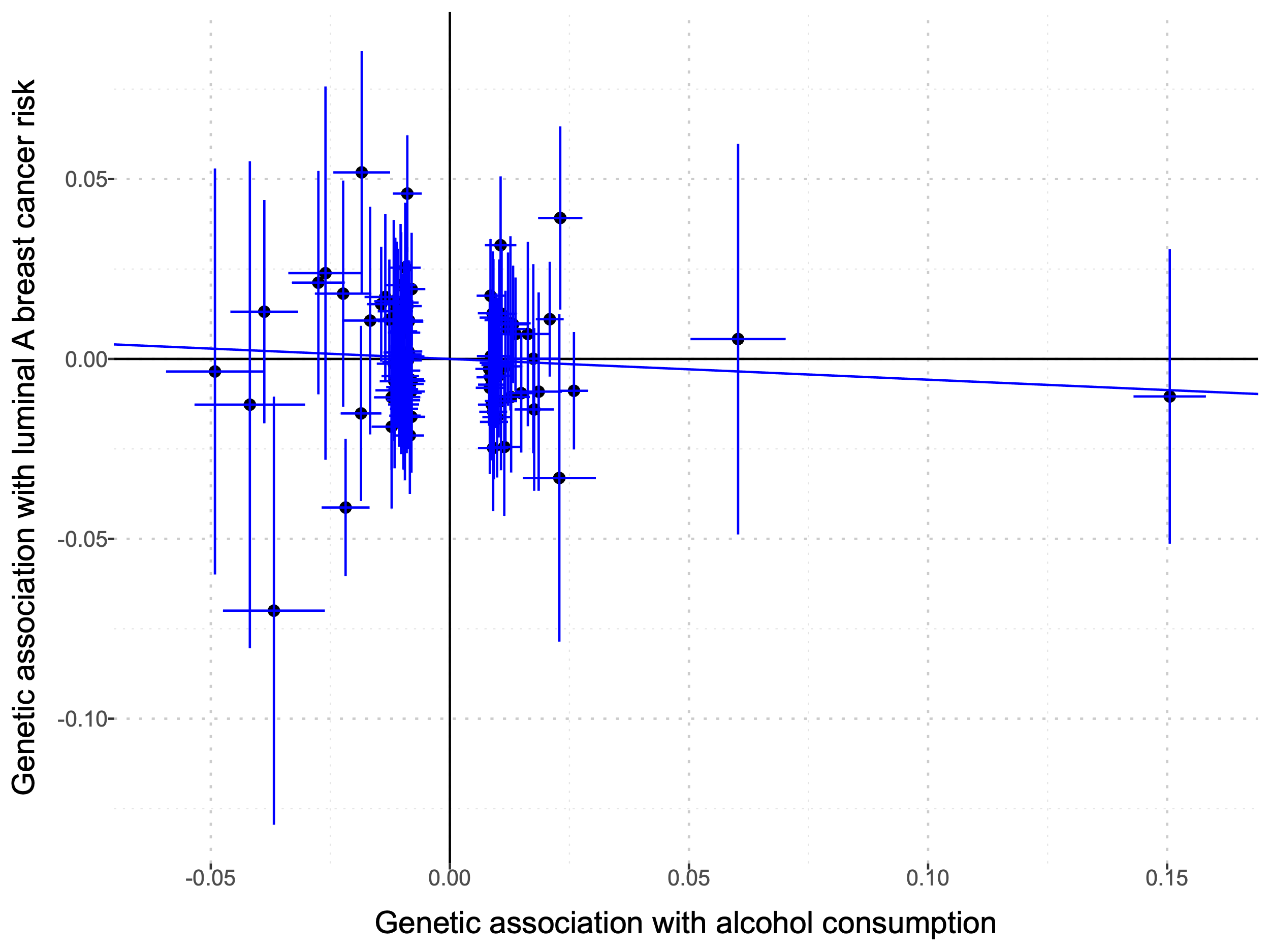


**Fig. S13.** Genetic associations with alcohol consumption and with risk of luminal B breast cancer from consortium data


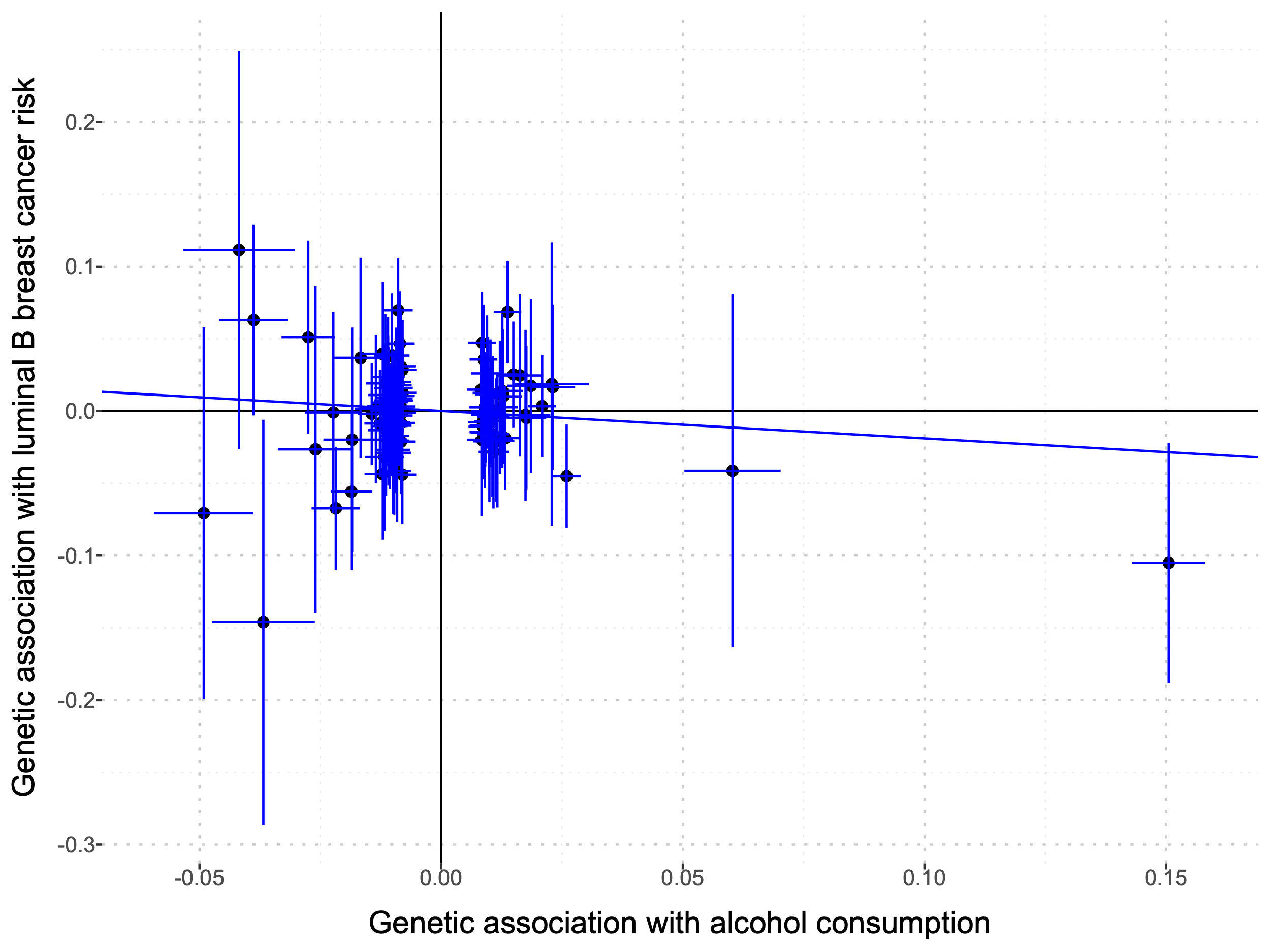


**Fig. S14.** Genetic associations with alcohol consumption and with risk of luminal B or HER2- breast cancer from consortium data


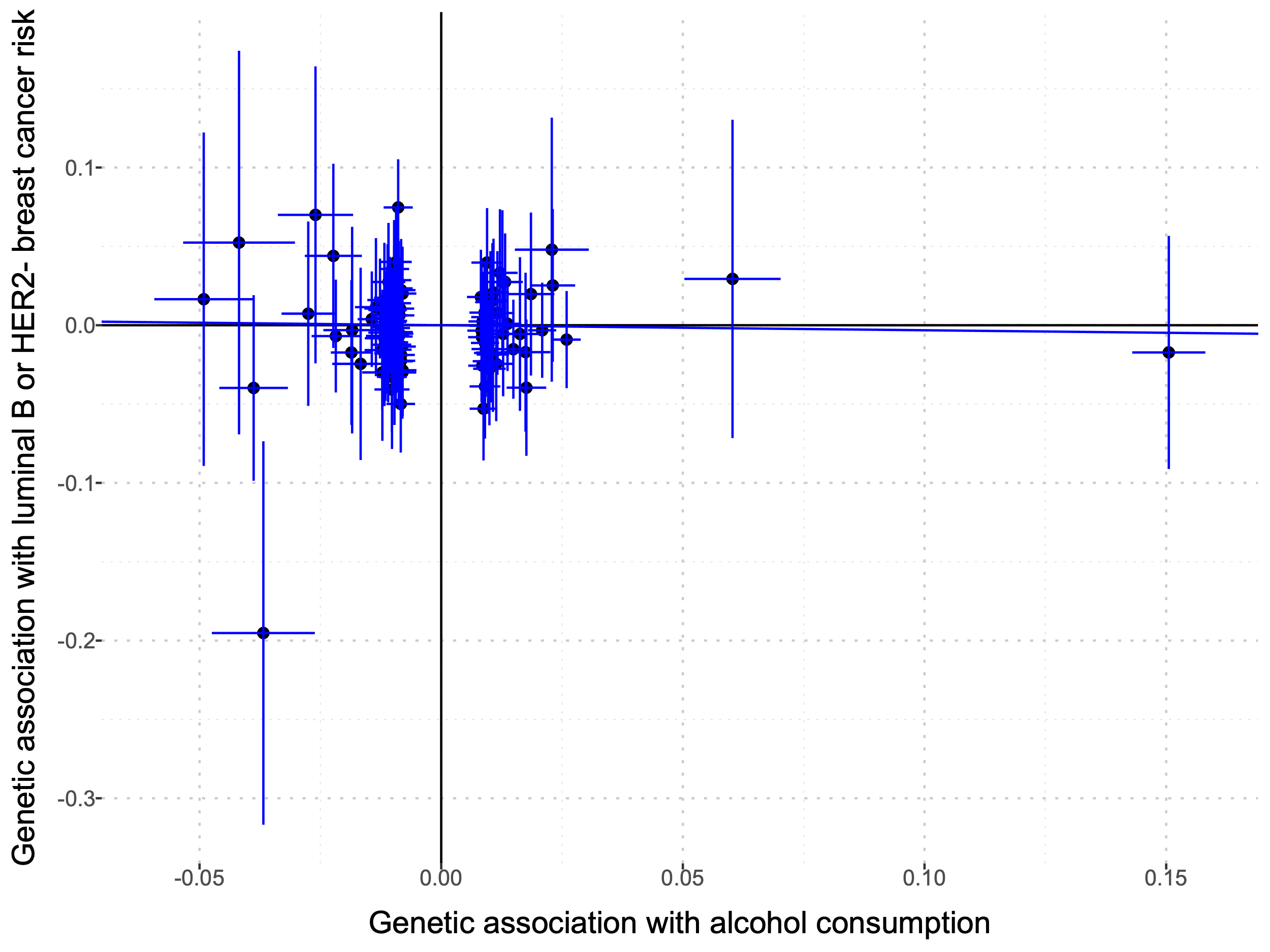


**Fig. S15.** Genetic associations with alcohol consumption and with risk of HER2 enriched breast cancer from consortium data


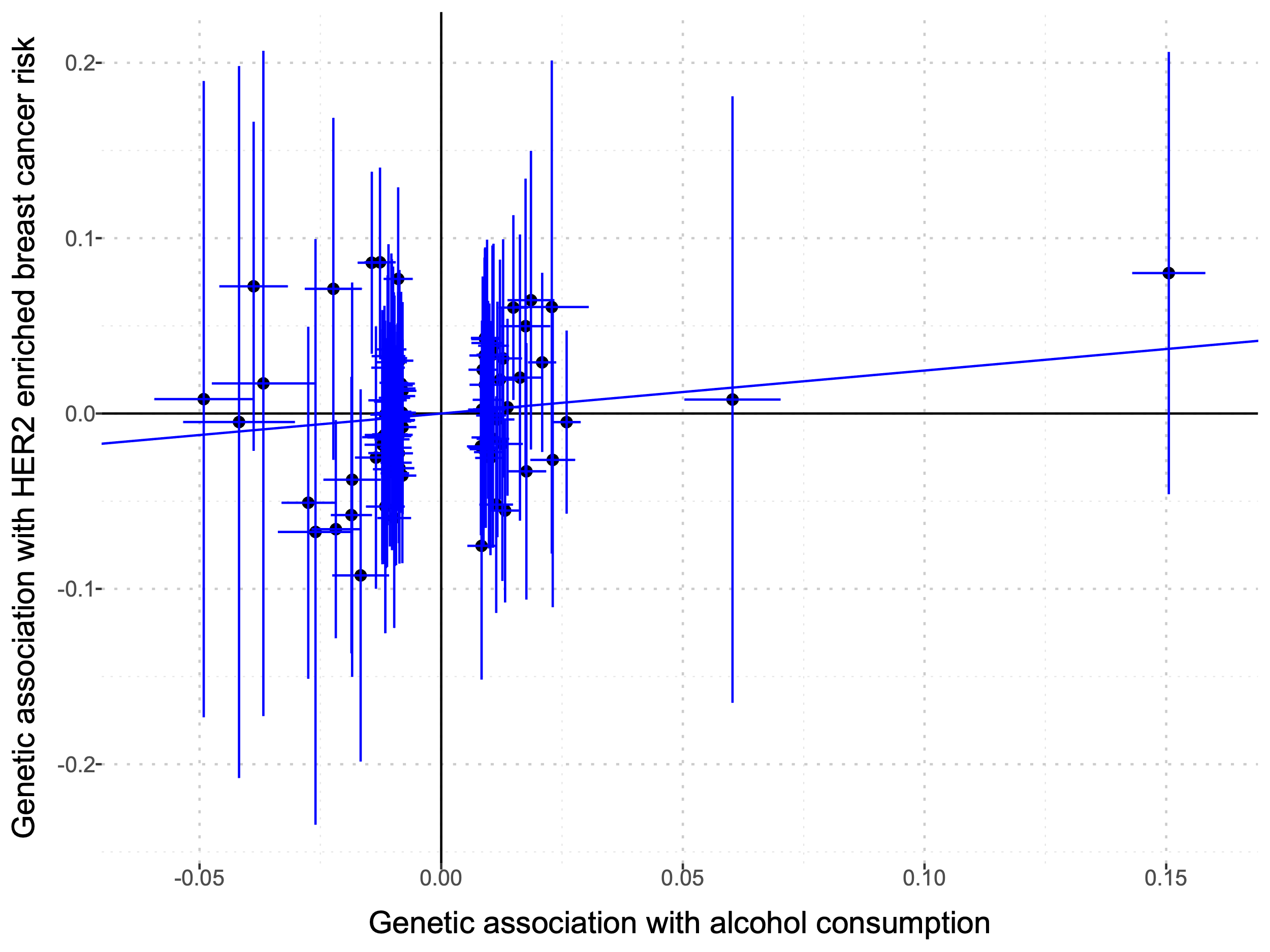


**Fig. S16.** Genetic associations with alcohol consumption and with risk of breast cancer survival (Escala-Garcia 2019) from consortium data


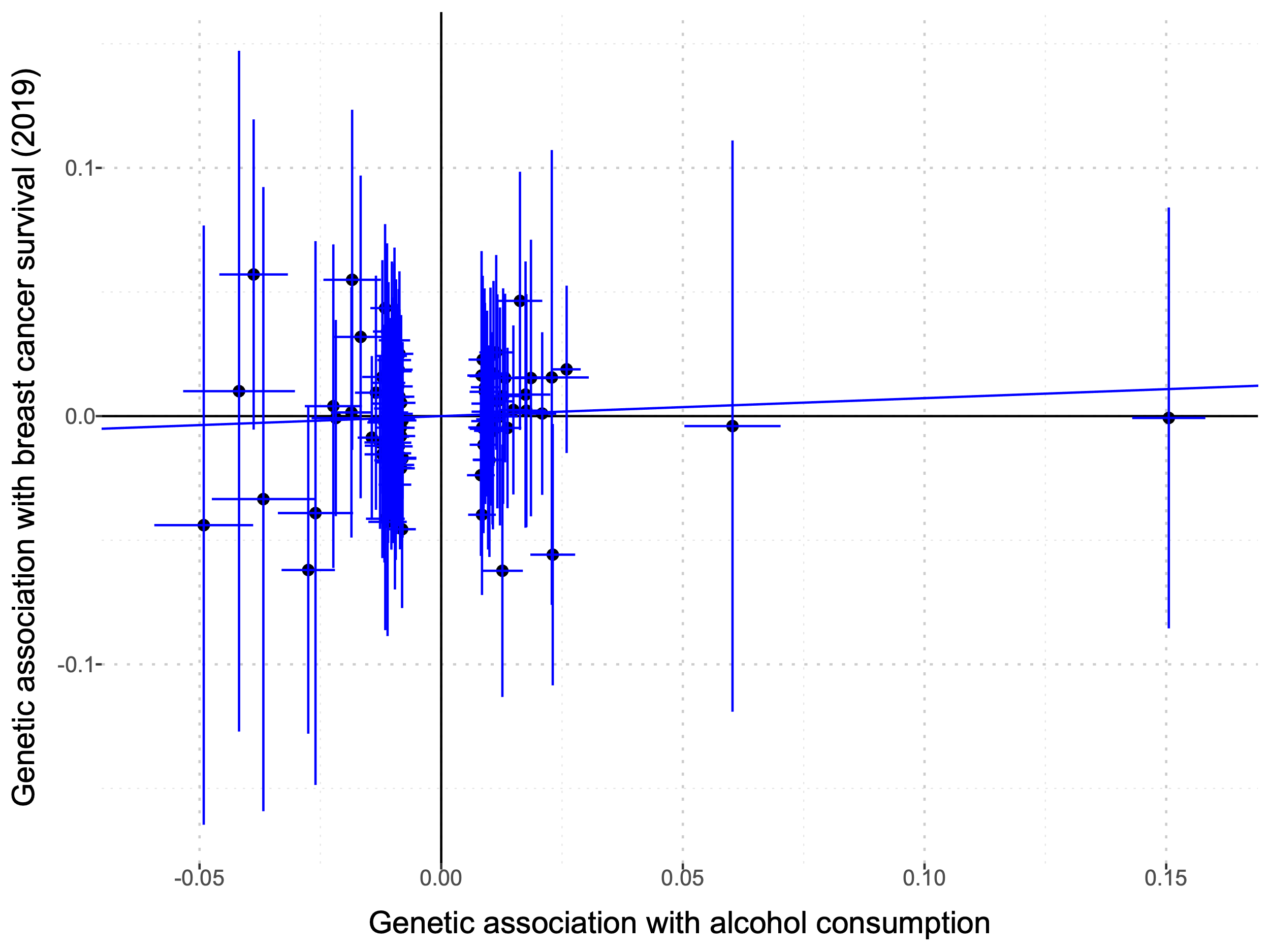


**Fig. S17.** Genetic associations with alcohol consumption and with risk of breast cancer survival (Morra 2021) from consortium data


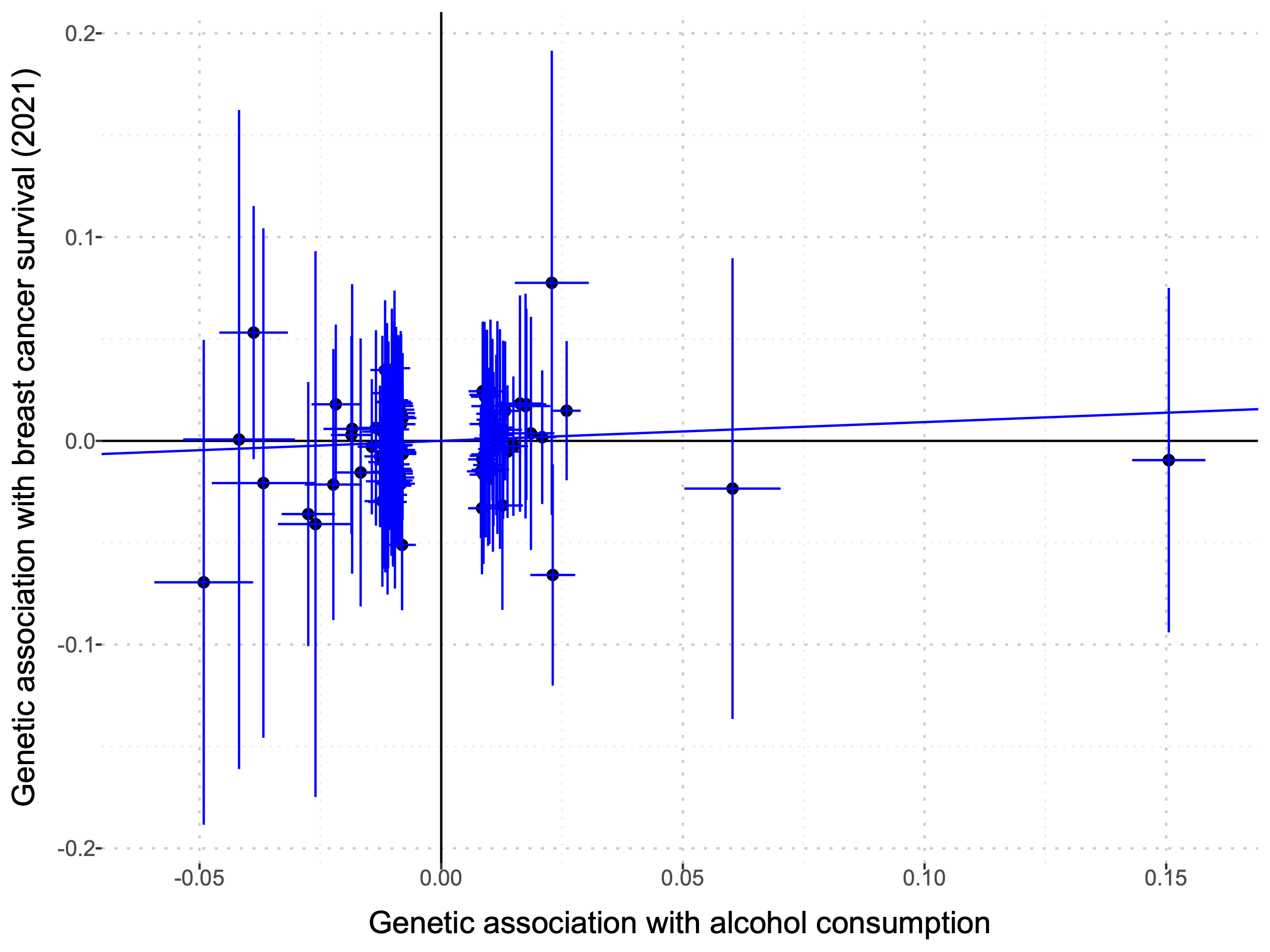


**Fig. S18.** Genetic associations with alcohol consumption and with risk of non-mucinous ovarian cancer from consortium data


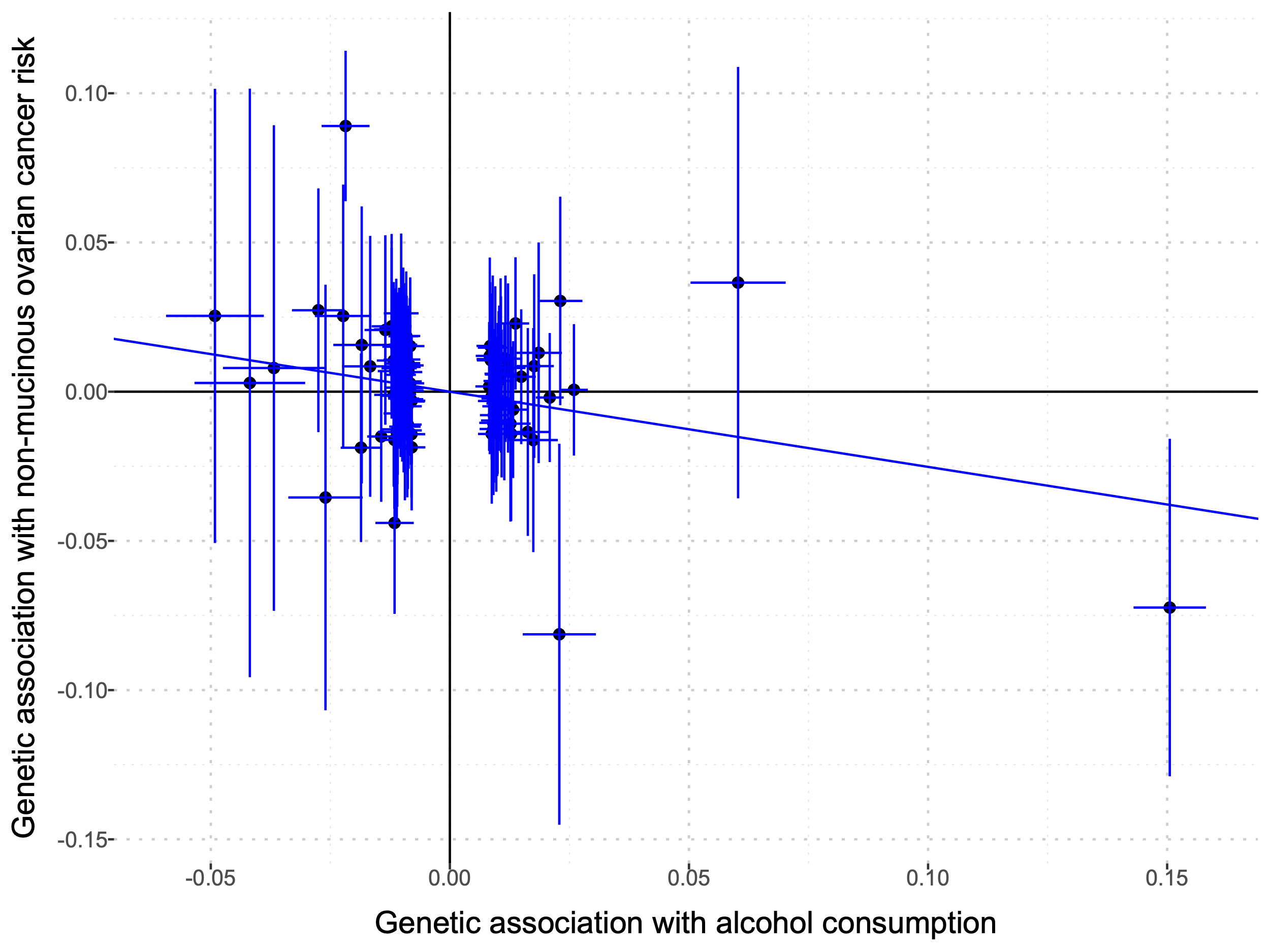


**Fig. S19.** Genetic associations with alcohol consumption and with risk of mucinous ovarian cancer from consortium data


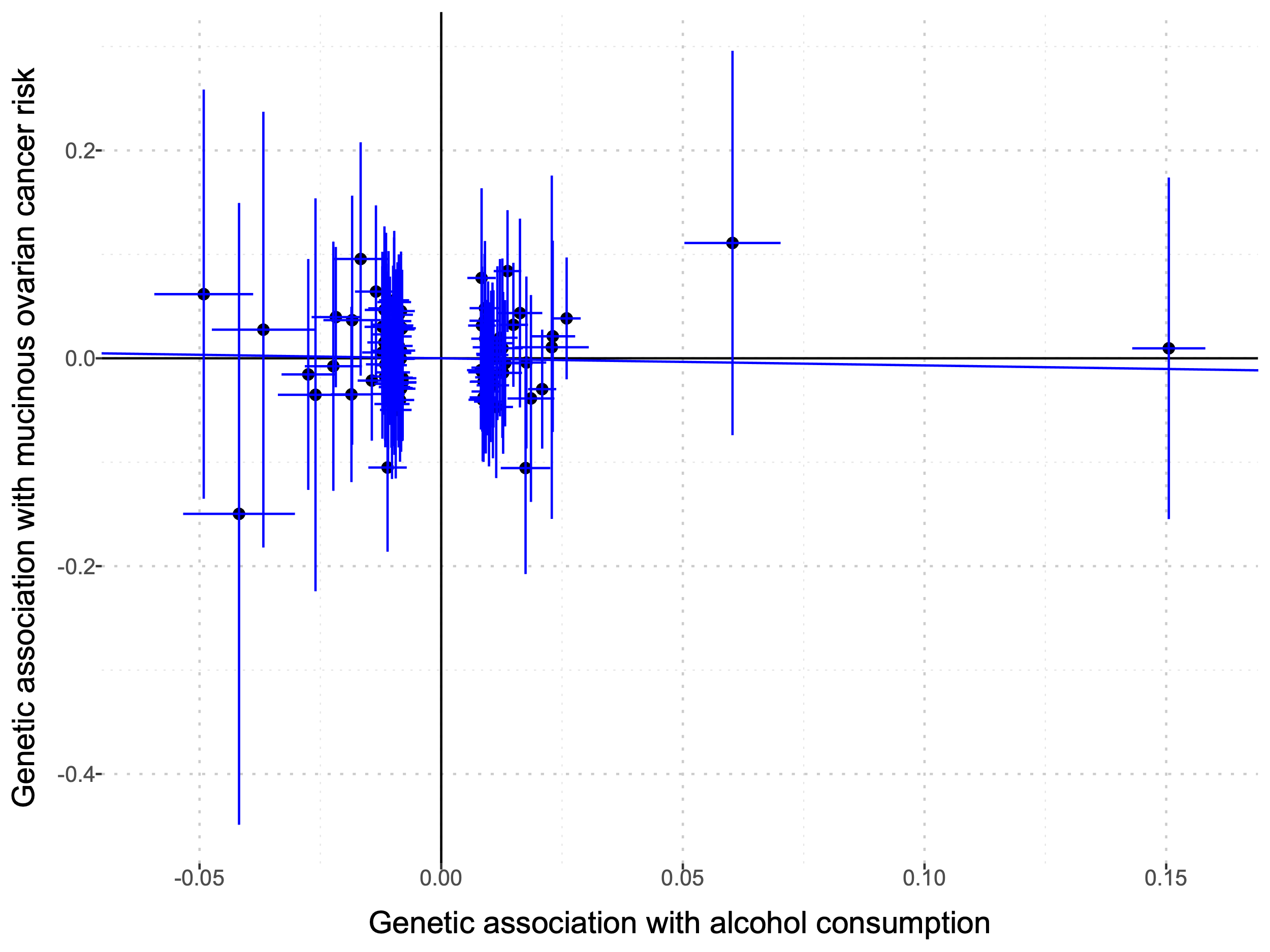


**Fig. S20.** Genetic associations with alcohol consumption and with risk of high grade serous ovarian cancer from consortium data


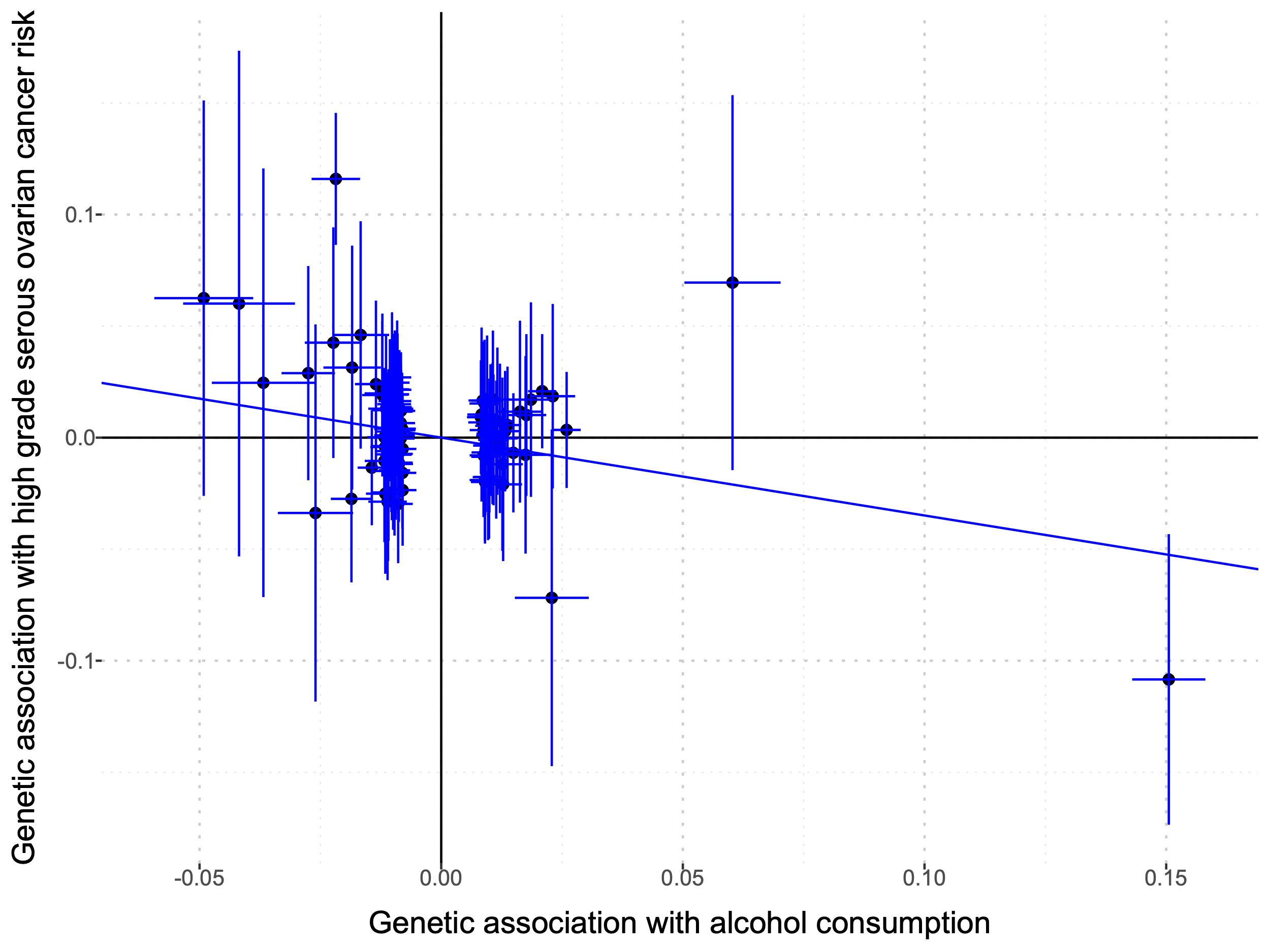


**Fig. S21.** Genetic associations with alcohol consumption and with risk of low grade serous ovarian cancer from consortium data


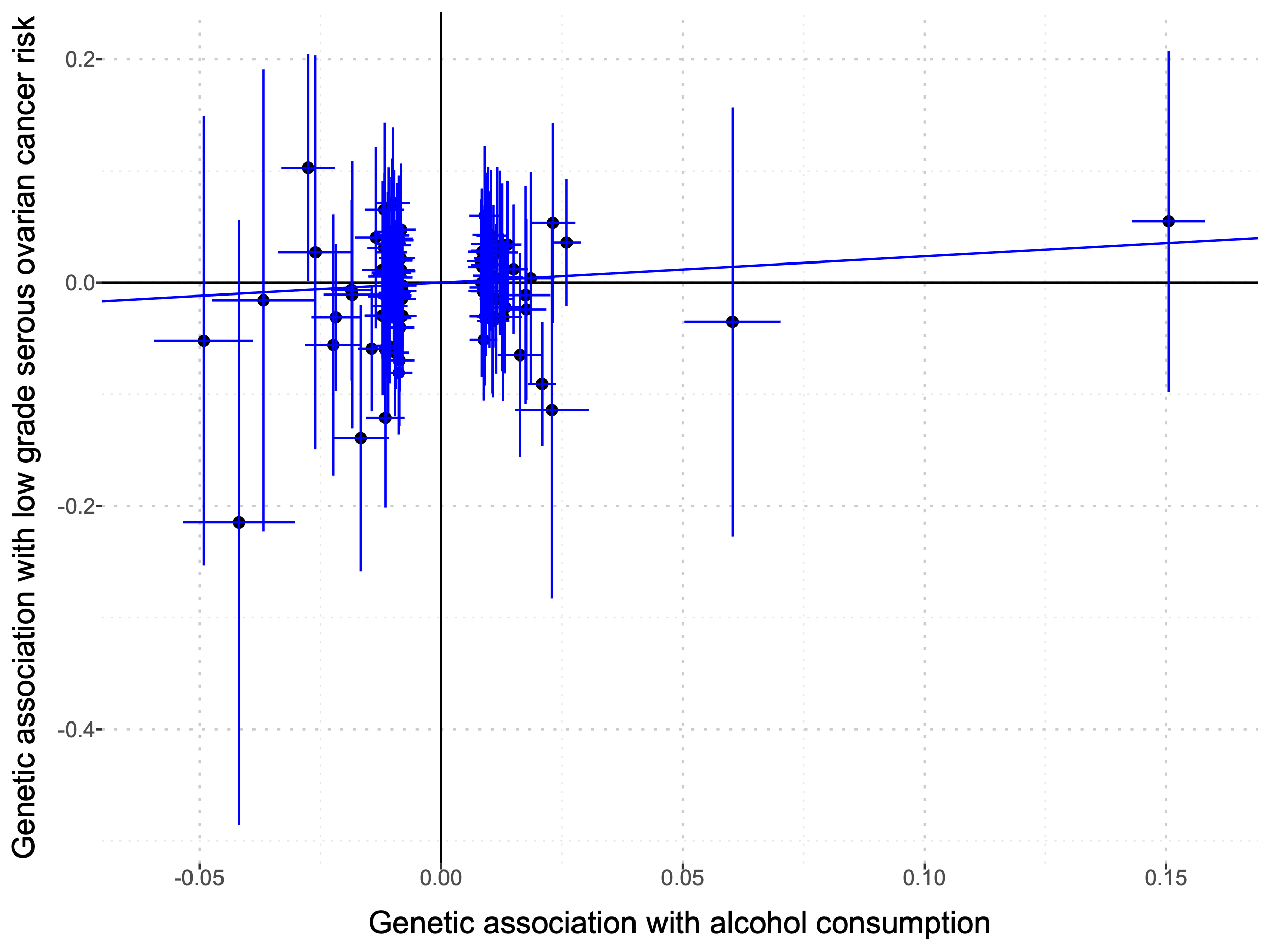


**Fig. S22.** Genetic associations with alcohol consumption and with risk of endometrioid ovarian cancer from consortium data


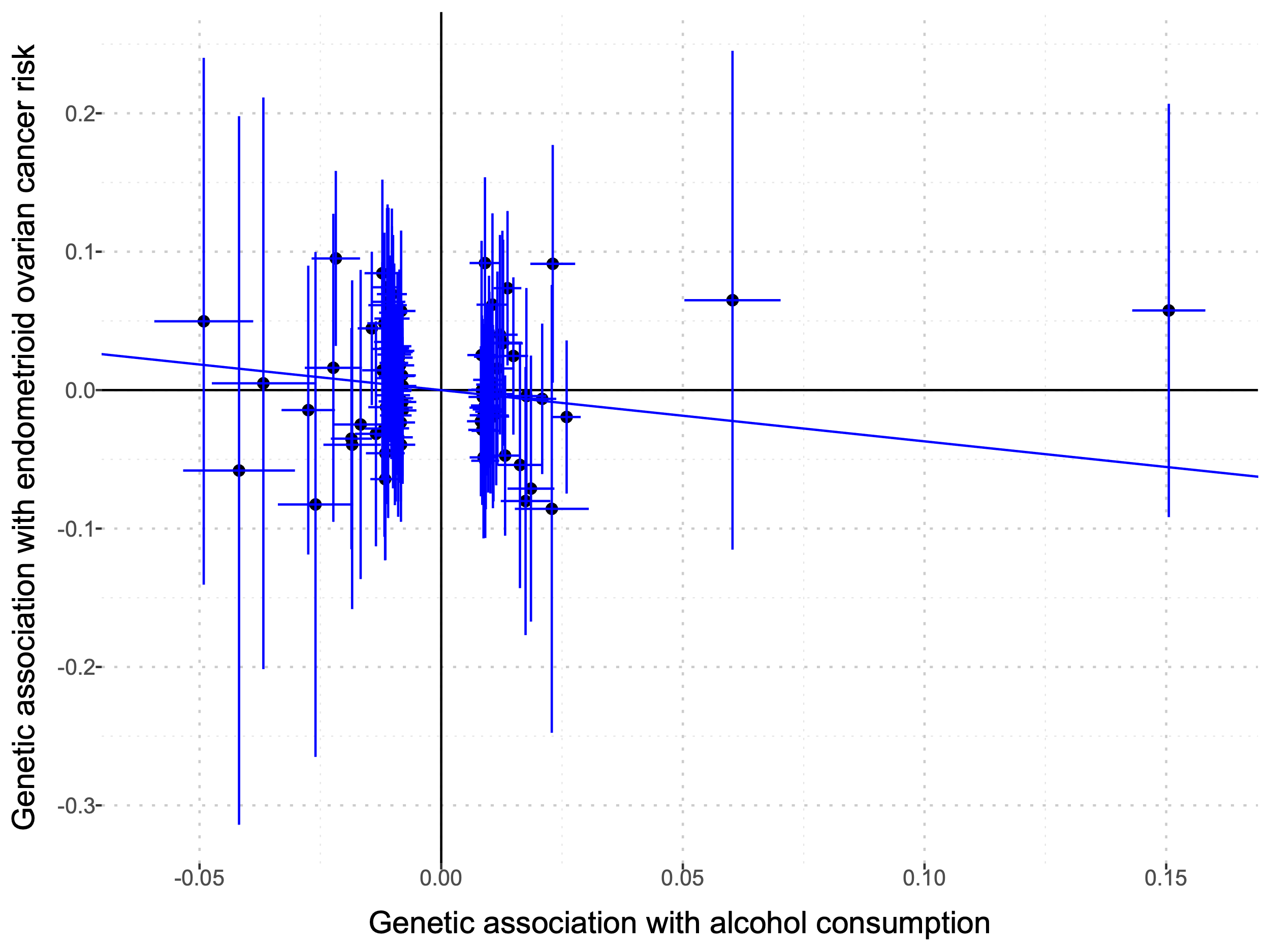


**Fig. S23.** Genetic associations with alcohol consumption and with risk of clear cell ovarian cancer from consortium data


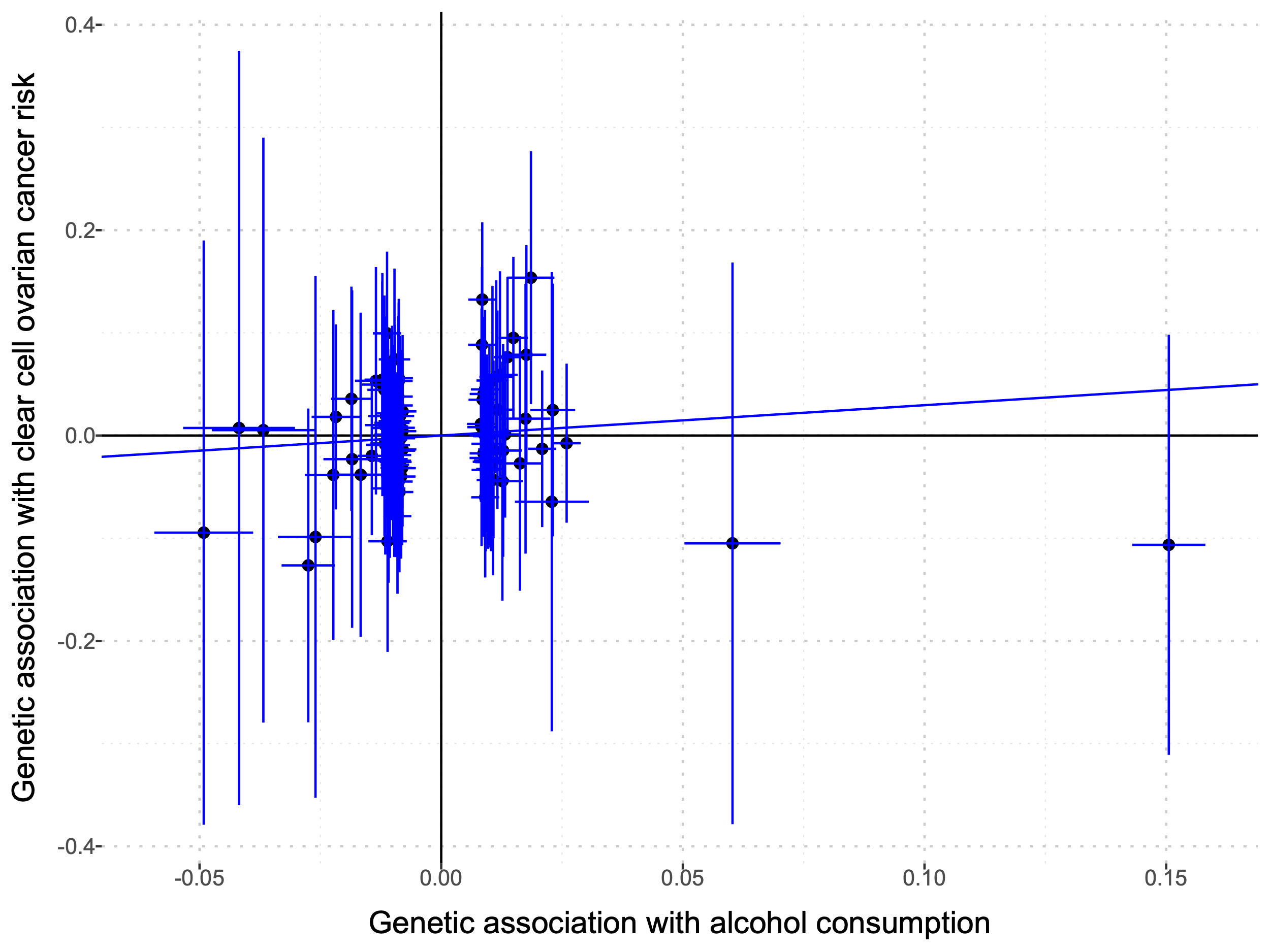


**Fig. S24.** Genetic associations with alcohol consumption and with risk of endometrial cancer from consortium data


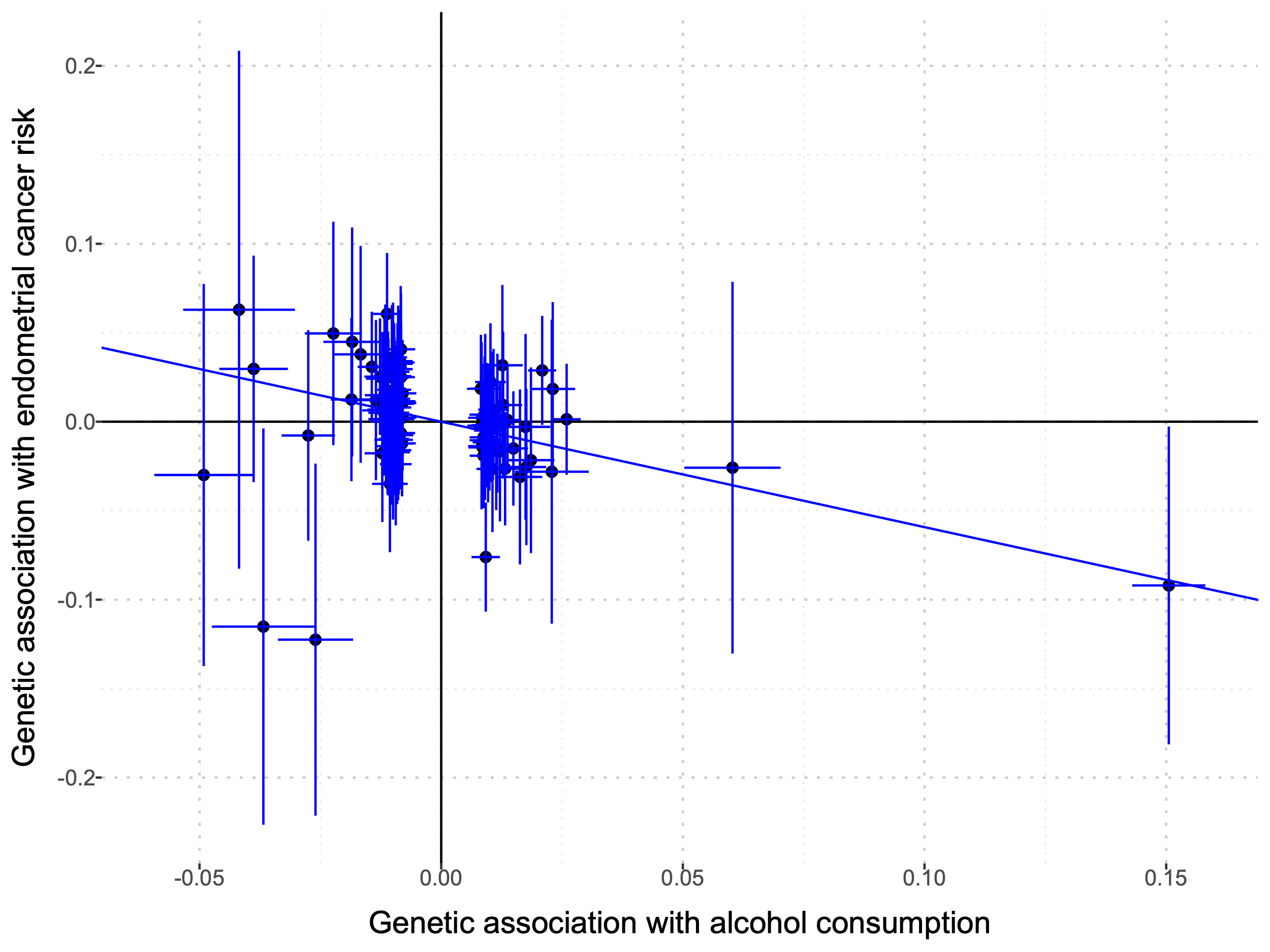


**Fig. S25.** Genetic associations with alcohol consumption and with risk of prostate cancer from consortium data


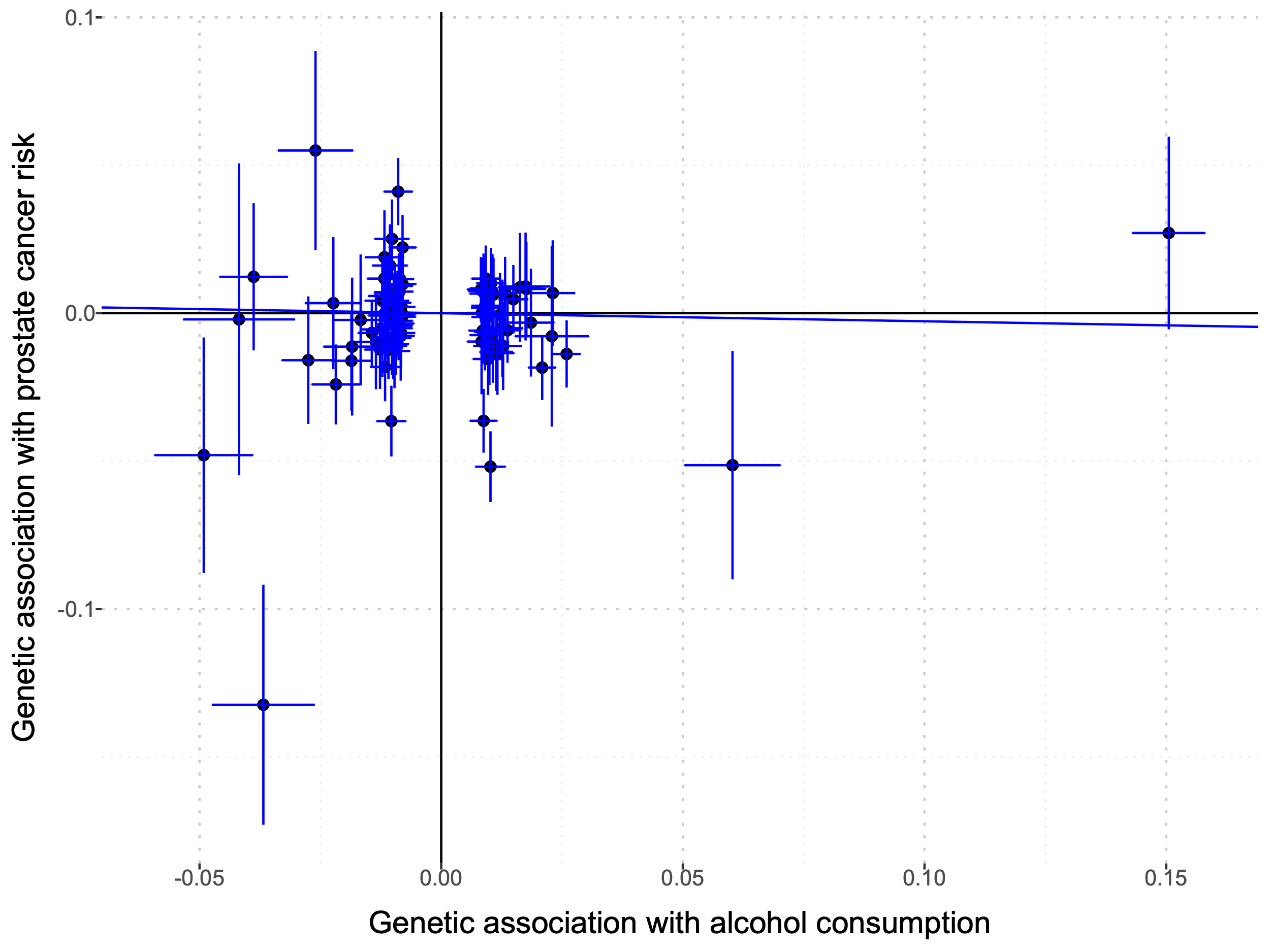


**Fig. S26.** Genetic associations with alcohol consumption and with risk of any kidney cancer from consortium data


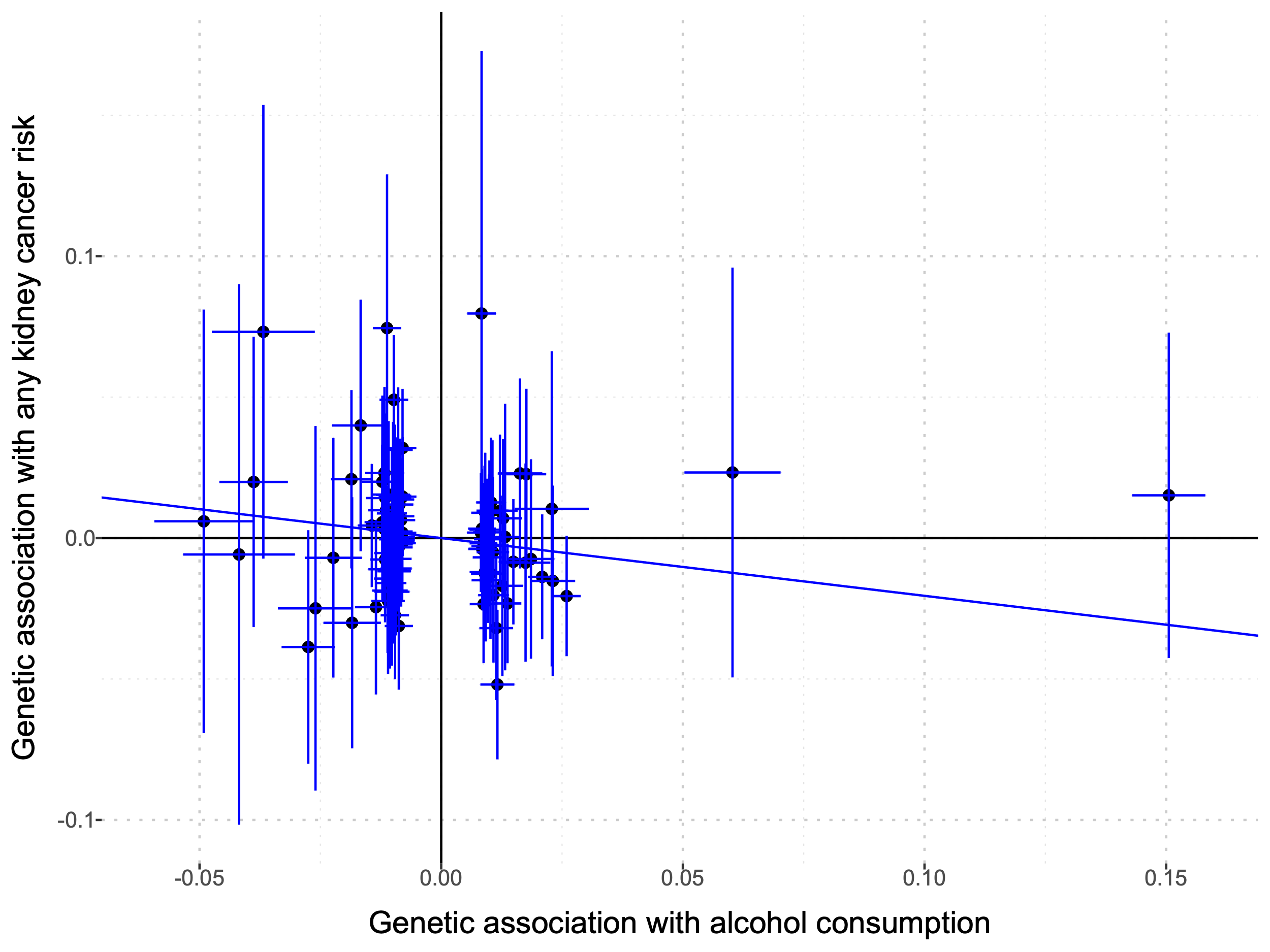


**Fig. S27.** Genetic associations with alcohol consumption and with risk of clear renal cell carcinoma from consortium data


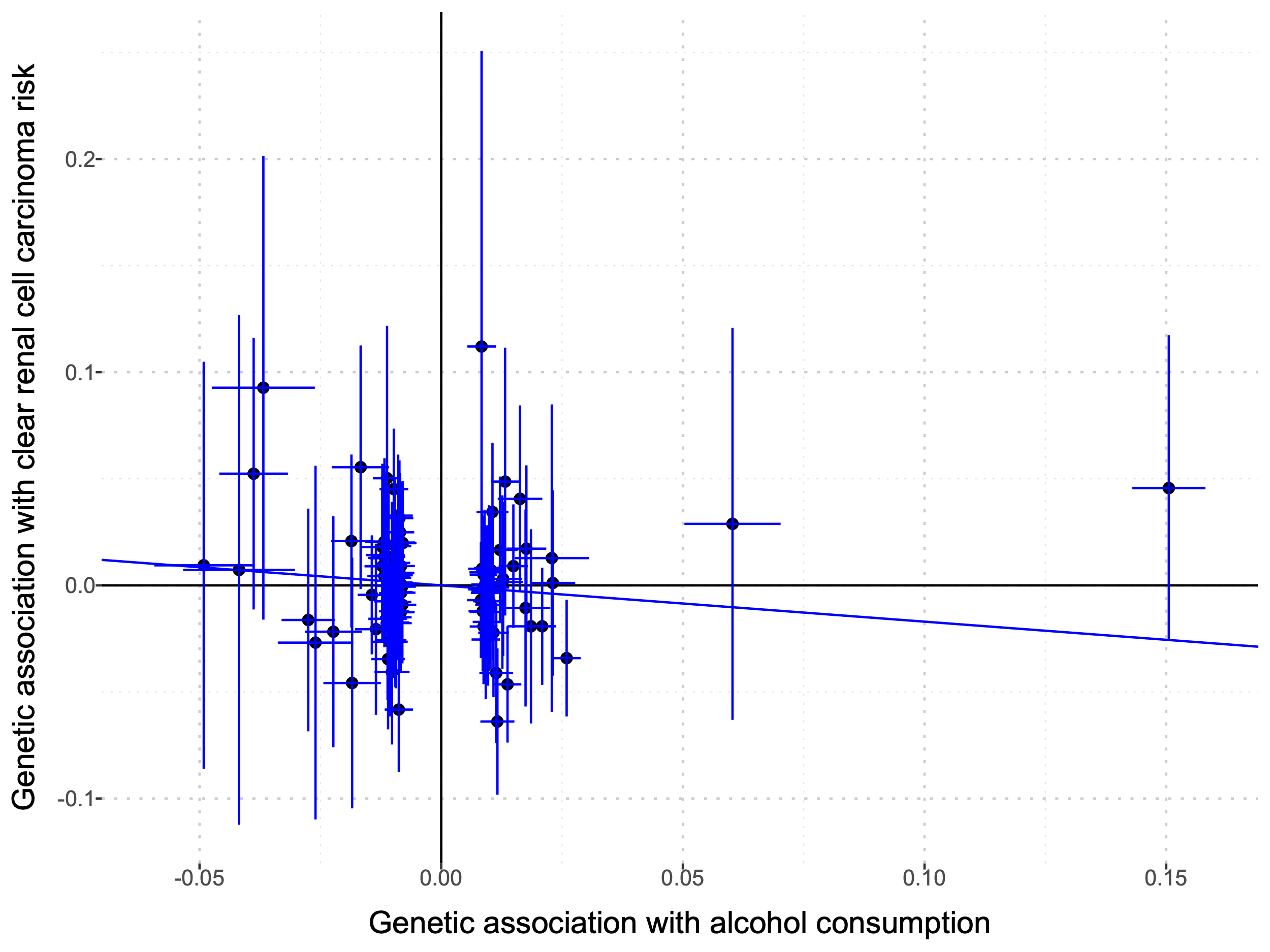


**Fig. S28.** Genetic associations with alcohol consumption and with risk of papillary renal cell carcinoma from consortium data


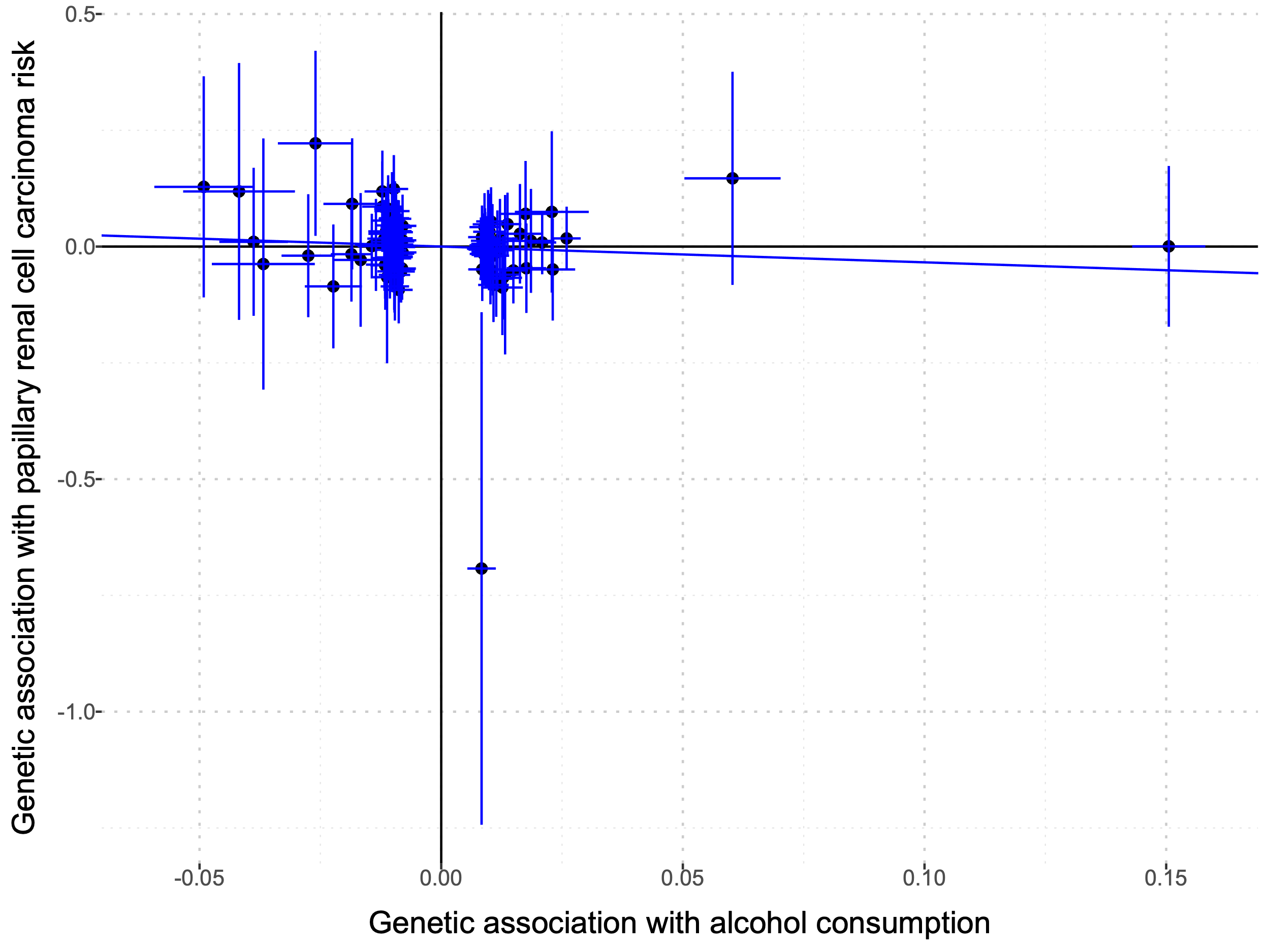


**Fig. S29.** Genetic associations with alcohol consumption and with risk of colorectum cancer from consortium data


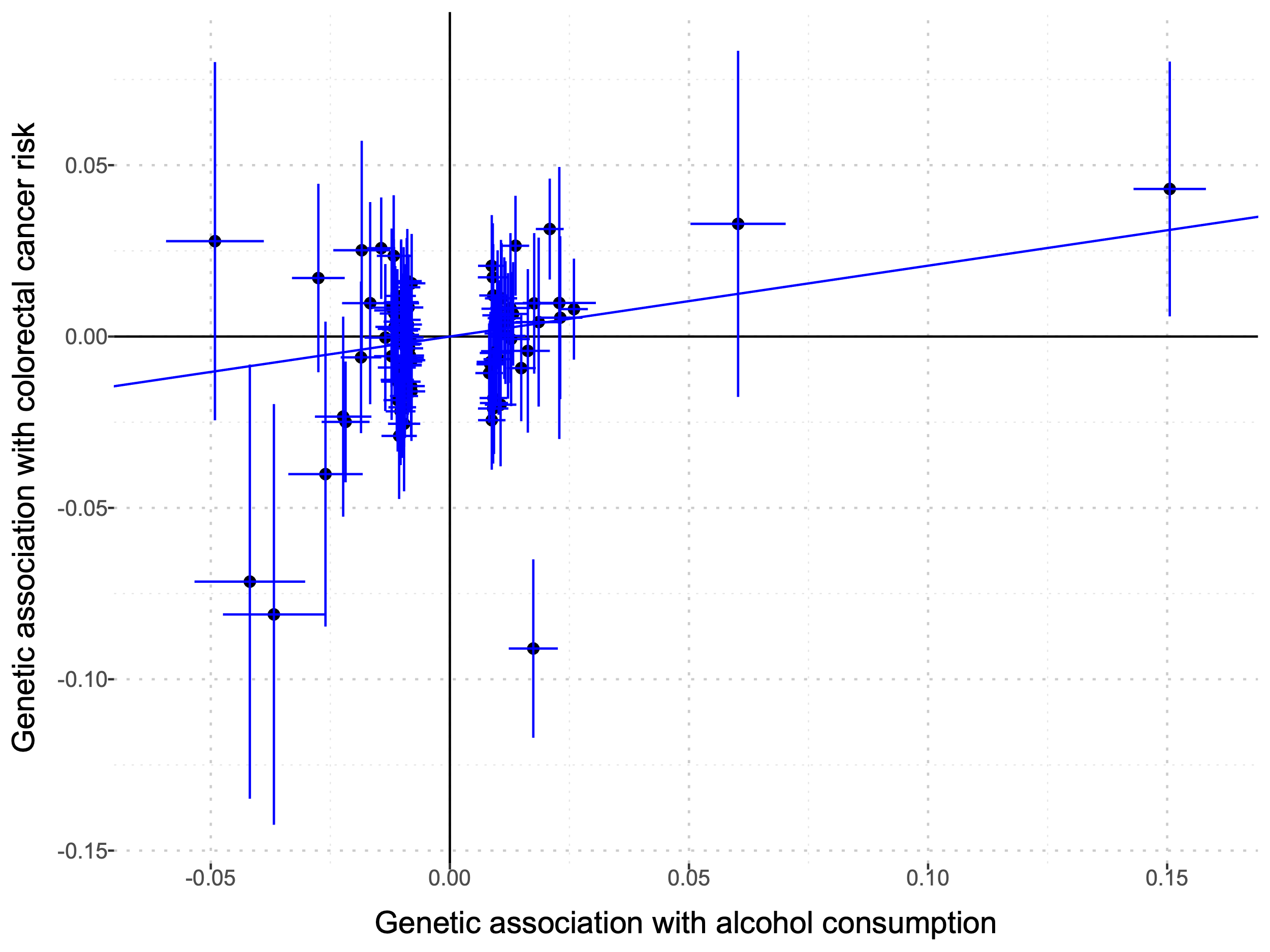


**Fig. S30.** Genetic associations with alcohol consumption and with risk of oesophagus cancer from consortium data


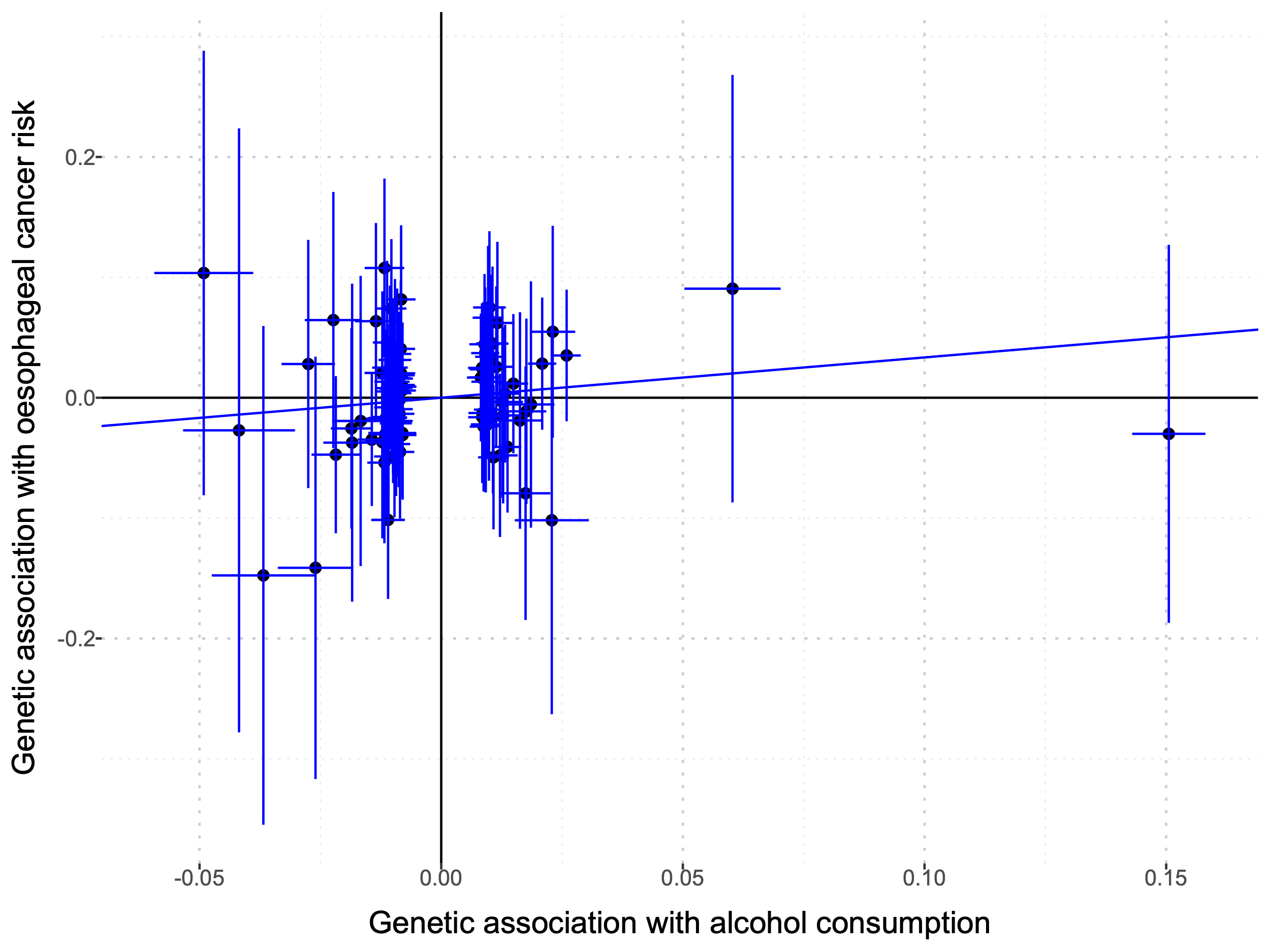


**Fig. S31.** Genetic associations with alcohol consumption and with risk of Barrett's oesophagus from consortium data


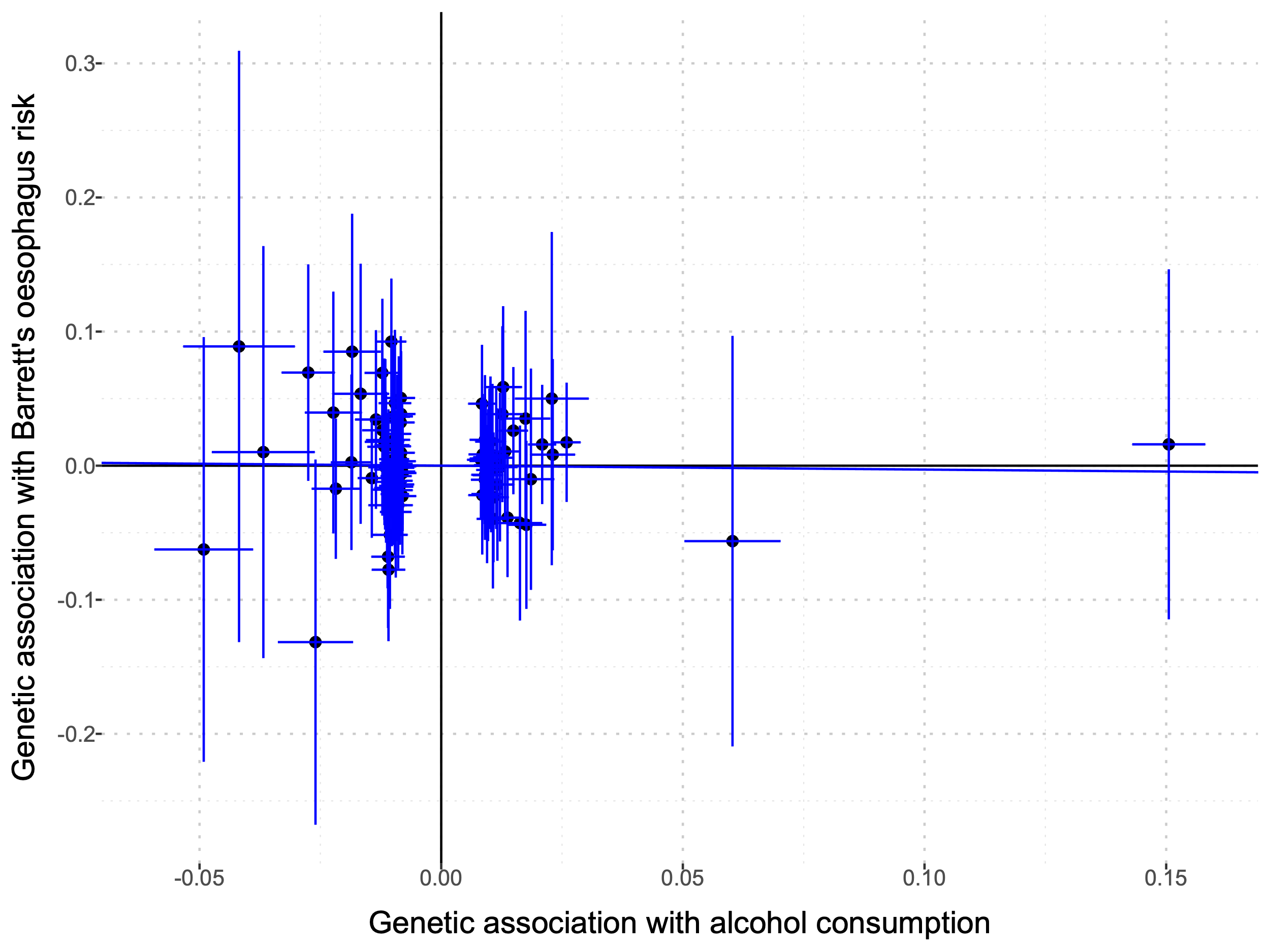

Supplement: Supplementary file 2 — Additional file 2: Figure S1-S31. Fig. S1. Mendelian randomization estimates calculated using outcome data on All of US African ancestry participants. Fig. S2. Mendelian randomization estimates calculated using outcome data on Million Veteran Program African ancestry participants. Fig. S3. Mendelian randomization estimates calculated using outcome data on All of US American admixed ancestry participants. Fig. S4. Mendelian randomization estimates calculated using outcome data on Million Veteran Program American admixed ancestry participants. Fig. S5. Mendelian randomization estimates calculated using outcome data on UK Biobank European ancestry participants. Fig. S6. Mendelian randomization estimates calculated using outcome data on FinnGen participants. Fig. S7. Mendelian randomization estimates calculated using outcome data on All of US European ancestry participants. Fig. S8. Mendelian randomization estimates calculated using outcome data on Million Veteran Program European ancestry participants. Fig. S9. Genetic associations with alcohol consumption and with risk of any breast cancer from consortium data. Fig. S10. Genetic associations with alcohol consumption and with risk of triple negative breast cancer from consortium data. Fig. S11. Genetic associations with alcohol consumption and with risk of triple negative or BRCA+ breast cancer from consortium data. Fig. S12. Genetic associations with alcohol consumption and with risk of luminal A breast cancer from consortium data. Fig. S13. Genetic associations with alcohol consumption and with risk of luminal B breast cancer from consortium data. Fig. S14. Genetic associations with alcohol consumption and with risk of luminal B or HER2- breast cancer from consortium data. Fig. S15. Genetic associations with alcohol consumption and with risk of HER2 enriched breast cancer from consortium data. Fig. S16. Genetic associations with alcohol consumption and with risk of breast cancer survival (Escala-Garcia 2019) from con [file 12916_2025_4543_MOESM2_ESM.docx]
