# Supplementary material for: Alcohol consumption and risk of cancer: a Mendelian randomization analysis of four biobanks and consortium data
Source: BMC Med. 2025 Dec 16;23:676. doi: 10.1186/s12916-025-04543-8 (PMC12707013; doi:10.1186/s12916-025-04543-8)
Supplement: Supplementary file 3 — Additional file 3. STROBE-MR checklist. [file 12916_2025_4543_MOESM3_ESM.docx]

**STROBE-MR checklist of recommended items to address in reports of Mendelian randomization studies**^1^ ^2^

| **Item No.** | **Section** | **Checklist item** | **Page No.** | **Relevant text from manuscript** |
| --- | --- | --- | --- | --- |
| 1 | **TITLE and ABSTRACT** | Indicate Mendelian randomization (MR) as the study’s design in the title and/or the abstract if that is a main purpose of the study | 2 | Title: “Alcohol consumption and risk of cancer: a Mendelian randomization analysis of four biobanks and consortium data”  Abstract: “We perform a comprehensive Mendelian randomization analysis to assess whether genetically-predicted levels of alcohol consumption associate with risk of 20 cancers.” |
|  | **INTRODUCTION** |  |  |  |
| 2 | **Background** | Explain the scientific background and rationale for the reported study. What is the exposure? Is a potential causal relationship between exposure and outcome plausible? Justify why MR is a helpful method to address the study question | 2-3 | Abstract: “Alcohol consumption has been linked to cancer risk. Evidence is strongest for seven cancer types: breast, colorectum, oesophagus, liver, mouth, pharynx, and larynx. However, evidence supporting a causal effect from Mendelian randomization is less consistent.”  Introduction: “However, much of the epidemiological evidence for the causal relationship between alcohol consumption and cancer risk in humans is observational in nature.”  “Mendelian randomization is an epidemiological approach developed to mitigate against bias from confounding and reverse causation that is pervasive in observational research.” |
| 3 | **Objectives** | State specific objectives clearly, including pre-specified causal hypotheses (if any). State that MR is a method that, under specific assumptions, intends to estimate causal effects | 3 | Background: “…to detect a causal effect, as well as specificity to address the particular claims made in the Surgeon General’s report.” |
|  | **METHODS** |  |  |  |
| 4 | **Study design and data sources** | Present key elements of the study design early in the article. Consider including a table listing sources of data for all phases of the study. For each data source contributing to the analysis, describe the following: |  |  |
|  | a) | Setting: Describe the study design and the underlying population, if possible. Describe the setting, locations, and relevant dates, including periods of recruitment, exposure, follow-up, and data collection, when available. | 4-5 | See section “Biobank datasets” |
|  | b) | Participants: Give the eligibility criteria, and the sources and methods of selection of participants. Report the sample size, and whether any power or sample size calculations were carried out prior to the main analysis | 4-5 | See section “Biobank datasets” |
|  | c) | Describe measurement, quality control and selection of genetic variants | 4-5 | See section “Genetic variants used as instruments” |
|  | d) | For each exposure, outcome, and other relevant variables, describe methods of assessment and diagnostic criteria for diseases | 5-7 | See sections “GWAS consortia” and “Outcomes” |
|  | e) | Provide details of ethics committee approval and participant informed consent, if relevant | 15-17 | “This study did not generate any data. Hence, no approval was sought for the study. All original studies from which the data were derived had obtained ethics approval and informed consent from participants." |
| 5 | **Assumptions** | Explicitly state the three core IV assumptions for the main analysis (relevance, independence and exclusion restriction) as well assumptions for any additional or sensitivity analysis | 3 | “The independent segregation of alleles at conception means that these genetically-defined subgroups should not differ systematically with respect to confounding variables, giving rise to a natural experiment analogous to a randomized trial.”  See also Discussion page 12: “The genetic variants may not be valid instrumental variables. They may have pleiotropic effects on other variables that influence alcohol consumption, or they may be subject to bias from assortative mating or population stratification. However, these phenomena would typically bias estimates away from the null, whereas here we observe largely null findings, particularly for more common cancer outcomes.” |
| 6 | **Statistical methods: main analysis** | Describe statistical methods and statistics used | 7 | See section “Statistical methods” |
|  | a) | Describe how quantitative variables were handled in the analyses (i.e., scale, units, model) | 6-7 | See section “Statistical methods” |
|  | b) | Describe how genetic variants were handled in the analyses and, if applicable, how their weights were selected | 6-7 | See section “Genetic variants used as instruments” |
|  | c) | Describe the MR estimator (e.g. two-stage least squares, Wald ratio) and related statistics. Detail the included covariates and, in case of two-sample MR, whether the same covariate set was used for adjustment in the two samples | 7 | See section “Statistical methods”  “Primary Mendelian randomization analyses within each dataset were performed using the inverse-variance weighted method with a random-effects model..”  Covariates: “…adjustment for age, age-squared, sex, and 10 genomic principal components (5 principal components in All of US).” – similar for each dataset. |
|  | d) | Explain how missing data were addressed | 4 | See section “Biobank datasets”:  “We assess genetic associations with cancer risk in four large biobank datasets: UK Biobank, FinnGen, All of US, and Million Veteran Program.”  “While there are non-European ancestry individuals in UK Biobank, they are relatively few in number for any specific ancestry group, and so we do not consider these individuals in our analyses.”  “As the majority of Million Veteran Program participants are male, we do not consider associations with cervical, ovarian, or uterine cancers in this dataset. Additionally, associations in non-European populations were only available for a subset of cancer types.”  All individuals who passed quality control filters were included in our individual-level data analyses. |
|  | e) | If applicable, indicate how multiple testing was addressed | N/A | We did not account for multiple testing. This is stated clearly as a limitation of findings:  “We note that this may lead to false positive claims, as it ignores multiple testing.”  and  “We also did not account for multiple testing in our analyses.” |
| 7 | **Assessment of assumptions** | Describe any methods or prior knowledge used to assess the assumptions or justify their validity | 6-7 | See section “Genetic variants used as instruments” on biological relevance of the genetic variants used as instruments. |
| 8 | **Sensitivity analyses and additional analyses** | Describe any sensitivity analyses or additional analyses performed (e.g. comparison of effect estimates from different approaches, independent replication, bias analytic techniques, validation of instruments, simulations) |  | Comparison of estimates from univariable versus multivariable MR, MR using a single variant, and using the weighted median, MR-Egger, and contamination mixture methods. |
| 9 | **Software and pre-registration** |  |  |  |
|  | a) | Name statistical software and package(s), including version and settings used | 4 | Genome-wide association analyses in MVP were performed using Regenie version 2.2.4  Unless otherwise stated, analyses were performed using R version 4.3.3 (“Angel Food Cake”) and MendelianRandomization package version 0.10.0. |
|  | b) | State whether the study protocol and details were pre-registered (as well as when and where) | N/A | The study protocol was not pre-registered. |
|  | **RESULTS** |  |  |  |
| 10 | **Descriptive data** |  |  |  |
|  | a) | Report the numbers of individuals at each stage of included studies and reasons for exclusion. Consider use of a flow diagram |  | All numbers in Figure 1 and Figure 2 |
|  | b) | Report summary statistics for phenotypic exposure(s), outcome(s), and other relevant variables (e.g. means, SDs, proportions) |  | Description of each study given in the text. |
|  | c) | If the data sources include meta-analyses of previous studies, provide the assessments of heterogeneity across these studies |  | Study-specific estimates are shown in Supplementary Figures 5-8 |
|  | d) | For two-sample MR:  i.  Provide justification of the similarity of the genetic variant-exposure associations between the exposure and outcome samples  ii.  Provide information on the number of individuals who overlap between the exposure and outcome studies |  | ii. “Genetic associations with endometrial cancer were obtained from analyses of female European ancestry participants by O’Mara et al. Associations were estimated in 12,906 European ancestry cases and 108,979 European ancestry controls. This dataset contains 636 cases that overlap with UK Biobank.”  “Genetic associations with prostate cancer were obtained from analyses of male European ancestry participants by Wang et al. Associations were estimated in 122,188 European ancestry cases and 604,640 European ancestry controls. This dataset contains 8765 cases that overlap with UK Biobank, 6311 that overlap with FinnGen, and 13,649 that overlap with Million Veteran Program.”  “Genetic associations with kidney cancer were obtained from Purdue et al, a large GWAS consortium. Associations with any kidney cancer were estimated in 25,890 European ancestry cases and 743,585 European ancestry controls. We also consider associations with clear renal cell carcinoma (16,321 cases) and papillary renal cell carcinoma (2193 cases). These datasets contain around 2900 cases that overlap with UK Biobank and FinnGen.”  “Genetic associations with colorectal cancer were obtained from Fernandez-Rozadilla et al. Associations were estimated in 78,473 European ancestry cases and 107,143 European ancestry controls. This dataset contains 4800 cases that overlap with UK Biobank.” |
| 11 | **Main results** |  |  |  |
|  | a) | Report the associations between genetic variant and exposure, and between genetic variant and outcome, preferably on an interpretable scale |  | Genetic associations with exposure are shown in Supplementary Table 5.  Genetic associations with the exposure and outcome are shown in Supplementary Figures 9-27. |
|  | b) | Report MR estimates of the relationship between exposure and outcome, and the measures of uncertainty from the MR analysis, on an interpretable scale, such as odds ratio or relative risk per SD difference |  | MR estimates are displayed in Figures 1, 2 and Supplementary Figures 1-8 together with confidence intervals.  MR estimates are also provided in the Results section, together with confidence intervals: “In primary analyses across the four biobanks (Figure 1), there was a positive estimate for colorectal cancer (OR 1.21, 95% CI 1.01, 1.45, p=0.035), head/neck cancer (OR 1.51, 95% CI 1.18, 1.93, p=0.001), lung cancer (OR 1.41, 95% CI 1.17, 1.70, p=0.0004), and oesophageal cancer (OR 1.50, 95% CI 1.00, 2.25, p=0.049), and negative estimates for kidney cancer (OR 0.64, 95% CI 0.50, 0.82, p=0.0003), myeloma (OR 0.61, 95% CI 0.41, 0.90, p=0.014), and non-Hodgkin’s lymphoma (OR 0.75, 95% CI 0.61, 0.94, p=0.010).” |
|  | c) | If relevant, consider translating estimates of relative risk into absolute risk for a meaningful time period |  | N/A |
|  | d) | Consider plots to visualize results (e.g. forest plot, scatterplot of associations between genetic variants and outcome versus between genetic variants and exposure) |  | N/A |
| 12 | **Assessment of assumptions** |  |  |  |
|  | a) | Report the assessment of the validity of the assumptions |  | Tables 1 and 2 |
|  | b) | Report any additional statistics (e.g., assessments of heterogeneity across genetic variants, such as *I^2^*, Q statistic or E-value) |  | R^2 statistics are provided in the Results section. |
| 13 | **Sensitivity analyses and additional analyses** |  |  |  |
|  | a) | Report any sensitivity analyses to assess the robustness of the main results to violations of the assumptions |  | Tables 1 and 2. |
|  | b) | Report results from other sensitivity analyses or additional analyses |  | Supplementary Tables S7-S13 |
|  | c) | Report any assessment of direction of causal relationship (e.g., bidirectional MR) |  | N/A, directionality not considered. |
|  | d) | When relevant, report and compare with estimates from non-MR analyses | 11 | Conventional observational analysis results are presented in Table 3, and discussed in the Discussion section |
|  | e) | Consider additional plots to visualize results (e.g., leave-one-out analyses) |  | Scatterplots are presented in Supplementary Figures S9-S27. |
|  | **DISCUSSION** |  |  |  |
| 14 | **Key results** | Summarize key results with reference to study objectives | 11 | Results are summarized in the Discussion section:  “We found no evidence of a harmful effect of alcohol consumption on overall cancer incidence, but the genetic association with cancer mortality was positive. Of the seven cancers listed in the US Surgeon General’s report as convincingly linked to alcohol, we saw evidence supporting a harmful effect of alcohol consumption on head/neck cancer (our definition includes mouth, pharynx, and larynx cancers), oesophageal cancer, and colorectal cancer at a nominal level of statistical significance in the primary analysis. Moreover, we found evidence supporting a harmful effect of alcohol consumption on liver cancer in the analysis utilizing the ADH1B-rs1229984 genetic variant only. Conversely, estimates for breast cancer were compatible with the null in all analyses. The positive associations of genetically-predicted alcohol consumption with cancer mortality, oesophageal cancer, and lung cancer attenuated, and became fully null for lung cancer, upon adjustment for smoking heaviness.”  And in the Abstract: “Out of the seven highlighted cancer types, we saw positive estimates for combined head/neck cancer (OR 1.51, p = 0.001), colorectal cancer (OR 1.21, p=0.035), and oesophageal cancer (OR 1.42 p=0.033). For liver cancer, there was a null estimate overall (OR 1.40, p=0.10), but a positive estimate in Million Veteran Program and when using the ADH1B-rs1229984 genetic variant only. For breast cancer, there was a null estimate in biobank data (OR 1.09, p=0.25) and consortium data (OR 0.98, p=0.84). Otherwise, the only other cancer type with a positive estimate was lung cancer, but this attenuated to the null on adjustment for smoking heaviness. Conversely, we observed negative estimates for kidney cancer (OR 0.65, p=0.0006), endometrial cancer (OR 0.55, p=0.0002), non-Hodgkin’s lymphoma (OR 0.75, p=0.010), myeloma (OR 0.61, p=0.014), and some subtypes of ovarian cancer. There was a positive association with cancer mortality (OR 1.42, p=0.003), although this attenuated on adjustment for smoking heaviness.” |
| 15 | **Limitations** | Discuss limitations of the study, taking into account the validity of the IV assumptions, other sources of potential bias, and imprecision. Discuss both direction and magnitude of any potential bias and any efforts to address them | 11 | Limitations are clearly discussed in the Discussion section, paragraph beginning “There are many caveats and limitations to this research…”  We discuss several potential limitations, including pleiotropy, violation of the assumptions for non-linear MR, population stratification, representativeness and selection bias, availability of genetic variants, measurement error in the exposure, inclusion of prevalent events, and statistical power. |
| 16 | **Interpretation** |  |  |  |
|  | a) | Meaning: Give a cautious overall interpretation of results in the context of their limitations and in comparison with other studies | 11-14 | Interpretation of results is provided in the Discussion section: “We saw evidence supporting a harmful effect of alcohol consumption on head/neck cancer (our definition includes mouth, pharynx, and larynx cancers), oesophageal cancer, and colorectal cancer at a nominal level of statistical significance in the primary analysis.” (and following sentences)  And in Abstract: “We observed moderate-to-weak evidence supporting causal effects of alcohol consumption on risk of head/neck, oesophageal, and colorectal cancer, inconsistent evidence for an effect on liver cancer, and no evidence for an effect on risk of breast cancer.” |
|  | b) | Mechanism: Discuss underlying biological mechanisms that could drive a potential causal relationship between the investigated exposure and the outcome, and whether the gene-environment equivalence assumption is reasonable. Use causal language carefully, clarifying that IV estimates may provide causal effects only under certain assumptions |  | N/A, many competing potential explanations have been considered at length elsewhere. |
|  | c) | Clinical relevance: Discuss whether the results have clinical or public policy relevance, and to what extent they inform effect sizes of possible interventions | 11-14 | Clinical relevance is discussed in the Discussion: “We found no evidence of a harmful effect of alcohol consumption on overall cancer incidence, but the genetic association with cancer mortality was positive.”  “We observed moderate-to-weak evidence supporting causal effects of alcohol consumption on the risk of most “alcohol-related cancers”, including head/neck, oesophageal, colorectal, and liver cancers. No evidence was found to support an effect on breast cancer risk. Alcohol consumption may increase the severity of the consequences of cancer, but human genetics does not provide evidence that it is a cause of all cancers, and suggests it may even protect against some cancers.”  And in the Abstract: “Alcohol consumption may increase the severity of the consequences of cancer, but human genetics does not provide evidence that it is a cause of all cancers, and suggests it may even protect against some cancers.” |
| 17 | **Generalizability** | Discuss the generalizability of the study results (a) to other populations, (b) across other exposure periods/timings, and (c) across other levels of exposure | 13 | Generalizability is discussed in the Discussion “Epidemiological studies typically recruit participants that are healthier than average members of the underlying population, and so heavy drinkers may be underrepresented in our analyses.” |
|  | **OTHER INFORMATION** |  |  |  |
| 18 | **Funding** | Describe sources of funding and the role of funders in the present study and, if applicable, sources of funding for the databases and original study or studies on which the present study is based |  | Provided as part of the submission (subheading “Funding”). |
| 19 | **Data and data sharing** | Provide the data used to perform all analyses or report where and how the data can be accessed, and reference these sources in the article. Provide the statistical code needed to reproduce the results in the article, or report whether the code is publicly accessible and if so, where |  | Provided as part of the submission (subheading “Data availability statement”). |
| 20 | **Conflicts of Interest** | All authors should declare all potential conflicts of interest |  | Provided as part of the submission (subheading “Conflict of Interest”). |

This checklist is copyrighted by the Equator Network under the Creative Commons Attribution 3.0 Unported (CC BY 3.0) license.

1. Skrivankova VW, Richmond RC, Woolf BAR, Yarmolinsky J, Davies NM, Swanson SA, et al. Strengthening the Reporting of Observational Studies in Epidemiology using Mendelian Randomization (STROBE-MR) Statement. JAMA. 2021;326(16):1614-1621.

2. Skrivankova VW, Richmond RC, Woolf BAR, Davies NM, Swanson SA, VanderWeele TJ, et al. Strengthening the Reporting of Observational Studies in Epidemiology using Mendelian Randomisation (STROBE-MR): Explanation and Elaboration. BMJ. 2021;375:n2233.
